# Gene Region Association Analysis of Longitudinal Quantitative Traits Based on a Function-On-Function Regression Model

**DOI:** 10.3389/fgene.2022.781740

**Published:** 2022-02-21

**Authors:** Shijing Li, Shiqin Li, Shaoqiang Su, Hui Zhang, Jiayu Shen, Yongxian Wen

**Affiliations:** ^1^ College of Computer and Information Science, Fujian Agriculture and Forestry University, Fuzhou, China; ^2^> Institute of Statistics and Application, Fujian Agriculture and Forestry University, Fuzhou, China; ^3^ School of Life Science and Technology, ShanghaiTech University, Shanghai, China

**Keywords:** association testing, functional data analysis, longitudinal traits, gene region, rare variants

## Abstract

In the process of growth and development in life, gene expressions that control quantitative traits will turn on or off with time. Studies of longitudinal traits are of great significance in revealing the genetic mechanism of biological development. With the development of ultra-high-density sequencing technology, the associated analysis has tremendous challenges to statistical methods. In this paper, a longitudinal functional data association test (LFDAT) method is proposed based on the function-on-function regression model. LFDAT can simultaneously treat phenotypic traits and marker information as continuum variables and analyze the association of longitudinal quantitative traits and gene regions. Simulation studies showed that: 1) LFDAT performs well for both linkage equilibrium simulation and linkage disequilibrium simulation, 2) LFDAT has better performance for gene regions (include common variants, low-frequency variants, rare variants and mixture), and 3) LFDAT can accurately identify gene switching in the growth and development stage. The longitudinal data of the Oryza sativa projected shoot area is analyzed by LFDAT. It showed that there is the advantage of quick calculations. Further, an association analysis was conducted between longitudinal traits and gene regions by integrating the micro effects of multiple related variants and using the information of the entire gene region. LFDAT provides a feasible method for studying the formation and expression of longitudinal traits.

## 1 Introduction

With sequencing technology development, genome-wide association studies (GWASs) have identified thousands of genetic variants successfully (Robinson et al., 2014). This research plays an important role in identifying the genetic associations of complex traits and diseases. However, GWASs that assess quantitative traits at a single time cannot better reveal the genetic mechanism of biological development. In fact, longitudinal traits have always been a major scientific issue in biology. As early as 1962, Kheiralla and Whittingtom, 1962 found that genetic effects behave differently in different periods. In the eighth decade of the last century, Lewis, 1978 revealed the molecular mechanism of morphological development in Drosophila, which laid a foundation for developing trait developmental genetics. At present, more and more scholars are conducting research on longitudinal traits and exploring the response mechanism of longitudinal traits to genetic variation in the development of crops. (Smith et al., 2010; Cousminer et al., 2013; Tang et al., 2014).

**FIGURE 1:**
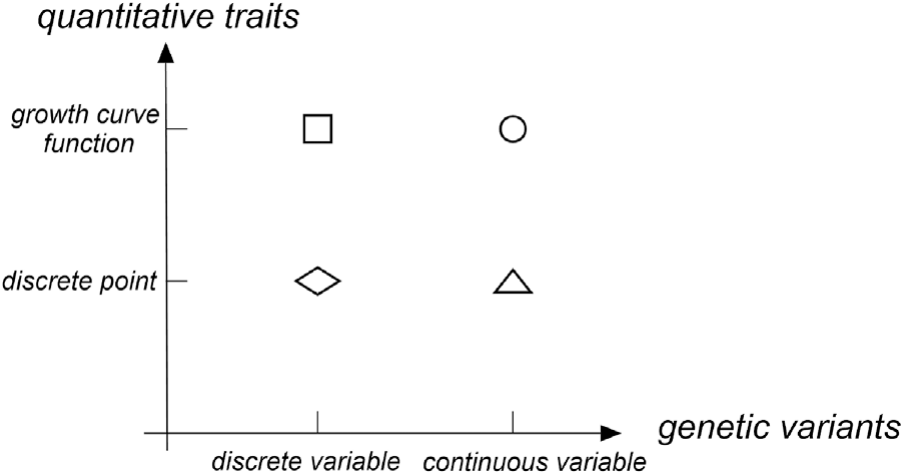
Research status on quantitative traits and genetic variants.

With the development of molecular biotechnology, the position of genes that control phenotypic traits in the genome was determined via a linkage analysis and association analysis to reveal the influence of genetic variations on phenotypic traits. For quantitative traits, many QTL (quantitative trait locus) mapping methods were proposed. Early QTL mapping methods that use a linkage analysis were mainly divided into three types for longitudinal traits: 1) treating phenotypes at different time points as repeated values of one trait; 2) treating phenotypes at different time points as measured values of different traits and analyze them via multiple trait methods; and 3) establishing a model between time points and phenotypes (Zhang, 2006). The former two methods were discrete in both quantitative traits and gene locus directions (as shown with a diamond in Figure 1). The third method fits longitudinal traits to a continuous curve. However, the locus direction still maintained a discrete state (as shown with a square in Figure 1). In the longitudinal data analysis, the third method was most commonly used. Further, several statistical methods had been developed, such as random effects models (Laird and Ware, 1982), hierarchical linear models (Raudenbush and Bryk, 2002), empirical Bayes models (Hui and Berger, 1983), and growth mixture models (Muthen, 2004). Now with the development of GWASs, the above statistical methods have been applied to test the single genetic variant of longitudinal traits via an association analysis. Das et al. (2011) integrated the growth curve describing traits into the GWAS framework and established a functional GWAS model to improve the test power of variants. Fan et al. (2012) proposed temporal association mapping models for longitudinal population data. Both parametric models and nonparametric models were proposed to be applied to multiple diallelic genetic markers. Meanwhile, Meirelles et al. (2013) established a shrinking average model based on the empirical Bayes algorithm. The test power of that dynamic model at multiple time points was significantly increased compared with that of a single time point.

GWASs were mainly divided into two types of studies for quantitative traits: 1) an association analysis based on common variants and 2) and an association analysis based on rare variants. At present, the single-variant association analysis has always been used by GWAS based on common variants mainly. Many methods were proposed in these studies and made great progress. However, the single-variant association analysis was limited to test rare variants common in high-throughput sequencing (Han and Pan, 2010; Sha et al., 2016). Common variants explained only a small part of genetic variation, and most of the associated sites that controlled complex traits were rare variants (Gibson, 2012; Marouli et al., 2017). A single-variant association analysis often ignored the overall information of gene region that rare variants were located. An association analysis method based on gene region can analyze the combination of the effects of the variant sites in the entire gene region. That method reduced the burden of multiple testing and has larger test power (Neale and Sham, 2004; Wu et al., 2010).

Most association analysis methods based on the gene region were designed for the phenotypic traits at a single time point. These methods were mainly divided into three types: 1) the burden test method based on the idea of merging (Madsen and Browning, 2009; Han and Pan, 2010; Morris and Zeggini, 2010; Price et al., 2010; Lin and Tang, 2011), 2) the variance composition method based on mixture effects (Liu et al., 2007; Kwee et al., 2008; Liu et al., 2008; Wu et al., 2010; Wu et al., 2011; Schifano et al., 2012; Chen et al., 2013), and 3) the method based on a functional data analysis (Luo et al., 2012; Svishcheva et al., 2015; Svishcheva et al., 2016a; Svishcheva et al., 2016b; Li et al., 2020). The current functional data analysis method maintained a discrete state in the direction of quantitative traits at a single time point and treated many discrete SNP sequences located in a narrow gene region as continuous variables in the direction of the gene locus. Then, gene regions that contained a large amount of genetic variation were analyzed using a functional data analysis (as shown with a triangle in Figure 1). Many studies have shown that the test power of the functional data analysis method was higher than that of the burden test method based on the combination idea and the variance component method based on the mixture effect (Luo et al., 2012; Fan et al., 2013; Svishcheva et al., 2016a).

The literature on the statistical method of the gene region association analysis for longitudinal traits was limited (Beyene and Hamid, 2014; Wu et al., 2014; Yan et al., 2015; Chien et al., 2016; Cao et al., 2017). In recent studies, a longitudinal trait association test method with the covariates based on SKAT (the sequence kernel association test) method (LSKAT; Longitudinal SNP-set/SKAT) was proposed (Wang et al., 2017). This method combined the features of linear mixed models and kernel machine methods. The association between genetic variation regions and longitudinal traits was analyzed using LSKAT. At the same time, a longitudinal trait burden test (LBT) that tested the association between traits and burden scores in a linear mixed model was proposed in that study. However, the inversion of the correlation matrix was required for these methods, and the calculation of the *p*-value using the eigenvalue decomposition of the correlation matrix brought a computational burden. Simultaneously, the time-varying genetic effect was not considered, but the influence of genes on traits might change over time.

In the functional data analysis, the function-on-function regression model can fit the growth curve of quantitative traits and transform dense discrete gene loci into continuous functions (as shown with a dot in Figure 1). Therefore, we propose a longitudinal functional data association test (LFDAT) method where the function-on-function regression model is applied to detect the association between the gene region and the longitudinal trait. This method can aggregate the small effects distributed at multiple sites, gather the association information of the entire gene region, and improve the test power of related sites with micro effects.

## 2 Methods

### 2.1 Function-On-Function Regression Model

Suppose that there are *n* individuals in a population, and the group structure and other factors are not considered. The SNP sequence constitutes the gene region [0, M] containing the *L* genetic locus, and the growth and development traits are measured in the time period [0, T]. Let $y_{i}\left(t\right)$ denote the phenotype of the *i*th (i=1,2,⋯,n) subject at the time point *t* ( t∈[0,T] ) and $x_{is}$ denote the marker information of the *i*th subject at the *s*th ( s∈[0,M] ) genetic locus. Consider a QTL with two alleles: Q and q. The two alleles can form three genotypes: QQ, Qq, and qq. The value of $x_{is}$ is 2 for QQ, 1 for Qq, and 0 for qq. At the time point *t*, the relationship between the phenotypic trait and the marker information can be described with the following multivariate linear genetic model:

$$y_{i}\left(t\right)=\mu \left(t\right)+\sum_{s=1}^{L}x_{is}\beta_{s}\left(t\right)+\varepsilon_{i}\left(t\right),i=1,2,\cdots ,n$$ (1)

where μ(t) is the population mean, $\varepsilon_{i}\left(t\right)$ is random error following a normal distribution $N\left(0,\sigma^{2}\right)$ , and ρ is the time correlation coefficient between each $\varepsilon_{i}\left(t\right)$ . Further, $\beta_{s}\left(t\right)$ is the genetic effect of the *s*th genetic locus at time point *t*. When the number of genetic markers is infinitely dense, the genetic model of the phenotype $y_{i}\left(t\right)$ can be expressed by a function-on-function regression model:

$$y_{i}\left(t\right)=\mu \left(t\right)+\int_{0}^{M}x_{i}\left(s\right)\beta \left(s,t\right)ds+\varepsilon_{i}\left(t\right),\;t\in \left[0,T\right],\;i=1,2,\cdots ,n.$$ (2)

where $x_{i}\left(s\right)$ is the genetic marker function of the *i*th subject in the gene region [0,M] and β(s,t) is the genetic effect of the *s*th genetic locus at the time point *t*, which is referred to herein as the time-varying function of the genetic effect.

### 2.2 Parameter Estimation

The intercept function μ(t) is dropped by centralization to simplify our discussion of the estimation of the model (2). According to functional data analysis method (Malfait and Ramsay, 2003; Zhao, 2015), let $y_{i}^{*}\left(t\right)=y_{i}\left(t\right)-\bar{y}\left(t\right)$ and $x_{i}^{*}\left(s\right)=x_{i}\left(s\right)-\bar{x}\left(s\right)$ , where $\bar{y}\left(t\right)=\frac{1}{n}\sum_{i=1}^{n}y_{i}\left(t\right)$ and $\bar{x}\left(s\right)=\frac{1}{n}\sum_{i=1}^{n}x_{i}\left(s\right)$ . Then, obtain the following:

$$y_{i}^{*}\left(t\right)=\int_{0}^{M}x_{i}^{*}\left(s\right)\beta \left(s,t\right)ds+\varepsilon_{i}\left(t\right)$$ (3)

The asterisk is dropped in what follows to further simplify the expression.

Let $\hat{\beta }\left(s,t\right)$ be the approximation of β(s,t) . Based on functional data analysis method, so $\hat{\beta }\left(s,t\right)$ can be linearly expressed by *K* known two-dimensional basis functions $\varphi_{k}\left(s,t\right)$ :

$$\hat{\beta }\left(s,t\right)=\sum_{k=1}^{K}b_{k}\varphi_{k}\left(s,t\right)$$ (4)

Combine (4) and (3) to obtain the following:

$$\begin{array}{l} y_{i}\left(t\right)=\int_{0}^{M}x_{i}\left(s\right)\left(\beta \left(s,t\right)-\hat{\beta }\left(s,t\right)\right)+x_{i}\left(s\right)\hat{\beta }\left(s,t\right)ds+\varepsilon_{i}\left(t\right)\\ =\sum_{k=1}^{K}b_{k}\int_{0}^{M}x_{i}\left(s\right)\varphi_{k}\left(s,t\right)ds+\int_{0}^{M}x_{i}\left(s\right)\varepsilon \left(s,t\right)ds+\varepsilon_{i}\left(t\right)\\ =\sum_{k=1}^{K}b_{k}\psi_{ik}\left(t\right)+{\varepsilon^{\prime }}_{i}\left(t\right) \end{array}$$ (5)

where $\psi_{ik}\left(t\right)=\int_{0}^{M}x_{i}\left(s\right)\varphi_{k}\left(s,t\right)ds$ and $\varepsilon \left(s,t\right)=\beta \left(s,t\right)-\hat{\beta }\left(s,t\right)$ . ${\varepsilon^{\prime }}_{i}\left(t\right)$ is the combined error composed of random error $\varepsilon_{i}\left(t\right)$ and approximation error ε(s,t) .

In simplifying the notations, we further obtain the matrix expression of Eq. 5:

y(t)=Ψ(t)b+e(t) (6)

where

$$y\left(t\right)=\left[\begin{array}{c} y_{1}\left(t\right)\\ y_{2}\left(t\right)\\ \vdots \\ y_{n}\left(t\right) \end{array}\right]\;b=\left[\begin{array}{c} b_{1}\\ b_{2}\\ \vdots \\ b_{K} \end{array}\right]\;\psi_{i}\left(t\right)=\left[\begin{array}{c} \psi_{i1}\left(t\right)\\ \psi_{i2}\left(t\right)\\ \cdots \\ \psi_{iK}\left(t\right) \end{array}\right]\;\Psi \left(t\right)=\left[\begin{array}{c} \psi_{1}\left(t\right)\\ \psi_{2}\left(t\right)\\ \vdots \\ \psi_{n}\left(t\right) \end{array}\right]\;e\left(t\right)=\left[\begin{array}{c} {\varepsilon^{\prime }}_{1}\left(t\right)\\ {\varepsilon^{\prime }}_{2}\left(t\right)\\ \vdots \\ {\varepsilon^{\prime }}_{n}\left(t\right) \end{array}\right]$$

The least-square method is used to estimate the coefficient vector b for the sum of integrated squared errors, namely

$$SISE=\int_{0}^{T}\sum_{i=1}^{n}{\left\{{\varepsilon^{\prime }}_{i}\left(t\right)\right\}}^{2}dt$$ (7)

This is equivalent to solving the following equation:

$$\left\{\int_{0}^{T}\Psi^{T}\left(t\right)\Psi \left(t\right)dt\right\}b=\int_{0}^{T}\Psi^{T}\left(t\right)y\left(t\right)dt$$ (8)

We impose a roughness penalty term on the two-dimensional basis function in each dimension separately.

Let $PEN_{1}$ and $PEN_{2}$ denote the roughness penalty in the *s* and *t* directions, respectively.

$$\begin{array}{l} PEN_{1}=\lambda_{1}{\iint \left[\frac{D^{2}\beta \left(s,t\right)}{ds^{2}}\right]}^{2}dsdt\\ =\lambda_{1}{\iint \left[\sum_{k=1}^{K}b_{k}{\varphi^{\prime \prime }}_{k\left(s\right)}\left(s,t\right)\right]}^{2}dsdt\\ =\lambda_{1}\iint {\left[b^{T}\varphi_{1}\left(s,t\right)\right]}^{2}dsdt\\ =\lambda_{1}b^{T}\left\{\iint \left[\varphi_{1}\left(s,t\right)\varphi_{1}^{T}\left(s,t\right)\right]dsdt\right\}b\\ =\lambda_{1}b^{T}R_{1}b \end{array}$$ (9)

where $R_{1}=\iint \left[\varphi_{1}\left(s,t\right)\varphi_{1}^{T}\left(s,t\right)\right]dsdt$ is a K×K matrix, $\lambda_{1}$ is a smoothing parameter and $\varphi_{1}\left(s,t\right)={\left[{\varphi^{\prime \prime }}_{1\left(s\right)}\left(s,t\right),\cdots ,{\varphi^{\prime \prime }}_{K\left(s\right)}\left(s,t\right)\right]}^{T}$ , ${\varphi^{\prime \prime }}_{k\left(s\right)}\left(s,t\right)$ is the second derivative of $\varphi_{k}\left(s,t\right)$ for the direction of *s*.

The same, the matrix expression of $PEN_{2}$ is as follows:

$$\begin{array}{l} PEN_{2}=\lambda_{2}{\iint \left[\frac{D^{2}\beta \left(s,t\right)}{dt^{2}}\right]}^{2}dsdt\\ =\lambda_{2}b^{T}R_{2}b \end{array}$$ (10)

where $R_{2}=\iint \left[\varphi_{2}\left(s,t\right)\varphi_{2}^{T}\left(s,t\right)\right]dsdt$ is a K×K matrix, $\lambda_{2}$ is a smoothing parameter and $\varphi_{2}\left(s,t\right)={\left[{\varphi^{\prime \prime }}_{1\left(t\right)}\left(s,t\right),\cdots ,{\varphi^{\prime \prime }}_{K\left(t\right)}\left(s,t\right)\right]}^{T}$ , ${\varphi^{\prime \prime }}_{k\left(t\right)}\left(s,t\right)$ is the second derivative of $\varphi_{k}\left(s,t\right)$ for the direction of *t*.

Now, we wish to minimize the sum of two penalties and the sum of integrated squared errors, expressed as follows:

$$PENSISE=SISE+PEN_{1}+PEN_{2}$$ (11)

This is equivalent to solving the following equation:

$$\left\{\int_{0}^{T}\Psi^{T}\left(t\right)\Psi \left(t\right)dt+\lambda_{1}R_{1}+\lambda_{2}R_{2}\right\}b=\int_{0}^{T}\Psi^{T}\left(t\right)y\left(t\right)dt$$ (12)

We evaluate $y_{i}\left(t\right)$ and $\psi_{i1}\left(t\right),\psi_{i2}\left(t\right),\cdots ,\psi_{iK}\left(t\right)$ at a set of time points $\left\{t_{q}\text{|}q=0,1,\cdots ,Q\right\}$ .

Let

$$y_{i}=\left[\begin{array}{c} y_{i}\left(t_{0}\right)\\ y_{i}\left(t_{1}\right)\\ \vdots \\ y_{i}\left(t_{Q}\right) \end{array}\right]\;,\;y=\left[\begin{array}{c} y_{1}\\ y_{2}\\ \cdots \\ y_{n} \end{array}\right],\;\Psi_{i}=\left[\begin{array}{ccccc} \psi_{i1}\left(t_{0}\right) & \cdots & \psi_{ik}\left(t_{0}\right) & \cdots & \psi_{iK}\left(t_{0}\right)\\ \vdots & \ddots & \vdots & \ddots & \vdots \\ \psi_{i1}\left(t_{Q}\right) & \cdots & \psi_{ik}\left(t_{Q}\right) & \cdots & \psi_{iK}\left(t_{Q}\right) \end{array}\right],\;\mathrm{\Psi =}\left[\begin{array}{c} \Psi_{1}\\ \Psi_{2}\\ \cdots \\ \Psi_{n} \end{array}\right]$$

Then, the regular equation of the least square method can be obtained from Eq. 12

$$\left\{\Psi^{T}\Psi +\lambda_{1}R_{1}+\lambda_{2}R_{2}\right\}b=\Psi^{T}y$$ (13)

Finally, the least square estimate of the coefficient vector in Eq. 13 is as follows:

$$\hat{b}={\left\{\Psi^{T}\Psi +\lambda_{1}R_{1}+\lambda_{2}R_{2}\right\}}^{-1}\Psi^{T}y$$ (14)

### 2.3 Hypothesis Testing Based on the Function-On-Function Regression Model

We usually consider the following hypothesis testing to detect whether the association between the gene regions and the phenotypes exists:

$$\begin{array}{ccc} H_{0}:\beta \left(s,t\right)=0; & H_{1}:\beta \left(s,t\right)\neq 0, & \text{for}\;\text{any}\;s,\;s\in \left[0,M\right],t\in \left[0,T\right] \end{array}$$

Since the time-varying function of the genetic effect $\beta \left(s,t\right)=\sum_{k=1}^{K}b_{k}\varphi_{k}\left(s,t\right)$ is a linear combination of two-dimensional basis functions, the above hypothesis testing is equivalent to the following:

$$\begin{array}{ccc} H_{0}:\text{for}\;\text{any}\;b_{k}\;\text{is}\;0; & H_{1}:\text{not}\;\text{all}\;b_{k}\;\text{is}\;\text{0}, & k=1,2,\cdots ,K. \end{array}$$

The following test statistics are available for the above hypothesis testing:

$$F=\frac{\left(RSS_{0}-RSS_{1}\right)/K}{RSS_{1}/\left(n-K-1\right)}∼F\left(K,n-K-1\right)$$ (15)

where $RSS_{0}=\sum_{i=1}^{n}y_{i}^{2}\left(t\right)$ and $RSS_{1}==\sum_{i=1}^{n}{\left(y_{i}\left(t\right)-\psi_{i}\left(t\right)\hat{b}\right)}^{2}$ are the sums of the squared residuals under the null model and the alternative model, respectively.

### 2.4 The Evaluation Indicators of the Estimation Result of the Time-Varying Function of the Genetic Effect

This was done to further measure the fitness of LFDAT for the time-varying function of the genetic effect in the gene region and provide other reference indicators for LFDAT in the process of the association analysis of the longitudinal trait. Then, some evaluation indicators are established for LFDAT.

Let N(β) denote the null region of β(s,t) , and S(β) denote the non-null region of β(s,t) , which is defined as the following:

N(β)={(s,t)∈[0,M]×[0,T]:β(s,t)=0}

and

S(β)={(s,t)∈[0,M]×[0,T]:β(s,t)≠0}.

From a statistical genetic point of view, if the time-varying function of the genetic effect β(s,t) is null, the *s*th genetic locus at the time point *t* is not associated with the longitudinal trait.

For the null region and non-null region, as Lin et al. (2017) and Centofanti et al. (2020) noted, we consider the integrated squared errors (ISE) as the fitting criterion for the estimator β(s,t) . The ISE over the null region (ISE0) and the non-null region (ISE1) are defined as follows:

$$\mathrm{ISE}0=\frac{1}{A_{0}}\int \int_{N\left(\beta \right)}{\left(\hat{\beta }\left(s,t\right)-\beta \left(s,t\right)\right)}^{2}dsdt,$$

and

$$\mathrm{ISE}1=\frac{1}{A_{1}}\int \int_{S\left(\beta \right)}{\left(\hat{\beta }\left(s,t\right)-\beta \left(s,t\right)\right)}^{2}dsdt.$$

where $A_{0}$ and $A_{1}$ are the measures of the null and non-null regions, respectively. The ISE0 and ISE1 are the measures of integrated squared errors between the true function β(s,t) and an estimated function $\hat{\beta }\left(s,t\right)$ on the null and non-null regions, respectively.

The predictive performance is measured by prediction mean squared errors (PMSE), defined as the following:

$$\mathrm{PMSE}=\frac{1}{N}\sum_{\left(x,y\right)\in test}\int_{0}^{T}{\left(y\left(t\right)-\hat{\mu }\left(t\right)-\int_{0}^{M}x\left(s\right)\hat{\beta }\left(s,t\right)ds\right)}^{2}dt.$$

where *test* denotes the test sample data set, *N* is the size of the test sample data set, and $\hat{\mu }\left(t\right)$ is the estimated intercept μ(t) .

In the gene region, we define ISE0, ISE1, and PMSE as the criterion to measure the fitness of the time-varying effect function. ISE0 is used to measure the fitness of the null effect, which denotes the overall deviation between the true value and the estimated value at the site where there is no effect value in the gene region. The expression is as follows:

$$\mathrm{ISE0}=\frac{1}{\left(\left|\Gamma \right|-1\right)\times \left(\left|S_{0}\right|-1\right)}\sum_{t\in \Gamma }\sum_{s\in S_{0}}{\left(\hat{\beta }\left(s,t\right)-\beta \left(s,t\right)\right)}^{2}$$ (16)

where $S_{0}$ denotes a collection of SNP sites that do not have an association relationship in the region, $\left|S_{0}\right|$ represents the number of elements in the collection $S_{0}$ , Γ denotes the collection of measurement time points, |Γ| represents the number of elements in the collection Γ , and β(s,t) and $\hat{\beta }\left(s,t\right)$ represent the actual effect and estimated effect of the *s*th genetic locus at time point *t* in the collection $S_{0}\times \Gamma$ , respectively.

ISE1 is used to measure the fitness of the non-null effect, which denotes the overall deviation between the true value and the estimated value at the site where there is an effect value in the gene region. The expression is as follows:

$$\mathrm{ISE1}=\frac{1}{\left(\left|\Gamma \right|-1\right)\times \left(\left|S_{1}\right|-1\right)}\sum_{t\in \Gamma }\sum_{s\in S_{1}}{\left(\hat{\beta }\left(s,t\right)-\beta \left(s,t\right)\right)}^{2}$$ (17)

where $S_{1}$ denotes a collection of SNP sites with an association relationship in the region, $\left|S_{1}\right|$ represents the number of elements in the collection $S_{1}$ , and β(s,t) and $\hat{\beta }\left(s,t\right)$ represent the actual effect and estimated effect of the *s*th genetic locus at time point *t* in the collection $S_{1}\times \Gamma$ , respectively.

PMSE is used to measure the fitness of the genetic model, which denotes the overall deviation between the estimated value of the trait obtained fitted by the model and the true value of the trait in the test set. The expression is as follows:

$$\mathrm{PMSE}=\frac{1}{N-1}\sum_{y_{i}\left(t\right)\in test}\sum_{t\in \Gamma }{\left(y_{i}\left(t\right)-{\hat{y}}_{i}\left(t\right)\right)}^{2}$$ (18)

where test denotes the test sample data set, *N* is the size of test sample data set, $y_{i}\left(t\right)$ denotes the true value of the trait of the *i*th subject in test data set at time point *t*, and ${\hat{y}}_{i}\left(t\right)$ denotes the predictive value of the trait of the *i*th subject in test data set at time point *t*.

## 3 Simulation Studies

The SNP sequence data set generated by the computer is used for type I error simulation and power simulation to evaluate the feasibility of the LFDAT. In Luo et al. (2012), the FLM and the Smoothed FLM are proposed for test association between gene region and quantitative trait. Because both the Smoothed FLM and LFDAT have the smooth penalty, so we compare the power of the Smoothed FLM to that of the LFDAT in simulation. However, the Smoothed FLM is only applicable to a single measurement, it’s applied to detect association between gene region and trait at each time point.

In the simulation, we consider linkage equilibrium simulation and linkage disequilibrium simulation. The SNP sequence data set simulated contains a 50 kb gene region, and a 1 kb genetic subregion is randomly selected from the gene region to assess type I error rates and power. The sizes of the samples are 1,000, 1,500, and 2,000, respectively. Gene regions consider five cases: 1) gene regions only have common variants, 2) gene regions only have rare variants, 3) gene regions only have low-frequency variants, 4) gene regions are randomly composed of 20% common variants and 80% rare variants, and 5) gene regions are randomly composed of 80% common variants and 20% rare variants. In the simulation, the upper limit *b* and lower limit *a* of U (*a*, *b*) corresponding to the MAF (minor allele frequency) of gene regions are different. The gene regions of rare variants are (0.0005, 0.01), gene regions of low-frequency variants are (0.01, 0.05), and gene regions of common variants are (0.05, 0.5).

The codes used in this paper are the *linmod* function in the *fda* package of the R software (Ramsay et al., 2009). In the simulation, set the number of two-dimensional B-spline basis functions *K* to 15 and the order *d* to 4. Leave-one-out cross-validation (Ramsay et al., 2009) can be used to select the optimal parameter from a set of smoothing coefficients $\left[10^{2},10^{3},10^{4},10^{5},10^{6}\right]$ for $\lambda_{1}$ and $\lambda_{2}$ .

Due to space limitations, all the simulated results are attached to Supplementary Datas S1–S6.

### 3.1 Linkage Equilibrium Simulation

#### 3.1.1 Type I Error Rates

We use the following model to generate phenotype data to assess type I error rates of LFDAT:

$$y_{i}\left(t\right)=\mu \left(t\right)+\varepsilon_{i}\left(t\right),\;\;\;\;\;i=1,2,\cdots ,n$$

where t=1,2,⋯,9 , μ(t)=1 , $\varepsilon_{i}\left(t\right)∼N\left(0,1\right)$ and the time correlation coefficient between each random error ρ is 0.5. We randomly selected a 1 kb subregion from the SNP sequence data set as the genotype data of the gene regions. Notice that the null hypothesis is valid, and the phenotypes have nothing to do with the current genotypes.

A total of 1,000 genotype-phenotype data sets for each sample size were simulated. The test statistics and related *p*-value based on the above genetic model were calculated. Under a given significance level *α*, the ratio of genotype-phenotype data sets that *p*-value is less than *α* is regarded as a type I error rate.

All results of type I error rates simulation can be seen Supplementary Data S1. Table 1 shows the type I error rates of the LFDAT and Smoothed FLM at the significance level of 0.05, 0.01, and 0.001 for linkage equilibrium simulation. It can be seen that LFDAT controls the type I error rates at each level of significance. The type I error rates of rare gene regions and low-frequency gene regions are lower than that of common gene regions. The type I error rates of gene regions with more common variants are generally higher than those with less common variants. As the significance level increases, the type I error rates of gene regions gradually decrease. For smaller significance levels (*α* is 1e-4, 1e-5, and 1e-6), LFDAT still performs well, and the type I error rates are all 0 (See Supplementary Data S1). Compared with the type I error rates of the LFDAT, the type I error rates of the Smoothed FLM is severely inflated. It means that there are more false associated gene regions with quantitative trait using the Smoothed FLM method. Simulation studies have shown that association analysis which combines the multiple measurement of quantitative traits can reduce the type I error rates.

**TABLE 1:** Type I error rates of the LFDA and Smoothed FLM based on 1,000 simulated replicates for linkage equilibrium simulation.

α	Sample size	Gene region	LFDA									Smoothed FLM								
			t = 1	t = 2	t = 3	t = 4	t = 5	t = 6	t = 7	t = 8	t = 9	t = 1	t = 2	t = 3	t = 4	t = 5	t = 6	t = 7	t = 8	t = 9
0.05	1,000	Common	0.025	0.008	0.011	0.012	0.008	0.006	0.011	0.010	0.027	0.063	0.043	0.057	0.057	0.053	0.049	0.061	0.048	0.057
		Rare	0.013	0.007	0.005	0.003	0.004	0.002	0.001	0.005	0.012	0.050	0.056	0.057	0.048	0.058	0.053	0.046	0.048	0.056
		Low	0.014	0.012	0.005	0.004	0.004	0.006	0.005	0.004	0.016	0.055	0.059	0.055	0.047	0.045	0.041	0.053	0.053	0.057
		Mixture one	0.019	0.007	0.008	0.006	0.000	0.007	0.005	0.016	0.018	0.062	0.048	0.056	0.050	0.049	0.061	0.056	0.058	0.059
		Mixture two	0.021	0.011	0.006	0.010	0.008	0.009	0.006	0.010	0.017	0.054	0.053	0.040	0.048	0.059	0.059	0.060	0.052	0.052
	1,500	Common	0.025	0.009	0.002	0.010	0.005	0.009	0.007	0.009	0.027	0.058	0.048	0.044	0.061	0.035	0.045	0.049	0.053	0.054
		Rare	0.012	0.004	0.000	0.002	0.000	0.003	0.005	0.002	0.007	0.053	0.038	0.057	0.045	0.043	0.040	0.049	0.040	0.046
		Low	0.019	0.006	0.011	0.008	0.002	0.006	0.004	0.013	0.017	0.060	0.042	0.063	0.050	0.064	0.055	0.052	0.062	0.059
		Mixture one	0.013	0.010	0.006	0.002	0.000	0.008	0.002	0.006	0.021	0.062	0.060	0.044	0.044	0.047	0.045	0.040	0.036	0.054
		Mixture two	0.031	0.014	0.009	0.012	0.005	0.007	0.010	0.010	0.028	0.061	0.073	0.050	0.053	0.046	0.049	0.045	0.045	0.054
	2000	Common	0.022	0.009	0.009	0.005	0.005	0.009	0.011	0.007	0.020	0.047	0.041	0.044	0.038	0.048	0.057	0.053	0.044	0.039
		Rare	0.011	0.013	0.002	0.003	0.005	0.004	0.004	0.007	0.010	0.053	0.058	0.046	0.043	0.045	0.040	0.054	0.044	0.037
		Low	0.013	0.011	0.009	0.011	0.002	0.008	0.009	0.010	0.011	0.053	0.059	0.059	0.053	0.050	0.053	0.046	0.046	0.041
		Mixture one	0.023	0.005	0.011	0.004	0.007	0.004	0.009	0.015	0.014	0.049	0.053	0.059	0.051	0.047	0.046	0.051	0.047	0.048
		Mixture two	0.024	0.010	0.009	0.006	0.010	0.012	0.016	0.006	0.032	0.045	0.052	0.056	0.055	0.051	0.055	0.069	0.049	0.062
0.01	1,000	Common	0.003	0.001	0.001	0.001	0.002	0.000	0.001	0.002	0.006	0.009	0.008	0.011	0.013	0.012	0.011	0.014	0.011	0.012
		Rare	0.001	0.000	0.000	0.000	0.000	0.001	0.000	0.002	0.001	0.014	0.008	0.013	0.011	0.009	0.009	0.011	0.012	0.012
		Low	0.003	0.002	0.000	0.001	0.000	0.000	0.001	0.000	0.004	0.011	0.016	0.009	0.009	0.007	0.012	0.012	0.010	0.011
		Mixture one	0.003	0.001	0.000	0.001	0.000	0.000	0.000	0.001	0.002	0.012	0.012	0.012	0.015	0.007	0.012	0.010	0.014	0.010
		Mixture two	0.001	0.000	0.000	0.000	0.001	0.001	0.000	0.001	0.005	0.010	0.008	0.010	0.010	0.011	0.020	0.007	0.011	0.011
	1,500	Common	0.005	0.002	0.000	0.001	0.001	0.000	0.000	0.002	0.003	0.009	0.009	0.002	0.015	0.006	0.008	0.011	0.007	0.008
		Rare	0.002	0.000	0.000	0.000	0.000	0.000	0.001	0.000	0.000	0.013	0.006	0.005	0.008	0.007	0.009	0.013	0.006	0.009
		Low	0.003	0.000	0.001	0.000	0.000	0.002	0.000	0.001	0.003	0.015	0.010	0.020	0.012	0.010	0.015	0.007	0.012	0.012
		Mixture one	0.002	0.003	0.001	0.001	0.000	0.000	0.000	0.001	0.001	0.010	0.010	0.009	0.004	0.004	0.014	0.005	0.008	0.010
		Mixture two	0.006	0.001	0.001	0.001	0.000	0.002	0.002	0.002	0.004	0.012	0.019	0.011	0.016	0.010	0.011	0.009	0.012	0.009
	2000	Common	0.003	0.000	0.001	0.000	0.000	0.000	0.003	0.002	0.008	0.004	0.011	0.009	0.007	0.011	0.011	0.014	0.005	0.010
		Rare	0.002	0.002	0.000	0.000	0.000	0.000	0.000	0.001	0.001	0.011	0.019	0.005	0.013	0.012	0.009	0.008	0.011	0.008
		Low	0.001	0.001	0.000	0.001	0.000	0.001	0.000	0.002	0.001	0.008	0.008	0.013	0.018	0.006	0.012	0.015	0.006	0.005
		Mixture one	0.005	0.000	0.002	0.002	0.000	0.000	0.002	0.000	0.002	0.014	0.013	0.019	0.008	0.011	0.008	0.010	0.018	0.006
		Mixture two	0.006	0.002	0.000	0.002	0.001	0.002	0.003	0.001	0.004	0.012	0.014	0.010	0.013	0.013	0.019	0.018	0.009	0.010
0.001	1,000	Common	0.000	0.000	0.000	0.000	0.000	0.000	0.000	0.002	0.000	0.000	0.002	0.001	0.001	0.002	0.000	0.002	0.002	0.002
		Rare	0.000	0.000	0.000	0.000	0.000	0.000	0.000	0.000	0.000	0.000	0.000	0.001	0.000	0.000	0.001	0.000	0.004	0.001
		Low	0.000	0.000	0.000	0.000	0.000	0.000	0.000	0.000	0.000	0.000	0.002	0.000	0.001	0.001	0.000	0.001	0.000	0.002
		Mixture one	0.000	0.000	0.000	0.000	0.000	0.000	0.000	0.000	0.000	0.001	0.000	0.000	0.001	0.000	0.002	0.000	0.000	0.000
		Mixture two	0.000	0.000	0.000	0.000	0.000	0.000	0.000	0.000	0.001	0.000	0.000	0.000	0.001	0.001	0.002	0.000	0.001	0.001
	1,500	Common	0.001	0.000	0.000	0.000	0.000	0.000	0.000	0.000	0.000	0.001	0.003	0.000	0.001	0.001	0.000	0.000	0.000	0.000
		Rare	0.000	0.000	0.000	0.000	0.000	0.000	0.000	0.000	0.000	0.003	0.000	0.000	0.002	0.000	0.000	0.000	0.000	0.000
		Low	0.000	0.000	0.000	0.000	0.000	0.000	0.000	0.000	0.000	0.002	0.001	0.001	0.001	0.000	0.002	0.001	0.003	0.000
		Mixture one	0.000	0.000	0.001	0.000	0.000	0.000	0.000	0.000	0.000	0.001	0.001	0.001	0.001	0.000	0.001	0.001	0.001	0.000
		Mixture two	0.000	0.000	0.000	0.000	0.000	0.001	0.000	0.000	0.000	0.000	0.001	0.001	0.001	0.000	0.002	0.003	0.002	0.000
	2000	Common	0.000	0.000	0.000	0.000	0.000	0.000	0.000	0.001	0.001	0.000	0.001	0.001	0.000	0.000	0.001	0.003	0.002	0.003
		Rare	0.000	0.000	0.000	0.000	0.000	0.000	0.000	0.000	0.000	0.001	0.002	0.000	0.001	0.001	0.000	0.000	0.001	0.001
		Low	0.000	0.000	0.000	0.000	0.000	0.000	0.000	0.000	0.000	0.000	0.002	0.001	0.001	0.000	0.002	0.001	0.004	0.000
		Mixture one	0.000	0.000	0.000	0.000	0.000	0.000	0.000	0.000	0.000	0.001	0.000	0.001	0.003	0.000	0.000	0.002	0.000	0.000
		Mixture two	0.001	0.000	0.000	0.000	0.000	0.000	0.000	0.000	0.000	0.002	0.001	0.000	0.000	0.000	0.001	0.002	0.000	0.000

Note: Common denotes gene regions only with common variants, Rare denotes gene regions only with rare variants, Low denotes gene regions only with low-frequency variants, Mixture one denotes gene regions with 20% of common variants and 80% of rare variants, and Mixture two denotes gene regions with 80% of common variants and 20% of rare variants.

#### 3.1.2 Power

We randomly selected a 1 kb subregion from the SNP sequence data set under the alternative hypothesis as the genotype data of the variant region to measure the test power of LFDAT for the gene regions. The generate phenotypic data is based on the following model:

$$y_{i}\left(t\right)=\mu \left(t\right)+\sum_{s\in A}x_{is}\beta_{s}\left(t\right)+\varepsilon_{i}\left(t\right)$$

where $x_{is}$ is the genotype of the *i*th subject in *s*th genetic locus, A denotes the collection of causal variants in simulated gene regions, $\beta_{s}\left(t\right)$ is the genetic effect in *s*th variant at time point *t*, t=1,2,⋯,9 , μ(t)=1 , $\varepsilon_{i}\left(t\right)∼N\left(0,1\right)$ , and the time correlation coefficient between each random error ρ is 0.5.

Consider the following scenarios for simulations: 1) the proportion of causal variants in the gene regions is 1, 2, or 4%, and 2) the proportion of negative effects of causal variants is 0, 20, 50%. Various processes in life activities are always accompanied by the selective opening and closing of different genes, and some genes are selectively expressed at a certain stage of development. Based on this phenomenon, the following two cases were considered for the time-varying function of the genetic effect:

Case one. The time-varying function of the genetic effect is β(s,t)=η(s)⋅θ(t) , where $\eta \left(s\right)=\ln\left(c\right)\times |\log_{10}\left(MAF_{s}\right)|/2$ (Wu et al., 2011; Lee et al., 2012; Chen et al., 2013; Fan et al., 2013; Belonogova et al., 2018) is the genetic effect function and θ(t)=2+2⁡sin(πt/12) is the time effect function. Then, $MAF_{s}$ is the minor allele frequency of *s*th genetic locus. The constant *c* will directly affect the size of the genetic effect function, which is set to 3, 5, or 7 in the simulation.
Case two. The time-varying function of the genetic effect is β(s,t)=η(s)⋅θ(t) , where $\eta \left(s\right)=\ln\left(c\right)\times |\log_{10}\left(MAF_{s}\right)|/2$ is the genetic effect function and θ(t)=2+2⁡sin(πt/2) is the time effect function.

For each setting scenario, 1,000 genotype-phenotype data sets are simulated. At the given significance level *α*, the ratio of genotype-phenotype data sets with a *p*-value is less than *α* are used as power. For each genotype-phenotype data set, the variation area is the same for all individuals in the data set. However, we allow the variation of different data sets to be different.

##### Case One Simulation

We assess the test power of five gene regions under different sample sizes (*n* = 1,000, 1,500, 2,000) by LFDAT, and the features that result are the same for each sample size. All results of power simulation can be seen Supplementary Data S1.

**FIGURE 2:**
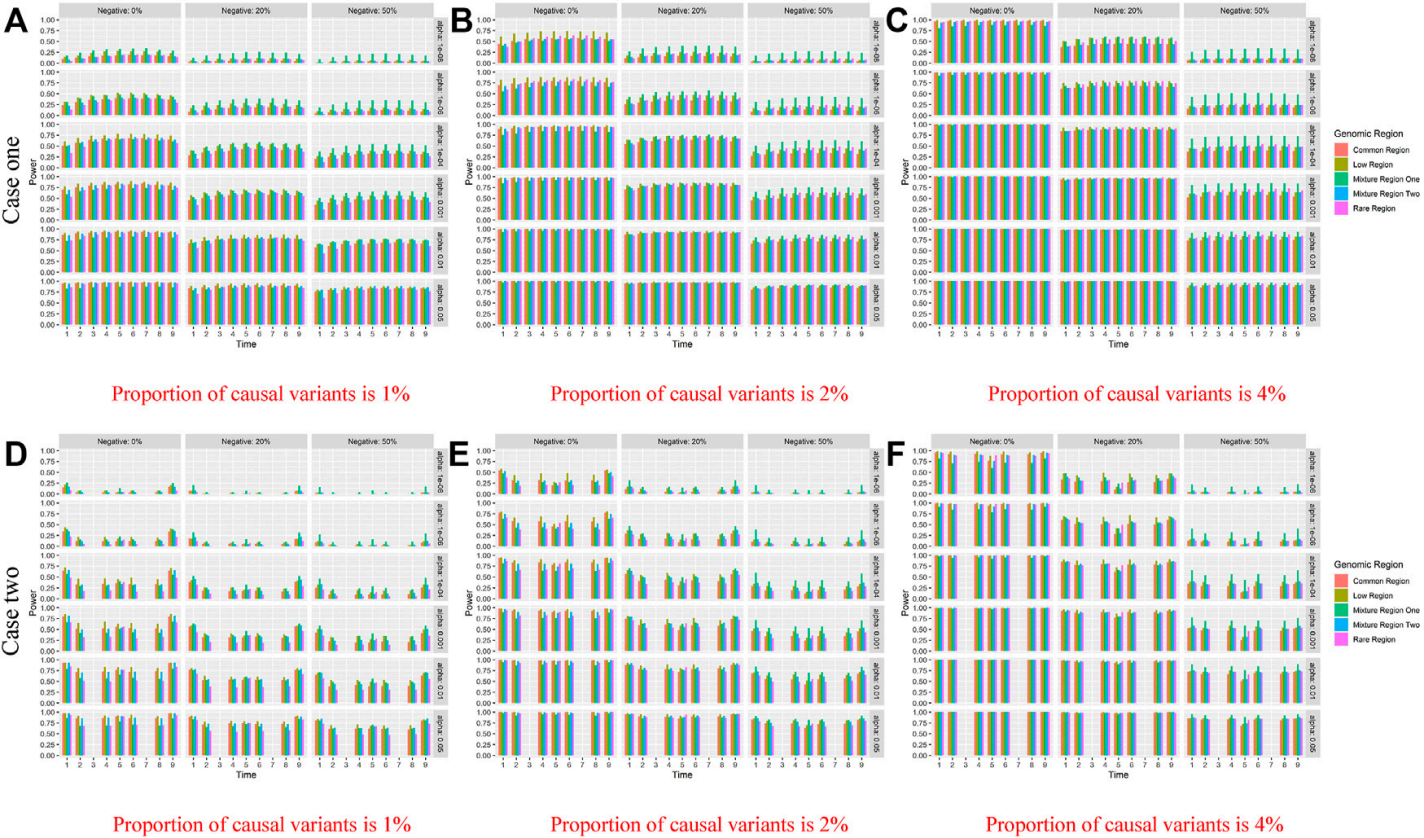
Power of linkage equilibrium’s case one and case two based on LFDAT for the five gene regions when *c* is 3, and sample size is 2000. The **(A–C)** denotes the power results of case one. The **(D–F)** denotes the power results of case two. The time effect function is θ(t)=2+2⁡sin(πt/12) for case one, and θ(t)=2+2⁡sin(πt/2) for case two. **(A)** Proportion of causal variants is 1% **(B)** Proportion of causal variants is 2% **(C)** Proportion of causal variants is 4%. **(D)** Proportion of causal variants is 1% **(E)** Proportion of causal variants is 2% **(F)** Proportion of causal variants is 4%. Note: Common region denotes gene regions only with common variants, Rare region denotes gene regions only with rare variants, Low region denotes gene regions only with low-frequency variants, Mixture region one denotes gene regions with 20% of common variants and 80% of rare variants, and the Mixture region two denotes gene regions with 80% of common variants and 20% of rare variants.

The figures of power based on LFDAT at nine time points are also shown in Supplementary Data S1 for different significance levels, constant *c*, the proportion of negative effects, and causal variants. Figure 2 (Only show the power figures when *c* is 3 and a sample size is 2000) show that the power of each time point is different, which might be the unequal value of the time-varying effect function at each time point. As constant *c* (See Supplementary Data S1) and the proportion of causal variants increase, the power also increases. However, as the proportion of negative effects and significance levels increase, the power gradually decreases. Overall, the power of the five gene regions is higher. We find that whether it is common, rare or low-frequency variants, as the genetic effects increase, the power of testing gene region increases by simulation study. The proportion of negative effects has a smaller impact on the power of mixture gene region one than on the other four gene regions. It may be that the effect values of rare variants are larger than that of common variants, and the offset effects of mixture gene region one are not as much as other regions. The LFDAT is applicable to common variants, rare variants and low-frequency variants.

At the same time, we also compare the power of the LFDAT and Smoothed FLM (See Supplementary Data S1). As the sample size increases, the power increases. In Table 2 (Due to space limitations, results of power are shown when significance level is 0.05, sample is 2000, *c* is 7, and proportion of causal variants is 1%), we can see that the power of the LFDAT is very close to that of the Smoothed FLM. These results indicate that LFDAT can reduce the probability of making the type I errors with keeping its high power.

**TABLE 2:** The power of linkage equilibrium simulation based on LFDA and Smoothed FLM at significance level of 0.05 when sample size is 2000, *c* is 7 and proportion of causal variants is 1%.

	Proportion of negative effects (%)	Gene region	LFDA									Smoothed FLM								
			t = 1	t = 2	t = 3	t = 4	t = 5	t = 6	t = 7	t = 8	t = 9	t = 1	t = 2	t = 3	t = 4	t = 5	t = 6	t = 7	t = 8	t = 9
Case 1	0	Common	0.982	0.987	0.990	0.989	0.989	0.989	0.991	0.989	0.987	0.983	0.989	0.990	0.990	0.991	0.990	0.992	0.989	0.987
		Rare	0.976	0.985	0.992	0.991	0.989	0.992	0.993	0.991	0.992	0.984	0.991	0.992	0.992	0.993	0.993	0.994	0.993	0.993
		Low	0.990	0.991	0.992	0.992	0.992	0.995	0.993	0.995	0.993	0.992	0.991	0.992	0.992	0.992	0.995	0.995	0.996	0.994
		Mixture one	0.908	0.915	0.924	0.937	0.934	0.931	0.932	0.931	0.925	0.916	0.922	0.932	0.940	0.937	0.934	0.938	0.938	0.929
		Mixture two	0.979	0.974	0.984	0.984	0.981	0.986	0.986	0.986	0.983	0.979	0.977	0.984	0.985	0.983	0.988	0.987	0.988	0.985
	20	Common	0.913	0.921	0.931	0.925	0.923	0.931	0.934	0.929	0.934	0.913	0.924	0.933	0.927	0.927	0.932	0.936	0.929	0.935
		Rare	0.921	0.941	0.960	0.959	0.964	0.964	0.968	0.962	0.952	0.941	0.953	0.967	0.970	0.975	0.970	0.972	0.967	0.968
		Low	0.945	0.948	0.959	0.962	0.962	0.958	0.964	0.960	0.954	0.951	0.954	0.961	0.963	0.963	0.961	0.965	0.963	0.960
		Mixture one	0.884	0.883	0.898	0.906	0.899	0.909	0.898	0.896	0.901	0.893	0.896	0.902	0.914	0.910	0.915	0.906	0.901	0.903
		Mixture two	0.941	0.944	0.948	0.950	0.955	0.951	0.953	0.953	0.950	0.944	0.951	0.955	0.954	0.955	0.952	0.957	0.955	0.951
	50	Common	0.843	0.867	0.874	0.883	0.878	0.880	0.877	0.876	0.877	0.846	0.877	0.877	0.887	0.884	0.886	0.885	0.877	0.881
		Rare	0.860	0.888	0.894	0.909	0.915	0.920	0.915	0.909	0.903	0.895	0.906	0.912	0.926	0.928	0.932	0.925	0.920	0.924
		Low	0.887	0.912	0.916	0.916	0.916	0.920	0.916	0.911	0.912	0.897	0.916	0.918	0.926	0.924	0.922	0.917	0.919	0.915
		Mixture one	0.833	0.853	0.859	0.866	0.870	0.870	0.877	0.866	0.863	0.846	0.859	0.866	0.875	0.876	0.876	0.880	0.873	0.867
		Mixture two	0.870	0.889	0.899	0.902	0.896	0.900	0.899	0.896	0.899	0.878	0.895	0.905	0.908	0.905	0.903	0.901	0.900	0.905
Case 2	0	Common	0.986	0.971	0.000	0.967	0.933	0.968	0.000	0.973	0.987	0.991	0.98	0.063	0.97	0.99	0.972	0.047	0.98	0.989
		Rare	0.979	0.950	0.000	0.949	0.972	0.939	0.000	0.947	0.982	0.995	0.972	0.058	0.971	0.994	0.963	0.055	0.972	0.993
		Low	0.99	0.985	0.000	0.986	0.962	0.977	0.000	0.981	0.991	0.993	0.989	0.063	0.989	0.992	0.982	0.05	0.984	0.992
		Mixture one	0.894	0.85	0.000	0.852	0.819	0.849	0.000	0.855	0.891	0.912	0.877	0.043	0.872	0.911	0.871	0.047	0.886	0.917
		Mixture two	0.977	0.952	0.000	0.959	0.922	0.961	0.000	0.961	0.975	0.981	0.967	0.051	0.968	0.981	0.967	0.046	0.968	0.979
	20	Common	0.924	0.879	0.000	0.881	0.787	0.882	0.000	0.88	0.921	0.94	0.908	0.051	0.896	0.938	0.899	0.057	0.906	0.932
		Rare	0.919	0.881	0.000	0.876	0.892	0.884	0.000	0.882	0.924	0.971	0.925	0.05	0.913	0.967	0.917	0.045	0.923	0.968
		Low	0.941	0.92	0.000	0.931	0.85	0.933	0.000	0.928	0.945	0.965	0.94	0.056	0.94	0.965	0.942	0.055	0.942	0.964
		Mixture one	0.878	0.839	0.000	0.828	0.803	0.832	0.000	0.83	0.876	0.909	0.865	0.05	0.858	0.907	0.861	0.052	0.857	0.906
		Mixture two	0.933	0.896	0.000	0.907	0.827	0.904	0.000	0.907	0.934	0.95	0.915	0.052	0.916	0.947	0.915	0.046	0.919	0.948
	50	Common	0.852	0.809	0.000	0.811	0.686	0.804	0.000	0.81	0.855	0.884	0.837	0.039	0.838	0.886	0.83	0.043	0.844	0.89
		Rare	0.838	0.796	0.000	0.793	0.794	0.799	0.000	0.791	0.852	0.921	0.857	0.053	0.838	0.928	0.852	0.059	0.842	0.928
		Low	0.88	0.874	0.000	0.873	0.771	0.888	0.000	0.859	0.889	0.929	0.897	0.042	0.891	0.926	0.907	0.048	0.895	0.93
		Mixture one	0.861	0.817	0.000	0.827	0.778	0.827	0.000	0.815	0.859	0.892	0.858	0.058	0.856	0.895	0.851	0.057	0.852	0.888
		Mixture two	0.891	0.847	0.000	0.844	0.749	0.856	0.000	0.84	0.883	0.909	0.87	0.037	0.864	0.916	0.879	0.047	0.877	0.911

Note: Common denotes gene regions only with common variants, Rare denotes gene regions only with rare variants, Low denotes gene regions only with low-frequency variants, Mixture one denotes gene regions with 20% of common variants and 80% of rare variants, and Mixture two denotes gene regions with 80% of common variants and 20% of rare variants.

##### Case Two Simulation

In this case, the time effect function is θ(t)=2+2⁡sin(πt/2) . The time effect is 0 at certain time points (*t* = 3 and *t* = 7). Therefore, the genes do not express at time points 3 and 7. The rest of the settings are the same as the case one simulation. All results of power simulation can be seen Supplementary Data S2. The setting of case two in Table 2 and Figure 2 are the same as case one simulation. Figure 2 shows that the power based on LFDAT is 0 at the time points 3 and 7 for five gene regions. It means that the associated genes cannot be detected at these time points. It further indicates that the LFDAT method can accurately detect the selective expression function of genes. Other features and trends shown by these figures are consistent with the simulation of case one. Similarly, we compare the power of the LFDAT and Smoothed FLM for case two (See Supplementary Data S1). In Table 2, the power of the LFDAT is all 0 but the Smoothed FLM has weak power at time points 3 and 7. It can be known from the results of simulation that the performance of the LFDAT is stable in different scenarios. It can detect gene switching more accurately than the Smoothed FLM. While ensuring high power, it can accurately identify whether genes are expressed.

#### 3.1.3 Estimation of ISE0, ISE1, and PMSE

We estimate the three evaluation indicators of two cases for five gene regions (See Supplementary Datas S1, S2), and we only display results of case one and case two when *c* is 3 and a sample size is 2000 in Table 3. In case one and case two simulations for the five gene regions, the means of ISE0 and ISE1 with the gene region of rare variants are the largest. Further, the means of PMSE with the gene region of low-frequency variants are largest in five gene regions. This indicates that LFDAT fits the time-varying effect function better for smaller genetic variants effects. Given the proportion of causal variants and the value of *c*, the change in the proportion of negative effects has little effect on the means and standard errors of ISE0, ISE1, and PMSE. Meanwhile, the proportion of causal variants and *c* increases (See Supplementary Datas S1, S2), and the means and the standard errors of ISE0 and PMSE gradually increase, whereas the means and standard errors of ISE1 decrease. The results of case two are smaller than that of case one, which might be affected by gene switching. The time-varying function of the genetic effect is null at a certain time point in case one so that the difference between the estimated time-varying function and the true time-varying function is smaller.

**TABLE 3:** The means and standard errors (in the parenthesis) of three indicators based on LFDA for linkage equilibrium simulation when sample size is 2000, *c* is 3

	Proportion of causal variants (%)	Proportion of negative effects (%)	ISE0					ISE1					PMSE				
			Common region	Rare region	Low-frequency region	Mixture region one	Mixture region two	Common region	Rare region	Low-frequency region	Mixture region one	Mixture region two	Common region	Rare region	Low-frequency region	Mixture region one	Mixture region two
Case 1	1	0	0.021	0.240	0.125	0.075	0.022	2.127	26.648	11.445	22.246	7.017	5.509	2.878	5.772	3.394	5.027
			(0.029)	(0.314)	(0.170)	(0.113)	(0.032)	(0.610)	(3.242)	(1.263)	(4.131)	(3.378)	(0.568)	(0.245)	(0.305)	(0.448)	(0.618)
		20	0.020	0.239	0.135	0.071	0.023	2.135	26.966	11.596	22.288	7.116	5.516	2.888	5.810	3.420	4.992
			(0.028)	(0.354)	(0.176)	(0.110)	(0.030)	(0.583)	(3.127)	(1.347)	(4.128)	(3.226)	(0.567)	(0.243)	(0.312)	(0.452)	(0.585)
		50	0.021	0.224	0.136	0.071	0.025	2.167	27.160	11.647	22.405	7.353	5.553	2.899	5.809	3.429	4.980
			(0.028)	(0.330)	(0.183)	(0.111)	(0.032)	(0.573)	(3.247)	(1.316)	(4.060)	(3.519)	(0.536)	(0.245)	(0.325)	(0.459)	(0.609)
	2	0	0.042	0.473	0.266	0.153	0.050	4.560	57.106	24.532	47.390	15.375	10.701	5.029	11.252	6.190	9.565
			(0.056)	(0.608)	(0.375)	(0.238)	(0.063)	(1.176)	(7.101)	(2.663)	(9.112)	(7.599)	(1.163)	(0.501)	(0.689)	(0.970)	(1.296)
		20	0.042	0.444	0.306	0.146	0.046	4.580	57.847	24.641	47.954	15.174	10.714	5.066	11.337	6.166	9.609
			(0.058)	(0.665)	(0.412)	(0.235)	(0.058)	(1.238)	(7.153)	(2.809)	(8.801)	(7.187)	(1.168)	(0.525)	(0.676)	(0.968)	(1.277)
		50	0.042	0.473	0.297	0.140	0.052	4.618	58.338	24.984	48.150	15.305	10.743	5.048	11.363	6.160	9.589
			(0.055)	(0.646)	(0.404)	(0.202)	(0.070)	(1.265)	(6.747)	(2.739)	(9.142)	(7.503)	(1.188)	(0.517)	(0.685)	(1.008)	(1.328)
	4	0	0.058	0.691	0.394	0.212	0.076	6.653	83.587	35.623	69.951	22.251	15.127	6.882	16.001	8.452	13.489
			(0.086)	(0.983)	(0.510)	(0.329)	(0.107)	(1.730)	(10.062)	(4.284)	(12.158)	(10.635)	(1.739)	(0.757)	(1.011)	(1.261)	(1.861)
		20	0.059	0.677	0.426	0.226	0.068	6.766	84.347	36.388	70.579	22.052	15.279	6.942	16.071	8.516	13.565
			(0.084)	(0.984)	(0.556)	(0.358)	(0.094)	(1.877)	(9.980)	(4.281)	(12.590)	(10.306)	(1.787)	(0.770)	(1.015)	(1.408)	(1.836)
		50	0.058	0.608	0.413	0.177	0.073	6.827	85.606	36.675	70.777	23.065	15.242	6.919	16.126	8.542	13.488
			(0.078)	(0.781)	(0.592)	(0.264)	(0.102)	(1.868)	(10.091)	(4.380)	(12.597)	(11.529)	(1.726)	(0.769)	(0.964)	(1.387)	(1.938)
Case 2	1	0	0.009	0.104	0.058	0.028	0.010	1.185	15.102	6.505	12.644	4.087	3.533	2.077	3.717	2.341	3.241
			(0.012)	(0.144)	(0.073)	(0.038)	(0.013)	(0.315)	(1.666)	(0.670)	(2.355)	(2.006)	(0.312)	(0.140)	(0.183)	(0.253)	(0.346)
		20	0.008	0.108	0.056	0.030	0.010	1.197	15.315	6.568	12.569	4.064	3.559	2.076	3.725	2.375	3.258
			(0.010)	(0.147)	(0.079)	(0.046)	(0.013)	(0.330)	(1.683)	(0.650)	(2.257)	(1.901)	(0.324)	(0.142)	(0.184)	(0.255)	(0.351)
		50	0.009	0.104	0.056	0.030	0.009	1.192	15.240	6.553	12.618	3.877	3.534	2.091	3.749	2.369	3.282
			(0.011)	(0.137)	(0.076)	(0.049)	(0.014)	(0.331)	(1.670)	(0.701)	(2.390)	(1.885)	(0.321)	(0.138)	(0.195)	(0.263)	(0.342)
	2	0	0.018	0.196	0.128	0.061	0.020	2.574	32.611	13.920	26.987	8.521	6.470	3.297	6.832	3.948	5.866
			(0.025)	(0.262)	(0.167)	(0.092)	(0.028)	(0.696)	(3.627)	(1.367)	(4.893)	(4.069)	(0.685)	(0.280)	(0.384)	(0.542)	(0.726)
		20	0.018	0.188	0.117	0.067	0.019	2.626	32.912	14.042	26.612	8.770	6.515	3.302	6.859	3.982	5.859
			(0.024)	(0.267)	(0.167)	(0.111)	(0.025)	(0.712)	(3.757)	(1.324)	(4.753)	(4.163)	(0.669)	(0.313)	(0.390)	(0.520)	(0.713)
		50	0.019	0.197	0.122	0.066	0.021	2.570	32.996	14.117	27.021	8.695	6.479	3.308	6.886	3.957	5.858
			(0.026)	(0.272)	(0.170)	(0.106)	(0.031)	(0.691)	(3.549)	(1.557)	(4.992)	(4.238)	(0.667)	(0.301)	(0.398)	(0.542)	(0.755)
	4	0	0.024	0.293	0.162	0.080	0.030	3.742	47.563	20.455	39.247	13.014	8.974	4.364	9.521	5.261	7.990
			(0.033)	(0.394)	(0.224)	(0.135)	(0.042)	(1.029)	(5.306)	(2.079)	(7.110)	(6.094)	(0.976)	(0.422)	(0.568)	(0.780)	(1.046)
		20	0.024	0.284	0.168	0.090	0.028	3.791	47.697	20.684	39.809	12.961	9.038	4.405	9.541	5.266	8.105
			(0.031)	(0.355)	(0.230)	(0.136)	(0.040)	(1.006)	(5.391)	(2.106)	(7.001)	(5.980)	(0.936)	(0.436)	(0.565)	(0.787)	(1.048)
		50	0.024	0.292	0.161	0.096	0.030	3.813	48.092	20.674	39.038	12.581	9.070	4.391	9.587	5.376	8.141
			(0.033)	(0.424)	(0.232)	(0.162)	(0.040)	(1.006)	(5.468)	(2.026)	(6.924)	(6.147)	(0.999)	(0.437)	(0.588)	(0.816)	(1.062)

Note: Common region denotes gene regions only with common variants, Rare region denotes gene regions only with rare variants, Low-frequency region denotes gene regions only with low-frequency variants, Mixture region one denotes gene regions with 20% of common variants and 80% of rare variants, and the Mixture region two denotes gene regions with 80% of common variants and 20% of rare variants.

### 3.2 Linkage Disequilibrium Simulation

The measure of linkage disequilibrium is *r* ^2^. It is randomly generated from a uniform distribution *U* (a,b). The measure of linkage disequilibrium between each SNP is not equal. We consider two scenarios that the *r* ^2^ is between 0.01 and 0.25, and 0.25 and 0.64. Simulation settings of type I error rates and power are the same as Section 3.1. All results of simulation can be seen Supplementary Datas S3–S6. Due to space limitations and the similar features and trends of the results of two scenarios, we only display the partial results of second scenarios (*r* ^2^ is between 0.25 and 0.64).

**FIGURE 3:**
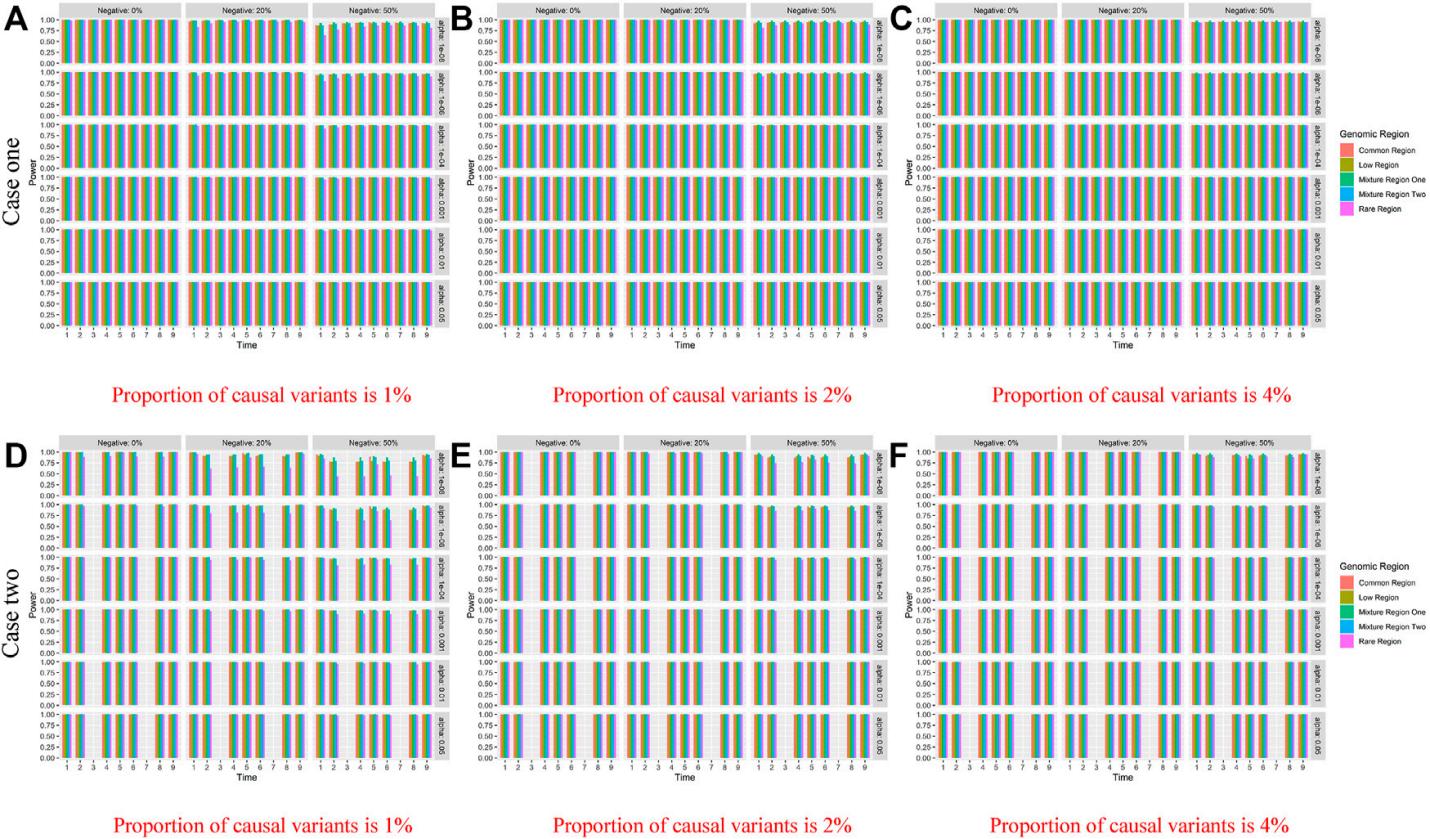
Power of linkage disequilibrium’s case one and case two based on LFDAT for the five gene regions when *c* is 3, and sample size is 2000. The **(A–C)** denotes the power results of case one. The **(D–F)** denotes the power results of case two. The time effect function is θ(t)=2+2⁡sin(πt/12) for case one, and θ(t)=2+2⁡sin(πt/2) for case two. Case one: **(A)** Proportion of causal variants is 1% **(B)** Proportion of causal variants is 2% **(C)** Proportion of causal variants is 4%. Case two: **(D)** Proportion of causal variants is 1% **(E)** Proportion of causal variants is 2% **(F)** Proportion of causal variants is 4%. Note: The *r* ^2^ measure of linkage disequilibrium is 0.25 to 0.64; Common region denotes gene regions only with common variants, Rare region denotes gene regions only with rare variants, Low region denotes gene regions only with low-frequency variants, Mixture region one denotes gene regions with 20% of common variants and 80% of rare variants, and the Mixture region two denotes gene regions with 80% of common variants and 20% of rare variants.

Table 4 shows the type I error rates of the LFDAT and Smoothed FLM at the significance level of 0.05, 0.01, and 0.001 for linkage disequilibrium simulation. The part of power results is shown in Table 5 (When significance level is 0.05, sample is 2000, *c* is 7, and proportion of causal variants is 1%) and Figure 3 (When sample is 2000, and *c* is 3). Type I error rates of rare gene region and low-frequency gene region are still lower than others. Type I error rates of the LFDAT is still lower than that of the Smoothed FLM, and the type I error rates of the Smoothed FLM is slightly inflated. It is verified once again that the use of the multiple measurement of traits can reduce the probability of making the type I errors. Power of linkage disequilibrium is very high for two cases. Especially in case one, power of five gene regions is 100% when the proportions of the negative effect of causal variants are 0%, 20%. The power of linkage disequilibrium simulation has increased a lot compared with linkage equilibrium simulation, which is due to consider the overall effect together in gene region as loci correlation.

**TABLE 4:** Type Ⅰ error rates of LFDA and Smoothed FLM based on 1,000 simulated replicates for linkage disequilibrium simulation.

α	Sample size	Gene region	LFDA									Smoothed FLM								
			t = 1	t = 2	t = 3	t = 4	t = 5	t = 6	t = 7	t = 8	t = 9	t = 1	t = 2	t = 3	t = 4	t = 5	t = 6	t = 7	t = 8	t = 9
0.05	1,000	Common	0.029	0.009	0.019	0.010	0.010	0.014	0.010	0.010	0.028	0.052	0.041	0.059	0.043	0.049	0.049	0.044	0.034	0.041
		Rare	0.010	0.005	0.005	0.008	0.005	0.004	0.005	0.008	0.010	0.041	0.054	0.051	0.051	0.056	0.052	0.056	0.047	0.048
		Low	0.018	0.007	0.008	0.002	0.009	0.005	0.009	0.006	0.021	0.034	0.048	0.047	0.044	0.045	0.044	0.061	0.055	0.042
		Mixture one	0.030	0.014	0.011	0.007	0.003	0.006	0.003	0.003	0.032	0.051	0.055	0.053	0.043	0.040	0.046	0.042	0.043	0.053
		Mixture two	0.032	0.010	0.011	0.013	0.011	0.016	0.009	0.017	0.036	0.048	0.033	0.049	0.043	0.053	0.060	0.058	0.055	0.052
	1,500	Common	0.036	0.009	0.010	0.011	0.008	0.011	0.015	0.009	0.033	0.062	0.048	0.057	0.049	0.044	0.043	0.057	0.050	0.049
		Rare	0.010	0.004	0.003	0.004	0.004	0.005	0.002	0.005	0.010	0.049	0.050	0.041	0.047	0.056	0.044	0.044	0.044	0.056
		Low	0.015	0.007	0.004	0.004	0.008	0.008	0.006	0.008	0.021	0.048	0.056	0.041	0.053	0.069	0.050	0.054	0.051	0.055
		Mixture one	0.034	0.013	0.012	0.005	0.009	0.007	0.008	0.009	0.039	0.057	0.055	0.058	0.061	0.056	0.039	0.050	0.045	0.064
		Mixture two	0.035	0.007	0.015	0.008	0.011	0.019	0.010	0.016	0.039	0.052	0.042	0.053	0.044	0.052	0.055	0.050	0.061	0.057
	2000	Common	0.032	0.020	0.016	0.011	0.018	0.015	0.020	0.019	0.043	0.046	0.054	0.041	0.045	0.057	0.052	0.048	0.060	0.054
		Rare	0.019	0.003	0.007	0.004	0.008	0.007	0.011	0.011	0.017	0.048	0.047	0.054	0.045	0.057	0.062	0.045	0.048	0.055
		Low	0.016	0.015	0.010	0.007	0.006	0.006	0.008	0.008	0.023	0.043	0.051	0.052	0.039	0.055	0.055	0.038	0.039	0.044
		Mixture one	0.023	0.006	0.007	0.007	0.012	0.003	0.011	0.007	0.026	0.053	0.047	0.043	0.055	0.065	0.059	0.064	0.045	0.048
		Mixture two	0.036	0.014	0.016	0.018	0.014	0.017	0.017	0.018	0.036	0.052	0.054	0.058	0.048	0.049	0.048	0.047	0.059	0.050
0.01	1,000	Common	0.006	0.004	0.002	0.003	0.003	0.006	0.001	0.001	0.003	0.014	0.010	0.018	0.012	0.013	0.013	0.006	0.007	0.005
		Rare	0.001	0.002	0.001	0.000	0.001	0.002	0.001	0.003	0.004	0.009	0.008	0.008	0.012	0.008	0.011	0.010	0.014	0.011
		Low	0.001	0.002	0.001	0.000	0.001	0.000	0.000	0.001	0.000	0.004	0.009	0.010	0.002	0.011	0.007	0.012	0.004	0.008
		Mixture one	0.008	0.001	0.002	0.000	0.001	0.000	0.000	0.000	0.006	0.016	0.017	0.010	0.011	0.007	0.010	0.003	0.004	0.013
		Mixture two	0.008	0.002	0.002	0.003	0.002	0.001	0.000	0.002	0.007	0.012	0.007	0.012	0.011	0.010	0.013	0.011	0.016	0.011
	1,500	Common	0.005	0.001	0.001	0.002	0.002	0.001	0.001	0.000	0.006	0.013	0.008	0.010	0.011	0.008	0.008	0.012	0.009	0.008
		Rare	0.001	0.000	0.000	0.001	0.001	0.000	0.000	0.000	0.002	0.009	0.009	0.005	0.014	0.014	0.009	0.005	0.011	0.014
		Low	0.004	0.002	0.000	0.000	0.002	0.000	0.000	0.003	0.003	0.007	0.010	0.005	0.010	0.009	0.010	0.015	0.012	0.008
		Mixture one	0.006	0.003	0.001	0.000	0.002	0.000	0.001	0.002	0.008	0.013	0.015	0.012	0.010	0.018	0.009	0.007	0.014	0.018
		Mixture two	0.006	0.001	0.002	0.002	0.003	0.003	0.003	0.001	0.007	0.010	0.005	0.014	0.008	0.014	0.013	0.007	0.009	0.009
	2000	Common	0.009	0.002	0.004	0.001	0.002	0.003	0.004	0.001	0.007	0.011	0.013	0.012	0.005	0.011	0.011	0.018	0.013	0.011
		Rare	0.003	0.000	0.000	0.000	0.001	0.000	0.001	0.001	0.004	0.013	0.004	0.010	0.007	0.012	0.012	0.017	0.010	0.013
		Low	0.005	0.002	0.000	0.000	0.001	0.001	0.001	0.000	0.004	0.009	0.015	0.009	0.010	0.009	0.011	0.006	0.010	0.010
		Mixture one	0.003	0.001	0.001	0.001	0.003	0.001	0.002	0.002	0.005	0.007	0.005	0.008	0.013	0.018	0.003	0.012	0.012	0.013
		Mixture two	0.011	0.002	0.003	0.004	0.004	0.002	0.003	0.001	0.004	0.013	0.007	0.012	0.013	0.009	0.012	0.012	0.012	0.011
0.001	1,000	Common	0.000	0.002	0.000	0.000	0.000	0.000	0.000	0.000	0.000	0.001	0.002	0.001	0.002	0.001	0.005	0.001	0.002	0.001
		Rare	0.000	0.000	0.000	0.000	0.000	0.000	0.000	0.000	0.000	0.000	0.002	0.001	0.002	0.001	0.002	0.001	0.004	0.002
		Low	0.000	0.000	0.000	0.000	0.000	0.000	0.000	0.000	0.000	0.000	0.001	0.000	0.000	0.003	0.001	0.001	0.001	0.000
		Mixture one	0.000	0.000	0.000	0.000	0.000	0.000	0.000	0.000	0.001	0.001	0.003	0.003	0.000	0.001	0.000	0.000	0.000	0.001
		Mixture two	0.001	0.000	0.000	0.000	0.000	0.001	0.000	0.000	0.001	0.001	0.000	0.000	0.001	0.001	0.001	0.000	0.001	0.001
	1,500	Common	0.000	0.000	0.000	0.000	0.001	0.001	0.000	0.000	0.000	0.000	0.001	0.000	0.002	0.001	0.001	0.001	0.000	0.001
		Rare	0.000	0.000	0.000	0.000	0.000	0.000	0.000	0.000	0.000	0.000	0.000	0.001	0.001	0.002	0.001	0.000	0.000	0.002
		Low	0.000	0.000	0.000	0.000	0.000	0.000	0.000	0.000	0.001	0.001	0.001	0.000	0.001	0.003	0.000	0.001	0.003	0.002
		Mixture one	0.001	0.000	0.000	0.000	0.000	0.000	0.000	0.000	0.002	0.001	0.001	0.001	0.000	0.002	0.001	0.001	0.001	0.002
		Mixture two	0.000	0.000	0.000	0.000	0.001	0.000	0.000	0.000	0.000	0.000	0.000	0.000	0.000	0.003	0.001	0.003	0.000	0.001
	2000	Common	0.003	0.000	0.000	0.000	0.000	0.001	0.001	0.000	0.000	0.003	0.001	0.000	0.001	0.001	0.001	0.003	0.000	0.000
		Rare	0.000	0.000	0.000	0.000	0.000	0.000	0.000	0.000	0.000	0.002	0.000	0.002	0.001	0.002	0.000	0.002	0.002	0.001
		Low	0.000	0.000	0.000	0.000	0.000	0.001	0.000	0.000	0.001	0.000	0.002	0.000	0.001	0.001	0.002	0.002	0.001	0.002
		Mixture one	0.000	0.000	0.000	0.000	0.000	0.000	0.000	0.000	0.000	0.000	0.000	0.001	0.000	0.001	0.001	0.000	0.001	0.000
		Mixture two	0.000	0.000	0.002	0.000	0.000	0.000	0.000	0.000	0.000	0.001	0.001	0.003	0.000	0.003	0.002	0.002	0.000	0.000

Note: (i) The *r* ^2^ measure of linkage disequilibrium is between 0.25 and 0.64; (ii) Common denotes gene regions only with common variants, Rare denotes gene regions only with rare variants, Low denotes gene regions only with low-frequency variants, Mixture one denotes gene regions with 20% of common variants and 80% of rare variants, and Mixture two denotes gene regions with 80% of common variants and 20% of rare variants.

**TABLE 5:** The power of linkage disequilibrium simulation based on LFDA and Smoothed FLM at significance level of 0.05 when sample size is 2000, *c* is 7 and proportion of causal variants is 1%.

	Proportion of negative effects (%)	Gene region	LFDA									Smoothed FLM								
			t = 1	t = 2	t = 3	t = 4	t = 5	t = 6	t = 7	t = 8	t = 9	t = 1	t = 2	t = 3	t = 4	t = 5	t = 6	t = 7	t = 8	t = 9
Case 1	0	Common	1.000	1.000	1.000	1.000	1.000	1.000	1.000	1.000	1.000	1.000	1.000	1.000	1.000	1.000	1.000	1.000	1.000	1.000
		Rare	1.000	1.000	1.000	1.000	1.000	1.000	1.000	1.000	1.000	1.000	1.000	1.000	1.000	1.000	1.000	1.000	1.000	1.000
		Low	1.000	1.000	1.000	1.000	1.000	1.000	1.000	1.000	1.000	1.000	1.000	1.000	1.000	1.000	1.000	1.000	1.000	1.000
		Mixture one	1.000	1.000	1.000	1.000	1.000	1.000	1.000	1.000	1.000	1.000	1.000	1.000	1.000	1.000	1.000	1.000	1.000	1.000
		Mixture two	1.000	1.000	1.000	1.000	1.000	1.000	1.000	1.000	1.000	1.000	1.000	1.000	1.000	1.000	1.000	1.000	1.000	1.000
	20	Common	1.000	1.000	1.000	1.000	1.000	1.000	1.000	1.000	1.000	1.000	1.000	1.000	1.000	1.000	1.000	1.000	1.000	1.000
		Rare	1.000	1.000	1.000	1.000	1.000	1.000	1.000	1.000	1.000	1.000	1.000	1.000	1.000	1.000	1.000	1.000	1.000	1.000
		Low	1.000	1.000	1.000	1.000	1.000	1.000	1.000	1.000	1.000	1.000	1.000	1.000	1.000	1.000	1.000	1.000	1.000	1.000
		Mixture one	1.000	1.000	1.000	1.000	1.000	1.000	1.000	1.000	1.000	1.000	1.000	1.000	1.000	1.000	1.000	1.000	1.000	1.000
		Mixture two	1.000	1.000	1.000	1.000	1.000	1.000	1.000	1.000	1.000	1.000	1.000	1.000	1.000	1.000	1.000	1.000	1.000	1.000
	50	Common	0.999	0.999	0.999	1.000	1.000	0.999	0.999	1.000	0.999	0.999	0.999	0.999	1.000	1.000	0.999	0.999	1.000	0.999
		Rare	0.997	0.999	1.000	0.999	1.000	0.999	0.999	0.999	1.000	0.997	0.999	1.000	1.000	1.000	0.999	0.999	0.999	1.000
		Low	1.000	1.000	1.000	1.000	1.000	1.000	1.000	1.000	1.000	1.000	1.000	1.000	1.000	1.000	1.000	1.000	1.000	1.000
		Mixture one	1.000	1.000	1.000	1.000	1.000	1.000	1.000	1.000	1.000	1.000	1.000	1.000	1.000	1.000	1.000	1.000	1.000	1.000
		Mixture two	0.999	0.999	1.000	0.999	0.999	0.999	0.999	0.999	0.999	0.999	0.999	1.000	0.999	0.999	0.999	0.999	0.999	0.999
Case 2	0	Common	1.000	1.000	0.000	1.000	1.000	1.000	0.000	1.000	1.000	1.000	1.000	0.052	1.000	1.000	1.000	0.060	1.000	1.000
		Rare	1.000	1.000	0.000	1.000	1.000	1.000	0.000	1.000	1.000	1.000	1.000	0.051	1.000	1.000	1.000	0.049	1.000	1.000
		Low	1.000	1.000	0.000	1.000	1.000	1.000	0.000	1.000	1.000	1.000	1.000	0.042	1.000	1.000	1.000	0.049	1.000	1.000
		Mixture one	1.000	1.000	0.000	1.000	1.000	1.000	0.000	1.000	1.000	1.000	1.000	0.058	1.000	1.000	1.000	0.043	1.000	1.000
		Mixture two	1.000	1.000	0.000	1.000	1.000	1.000	0.000	1.000	1.000	1.000	1.000	0.057	1.000	1.000	1.000	0.051	1.000	1.000
	20	Common	1.000	1.000	0.000	1.000	1.000	1.000	0.000	1.000	1.000	1.000	1.000	0.053	1.000	1.000	1.000	0.048	1.000	1.000
		Rare	1.000	1.000	0.000	1.000	1.000	1.000	0.000	1.000	1.000	1.000	1.000	0.049	1.000	1.000	1.000	0.044	1.000	1.000
		Low	1.000	1.000	0.000	1.000	1.000	1.000	0.000	1.000	1.000	1.000	1.000	0.060	1.000	1.000	1.000	0.065	1.000	1.000
		Mixture one	1.000	1.000	0.000	1.000	1.000	1.000	0.000	1.000	1.000	1.000	1.000	0.053	1.000	1.000	1.000	0.063	1.000	1.000
		Mixture two	1.000	1.000	0.000	1.000	1.000	1.000	0.000	1.000	1.000	1.000	1.000	0.053	1.000	1.000	1.000	0.041	1.000	1.000
	50	Common	1.000	1.000	0.000	1.000	1.000	1.000	0.000	1.000	1.000	1.000	1.000	0.051	1.000	1.000	1.000	0.054	1.000	1.000
		Rare	0.998	0.994	0.000	0.998	0.998	0.998	0.000	0.996	0.998	0.998	0.996	0.052	0.998	0.998	0.998	0.044	0.999	0.998
		Low	1.000	1.000	0.000	1.000	0.999	1.000	0.000	1.000	1.000	1.000	1.000	0.052	1.000	1.000	1.000	0.054	1.000	1.000
		Mixture one	1.000	0.999	0.000	1.000	0.999	0.998	0.000	0.999	1.000	1.000	0.999	0.061	1.000	1.000	0.998	0.054	1.000	1.000
		Mixture two	1.000	1.000	0.000	1.000	1.000	1.000	0.000	1.000	1.000	1.000	1.000	0.053	1.000	1.000	1.000	0.058	1.000	1.000

Note: (i) The *r* ^2^ measure of linkage disequilibrium is between 0.25 and 0.64; (ii) Common denotes gene regions only with common variants, Rare denotes gene regions only with rare variants, Low denotes gene regions only with low-frequency variants, Mixture one denotes gene regions with 20% of common variants and 80% of rare variants, and Mixture two denotes gene regions with 80% of common variants and 20% of rare variants.

At the same time, we evaluate the three indicators of two cases for five gene regions (See Supplementary Datas S3–S6), and here we only display results of case one and case two when *c* is 3 in Table 6 for second scenarios (*r* ^2^ is between 0.25 and 0.64). Same as linkage equilibrium simulation, the means of ISE0 and ISE1 of rare gene region is largest and the means of PMSE of rare gene region is smallest. This also verifies that LFDAT fits the time-varying effect function better for smaller genetic effects of gene regions.

**TABLE 6:** The means and standard errors (in the parenthesis) of three indicators based on LFDA for linkage disequilibrium simulation when sample size is 2000, *c* is 3

	Proportion of causal variants (%)	Proportion of negative effects (%)	ISE0					ISE1					PMSE				
			Common region	Rare region	Low-frequency region	Mixture region one	Mixture region two	Common region	Rare region	Low-frequency region	Mixture region one	Mixture region two	Common region	Rare region	Low-frequency region	Mixture region one	Mixture region two
Case 1	1	0	0.014	0.215	0.097	0.076	0.018	1.500	24.640	10.842	10.978	2.220	5.262	2.822	5.603	5.492	5.897
			(0.021)	(0.286)	(0.128)	(0.109)	(0.023)	(0.200)	(1.945)	(0.866)	(2.210)	(0.424)	(0.405)	(0.167)	(0.299)	(0.514)	(0.466)
		20	0.012	0.220	0.096	0.071	0.018	1.522	24.998	10.966	11.203	2.226	5.258	2.821	5.660	5.500	5.928
			(0.017)	(0.305)	(0.136)	(0.098)	(0.025)	(0.219)	(2.132)	(0.836)	(2.241)	(0.410)	(0.411)	(0.170)	(0.318)	(0.524)	(0.459)
		50	0.014	0.201	0.103	0.072	0.018	1.545	25.089	11.083	11.206	2.243	5.303	2.831	5.645	5.520	5.933
			(0.019)	(0.291)	(0.139)	(0.101)	(0.026)	(0.214)	(1.918)	(0.837)	(2.293)	(0.433)	(0.414)	(0.168)	(0.319)	(0.527)	(0.467)
	2	0	0.029	0.427	0.209	0.154	0.038	3.262	52.701	23.190	23.840	4.727	10.148	4.903	10.905	10.629	11.507
			(0.038)	(0.588)	(0.285)	(0.223)	(0.055)	(0.478)	(4.013)	(1.631)	(5.010)	(0.917)	(0.882)	(0.361)	(0.665)	(1.164)	(0.977)
		20	0.030	0.469	0.209	0.159	0.039	3.312	53.610	23.556	24.294	4.814	10.226	4.929	10.975	10.589	11.605
			(0.042)	(0.679)	(0.275)	(0.219)	(0.058)	(0.473)	(3.914)	(1.711)	(4.985)	(0.976)	(0.840)	(0.348)	(0.657)	(1.153)	(1.010)
		50	0.028	0.449	0.208	0.163	0.039	3.312	53.686	23.834	24.057	4.820	10.257	4.973	11.011	10.697	11.639
			(0.041)	(0.625)	(0.321)	(0.223)	(0.053)	(0.478)	(4.070)	(2.070)	(4.988)	(0.927)	(0.894)	(0.346)	(0.680)	(1.157)	(0.957)
	4	0	0.041	0.713	0.302	0.252	0.053	4.770	77.248	33.976	34.620	6.915	14.435	6.714	15.535	15.082	16.429
			(0.053)	(0.970)	(0.415)	(0.376)	(0.073)	(0.674)	(6.265)	(2.621)	(7.464)	(1.333)	(1.259)	(0.520)	(0.985)	(1.629)	(1.400)
		20	0.042	0.671	0.318	0.240	0.050	4.829	78.215	34.583	35.011	7.012	14.472	6.774	15.571	15.114	16.459
			(0.058)	(0.941)	(0.426)	(0.331)	(0.070)	(0.701)	(6.598)	(3.307)	(7.349)	(1.386)	(1.250)	(0.526)	(0.949)	(1.763)	(1.368)
		50	0.042	0.637	0.310	0.241	0.052	4.817	78.831	34.598	35.228	7.045	14.468	6.787	15.662	15.237	16.546
			(0.059)	(0.932)	(0.428)	(0.354)	(0.071)	(0.692)	(6.105)	(2.502)	(7.163)	(1.380)	(1.282)	(0.525)	(0.989)	(1.659)	(1.381)
Case 2	1	0	0.006	0.091	0.041	0.029	0.007	0.878	13.948	6.194	6.516	1.312	3.476	2.069	3.661	3.548	3.855
			(0.008)	(0.129)	(0.056)	(0.039)	(0.009)	(0.107)	(0.906)	(0.392)	(1.372)	(0.264)	(0.221)	(0.100)	(0.187)	(0.330)	(0.259)
		20	0.006	0.092	0.040	0.027	0.007	0.880	14.068	6.257	6.589	1.320	3.480	2.066	3.662	3.523	3.870
			(0.008)	(0.116)	(0.057)	(0.039)	(0.010)	(0.109)	(1.015)	(0.367)	(1.412)	(0.248)	(0.226)	(0.101)	(0.188)	(0.318)	(0.247)
		50	0.006	0.089	0.041	0.027	0.007	0.884	14.164	6.311	6.522	1.351	3.471	2.066	3.666	3.538	3.878
			(0.009)	(0.120)	(0.053)	(0.037)	(0.010)	(0.114)	(0.923)	(0.398)	(1.332)	(0.310)	(0.226)	(0.102)	(0.176)	(0.305)	(0.262)
	2	0	0.013	0.190	0.087	0.053	0.016	1.864	29.977	13.332	13.878	2.856	6.275	3.292	6.697	6.461	7.146
			(0.019)	(0.246)	(0.110)	(0.074)	(0.022)	(0.233)	(1.974)	(0.810)	(2.794)	(0.626)	(0.467)	(0.203)	(0.370)	(0.658)	(0.561)
		20	0.013	0.194	0.088	0.053	0.016	1.892	30.220	13.441	13.989	2.892	6.319	3.292	6.688	6.461	7.146
			(0.018)	(0.269)	(0.121)	(0.074)	(0.022)	(0.244)	(1.954)	(0.747)	(2.993)	(0.688)	(0.471)	(0.208)	(0.393)	(0.702)	(0.570)
		50	0.013	0.204	0.087	0.059	0.017	1.895	30.472	13.560	14.027	2.873	6.320	3.288	6.696	6.446	7.179
			(0.018)	(0.298)	(0.114)	(0.082)	(0.023)	(0.244)	(2.134)	(0.865)	(2.908)	(0.609)	(0.480)	(0.209)	(0.386)	(0.670)	(0.535)
	4	0	0.019	0.261	0.125	0.081	0.022	2.727	43.856	19.508	20.433	4.123	8.745	4.354	9.340	8.970	9.984
			(0.029)	(0.347)	(0.169)	(0.118)	(0.030)	(0.343)	(2.944)	(1.198)	(4.177)	(0.816)	(0.703)	(0.293)	(0.540)	(0.949)	(0.804)
		20	0.019	0.260	0.119	0.089	0.021	2.759	44.145	19.702	20.322	4.214	8.754	4.347	9.334	9.022	10.045
			(0.025)	(0.346)	(0.155)	(0.129)	(0.027)	(0.350)	(2.815)	(1.187)	(4.260)	(0.853)	(0.698)	(0.296)	(0.565)	(0.984)	(0.796)
		50	0.018	0.289	0.127	0.077	0.022	2.775	44.346	19.757	20.320	4.224	8.802	4.355	9.374	9.003	10.032
			(0.024)	(0.442)	(0.185)	(0.105)	(0.030)	(0.344)	(2.867)	(1.210)	(4.165)	(0.973)	(0.716)	(0.299)	(0.543)	(0.975)	(0.791)

Note: (i) The *r* ^2^ measure of linkage disequilibrium is between 0.25 and 0.64; (ii) Common region denotes gene regions only with common variants, Rare region denotes gene regions only with rare variants, Low-frequency region denotes gene regions only with low-frequency variants, Mixture region one denotes gene regions with 20% of common variants and 80% of rare variants, and the Mixture region two denotes gene regions with 80% of common variants and 20% of rare variants.

### 3.3 Comparison of Simulation

Linkage equilibrium simulation and the two scenarios of linkage disequilibrium simulation are compared and analyzed when sample size is 1,500, constant *c* is 5, and the proportion of casual variants is 2% (Tables 7–9). The characteristics of the remaining simulation results are similar to that of above simulation. In general, the type I error rates of the two scenarios of linkage disequilibrium simulation is larger than that of linkage equilibrium simulation. This is because the increase in power will increase the type I error rates. From results of two cases we can know that because the *r* ^2^ measure of linkage disequilibrium increase, the power also increases for five gene regions. The power of linkage disequilibrium simulation is significantly less affected by the proportion of negative effects than that of linkage equilibrium simulation, which is also due to the interaction between genes.

**TABLE 7:** Compare the type Ⅰ error rates of linkage equilibrium and linkage disequilibrium simulation based on LFDAT when sample size is 1,500.

α	Simulation	Gene region	LFDA								
			t = 1	t = 2	t = 3	t = 4	t = 5	t = 6	t = 7	t = 8	t = 9
0.05	LE	Common	0.025	0.009	0.002	0.010	0.005	0.009	0.007	0.009	0.027
		Rare	0.012	0.004	0.000	0.002	0.000	0.003	0.005	0.002	0.007
		Low	0.019	0.006	0.011	0.008	0.002	0.006	0.004	0.013	0.017
		Mixture one	0.013	0.010	0.006	0.002	0.000	0.008	0.002	0.006	0.021
		Mixture two	0.031	0.014	0.009	0.012	0.005	0.007	0.010	0.010	0.028
	LD1	Common	0.029	0.012	0.012	0.009	0.012	0.010	0.008	0.017	0.031
		Rare	0.007	0.005	0.004	0.005	0.003	0.003	0.003	0.006	0.011
		Low	0.020	0.007	0.007	0.006	0.007	0.006	0.004	0.006	0.022
		Mixture one	0.019	0.005	0.006	0.003	0.006	0.012	0.005	0.011	0.021
		Mixture two	0.030	0.011	0.008	0.008	0.006	0.008	0.006	0.014	0.028
	LD2	Common	0.036	0.009	0.010	0.011	0.008	0.011	0.015	0.009	0.033
		Rare	0.010	0.004	0.003	0.004	0.004	0.005	0.002	0.005	0.010
		Low	0.015	0.007	0.004	0.004	0.008	0.008	0.006	0.008	0.021
		Mixture one	0.034	0.013	0.012	0.005	0.009	0.007	0.008	0.009	0.039
		Mixture two	0.035	0.007	0.015	0.008	0.011	0.019	0.010	0.016	0.039
0.01	LE	Common	0.005	0.002	0.000	0.001	0.001	0.000	0.000	0.002	0.003
		Rare	0.002	0.000	0.000	0.000	0.000	0.000	0.001	0.000	0.000
		Low	0.003	0.000	0.001	0.000	0.000	0.002	0.000	0.001	0.003
		Mixture one	0.002	0.003	0.001	0.001	0.000	0.000	0.000	0.001	0.001
		Mixture two	0.006	0.001	0.001	0.001	0.000	0.002	0.002	0.002	0.004
	LD1	Common	0.003	0.002	0.001	0.002	0.000	0.001	0.001	0.005	0.005
		Rare	0.001	0.001	0.000	0.000	0.001	0.000	0.001	0.001	0.002
		Low	0.003	0.002	0.000	0.001	0.002	0.001	0.000	0.000	0.002
		Mixture one	0.005	0.000	0.002	0.000	0.002	0.000	0.001	0.001	0.001
		Mixture two	0.007	0.001	0.000	0.002	0.001	0.001	0.000	0.004	0.002
	LD2	Common	0.005	0.001	0.001	0.002	0.002	0.001	0.001	0.000	0.006
		Rare	0.001	0.000	0.000	0.001	0.001	0.000	0.000	0.000	0.002
		Low	0.004	0.002	0.000	0.000	0.002	0.000	0.000	0.003	0.003
		Mixture one	0.006	0.003	0.001	0.000	0.002	0.000	0.001	0.002	0.008
		Mixture two	0.006	0.001	0.002	0.002	0.003	0.003	0.003	0.001	0.007
0.001	LE	Common	0.001	0.000	0.000	0.000	0.000	0.000	0.000	0.000	0.000
		Rare	0.000	0.000	0.000	0.000	0.000	0.000	0.000	0.000	0.000
		Low	0.000	0.000	0.000	0.000	0.000	0.000	0.000	0.000	0.000
		Mixture one	0.000	0.000	0.001	0.000	0.000	0.000	0.000	0.000	0.000
		Mixture two	0.000	0.000	0.000	0.000	0.000	0.001	0.000	0.000	0.000
	LD1	Common	0.000	0.000	0.000	0.000	0.000	0.000	0.000	0.000	0.000
		Rare	0.000	0.000	0.000	0.000	0.000	0.000	0.000	0.000	0.000
		Low	0.000	0.000	0.000	0.000	0.000	0.000	0.000	0.000	0.001
		Mixture one	0.001	0.000	0.000	0.000	0.000	0.000	0.000	0.000	0.000
		Mixture two	0.000	0.000	0.000	0.000	0.000	0.000	0.000	0.000	0.000
	LD2	Common	0.000	0.000	0.000	0.000	0.001	0.001	0.000	0.000	0.000
		Rare	0.000	0.000	0.000	0.000	0.000	0.000	0.000	0.000	0.000
		Low	0.000	0.000	0.000	0.000	0.000	0.000	0.000	0.000	0.001
		Mixture one	0.001	0.000	0.000	0.000	0.000	0.000	0.000	0.000	0.002
		Mixture two	0.000	0.000	0.000	0.000	0.001	0.000	0.000	0.000	0.000

Note: Common denotes gene regions only with common variants, Rare denotes gene regions only with rare variants, Low denotes gene regions only with low-frequency variants, Mixture one denotes gene regions with 20% of common variants and 80% of rare variants, and Mixture two denotes gene regions with 80% of common variants and 20% of rare variants. LE, denotes linkage equilibrium simulation, LD1 denotes linkage disequilibrium simulation when *r* ^*2*^ is between 0.01 and 0.25, LD2 denotes linkage disequilibrium simulation when *r* ^*2*^ is between 0.25 and 0.64.

**TABLE 8:** Compare the power of linkage equilibrium and linkage disequilibrium simulation based on LFDAT when significant level is 0.05, sample size is 1,500, *c* is 5, and proportion of casual variants is 2%.

Gene region	Simulation	Proportion of negative effects (%)	Case 1									Case 2								
			t = 1	t = 2	t = 3	t = 4	t = 5	t = 6	t = 7	t = 8	t = 9	t = 1	t = 2	t = 3	t = 4	t = 5	t = 6	t = 7	t = 8	t = 9
Common	LE	0	0.991	0.990	0.988	0.990	0.987	0.988	0.990	0.991	0.989	0.988	0.976	0.000	0.979	0.951	0.982	0.000	0.981	0.993
		20	0.917	0.922	0.929	0.927	0.924	0.932	0.932	0.937	0.927	0.917	0.886	0.000	0.894	0.733	0.897	0.000	0.886	0.919
		50	0.735	0.734	0.744	0.752	0.752	0.757	0.760	0.755	0.759	0.708	0.676	0.000	0.684	0.482	0.675	0.000	0.684	0.700
	LD1	0	1.000	1.000	1.000	1.000	1.000	1.000	1.000	1.000	1.000	1.000	1.000	0.000	1.000	1.000	1.000	0.000	1.000	1.000
		20	0.996	0.996	0.996	0.997	0.996	0.997	0.997	0.998	0.998	0.994	0.992	0.000	0.991	0.986	0.992	0.000	0.988	0.992
		50	0.938	0.949	0.952	0.954	0.957	0.950	0.949	0.951	0.947	0.942	0.916	0.000	0.913	0.851	0.923	0.000	0.915	0.942
	LD2	0	1.000	1.000	1.000	1.000	1.000	1.000	1.000	1.000	1.000	1.000	1.000	0.000	1.000	1.000	1.000	0.000	1.000	1.000
		20	1.000	1.000	1.000	1.000	1.000	1.000	1.000	1.000	1.000	1.000	1.000	0.000	1.000	1.000	1.000	0.000	1.000	1.000
		50	1.000	1.000	1.000	1.000	1.000	1.000	1.000	1.000	1.000	1.000	0.999	0.000	0.999	0.998	1.000	0.000	1.000	1.000
Rare	LE	0	0.992	0.996	0.998	0.998	0.999	0.998	0.998	1.000	1.000	0.989	0.976	0.000	0.980	0.983	0.981	0.000	0.981	0.988
		20	0.913	0.944	0.944	0.949	0.954	0.958	0.952	0.954	0.951	0.895	0.881	0.000	0.874	0.861	0.880	0.000	0.873	0.896
		50	0.786	0.837	0.854	0.861	0.864	0.859	0.862	0.859	0.848	0.721	0.709	0.000	0.713	0.657	0.716	0.000	0.713	0.722
	LD1	0	1.000	1.000	1.000	1.000	1.000	1.000	1.000	1.000	1.000	1.000	1.000	0.000	1.000	1.000	1.000	0.000	1.000	1.000
		20	0.993	0.996	0.997	0.995	0.996	0.998	0.996	0.996	0.996	0.996	0.987	0.000	0.989	0.994	0.988	0.000	0.987	0.997
		50	0.946	0.952	0.954	0.962	0.959	0.961	0.955	0.957	0.945	0.914	0.903	0.000	0.905	0.876	0.898	0.000	0.908	0.911
	LD2	0	1.000	1.000	1.000	1.000	1.000	1.000	1.000	1.000	1.000	1.000	1.000	0.000	1.000	1.000	1.000	0.000	1.000	1.000
		20	1.000	1.000	1.000	1.000	1.000	1.000	1.000	1.000	1.000	1.000	1.000	0.000	1.000	1.000	1.000	0.000	1.000	1.000
		50	1.000	1.000	1.000	1.000	1.000	1.000	1.000	0.999	1.000	0.999	0.995	0.000	0.998	0.996	0.997	0.000	0.994	0.999
Low	LE	0	1.000	1.000	1.000	1.000	1.000	1.000	1.000	1.000	1.000	1.000	0.996	0.000	0.997	0.983	0.997	0.000	0.996	0.998
		20	0.948	0.948	0.955	0.954	0.962	0.962	0.955	0.955	0.953	0.895	0.887	0.000	0.903	0.798	0.907	0.000	0.893	0.895
		50	0.827	0.837	0.837	0.846	0.840	0.855	0.849	0.850	0.839	0.752	0.761	0.000	0.781	0.587	0.785	0.000	0.776	0.749
	LD1	0	1.000	1.000	1.000	1.000	1.000	1.000	1.000	1.000	1.000	1.000	1.000	0.000	1.000	0.999	1.000	0.000	1.000	1.000
		20	0.996	0.996	0.996	0.998	0.997	0.998	0.998	0.996	0.997	0.998	0.997	0.000	0.998	0.989	0.999	0.000	0.996	0.999
		50	0.955	0.956	0.964	0.965	0.965	0.963	0.962	0.958	0.958	0.942	0.938	0.000	0.938	0.860	0.941	0.000	0.934	0.940
	LD2	0	1.000	1.000	1.000	1.000	1.000	1.000	1.000	1.000	1.000	1.000	1.000	0.000	1.000	1.000	1.000	0.000	1.000	1.000
		20	1.000	1.000	1.000	1.000	1.000	1.000	1.000	1.000	1.000	1.000	1.000	0.000	1.000	1.000	1.000	0.000	1.000	1.000
		50	0.998	0.998	0.999	0.999	0.998	0.999	0.998	0.999	0.998	0.999	0.998	0.000	0.999	0.996	0.999	0.000	0.998	0.999
Mixture One	LE	0	0.961	0.969	0.972	0.968	0.974	0.975	0.972	0.975	0.971	0.958	0.928	0.000	0.938	0.905	0.939	0.000	0.930	0.958
		20	0.913	0.917	0.930	0.931	0.927	0.925	0.933	0.930	0.919	0.915	0.876	0.000	0.896	0.821	0.887	0.000	0.868	0.903
		50	0.846	0.874	0.878	0.890	0.894	0.897	0.901	0.890	0.881	0.840	0.807	0.000	0.814	0.714	0.815	0.000	0.800	0.847
	LD1	0	1.000	1.000	1.000	1.000	1.000	1.000	1.000	1.000	1.000	1.000	1.000	0.000	1.000	1.000	1.000	0.000	0.999	1.000
		20	0.991	0.995	0.995	0.994	0.992	0.993	0.994	0.992	0.993	0.997	0.991	0.000	0.994	0.984	0.995	0.000	0.989	0.996
		50	0.980	0.984	0.985	0.990	0.987	0.990	0.988	0.988	0.985	0.980	0.966	0.000	0.966	0.925	0.969	0.000	0.961	0.978
	LD2	0	1.000	1.000	1.000	1.000	1.000	1.000	1.000	1.000	1.000	1.000	1.000	0.000	1.000	1.000	1.000	0.000	1.000	1.000
		20	1.000	1.000	1.000	1.000	1.000	1.000	1.000	1.000	1.000	1.000	1.000	0.000	1.000	1.000	1.000	0.000	1.000	1.000
		50	1.000	0.999	1.000	1.000	1.000	0.999	1.000	1.000	1.000	1.000	0.999	0.000	0.999	0.999	0.999	0.000	1.000	0.999
Mixture Two	LE	0	0.992	0.993	0.990	0.991	0.992	0.995	0.994	0.995	0.995	0.991	0.979	0.000	0.985	0.951	0.985	0.000	0.983	0.991
		20	0.915	0.923	0.925	0.936	0.937	0.940	0.931	0.933	0.929	0.909	0.875	0.000	0.893	0.761	0.883	0.000	0.874	0.899
		50	0.789	0.795	0.806	0.808	0.813	0.815	0.813	0.813	0.808	0.768	0.743	0.000	0.734	0.538	0.741	0.000	0.721	0.777
	LD1	0	1.000	1.000	1.000	1.000	1.000	1.000	1.000	1.000	1.000	1.000	1.000	0.000	1.000	1.000	1.000	0.000	1.000	1.000
		20	0.992	0.992	0.994	0.992	0.993	0.994	0.992	0.994	0.993	0.997	0.996	0.000	0.994	0.985	0.995	0.000	0.997	0.997
		50	0.932	0.941	0.942	0.950	0.948	0.944	0.944	0.944	0.940	0.942	0.928	0.000	0.943	0.854	0.931	0.000	0.932	0.941
	LD2	0	1.000	1.000	1.000	1.000	1.000	1.000	1.000	1.000	1.000	1.000	1.000	0.000	1.000	1.000	1.000	0.000	1.000	1.000
		20	1.000	1.000	1.000	1.000	1.000	1.000	1.000	1.000	1.000	1.000	1.000	0.000	1.000	1.000	1.000	0.000	1.000	1.000
		50	1.000	0.999	1.000	1.000	1.000	1.000	1.000	1.000	1.000	0.997	0.998	0.000	0.998	0.997	0.997	0.000	0.996	0.997

Note: Common denotes gene regions only with common variants, Rare denotes gene regions only with rare variants, Low denotes gene regions only with low-frequency variants, Mixture one denotes gene regions with 20% of common variants and 80% of rare variants, and Mixture two denotes gene regions with 80% of common variants and 20% of rare variants. LE, denotes linkage equilibrium simulation, LD1 denotes linkage disequilibrium simulation when *r* ^*2*^ is between 0.01 and 0.25, LD2 denotes linkage disequilibrium simulation when *r* ^*2*^ is between 0.25 and 0.64.

**TABLE 9:** Compare the estimated means and standard errors (in the parenthesis) of three indicators for linkage equilibrium and linkage disequilibrium simulation based on LFDA method when sample size is 1,500, *c* is 5, and proportion of casual variants is 2%.

	Simulation	Proportion of negative effects (%)	ISE0					ISE1					PMSE				
			Common region	Rare region	Low-frequency region	Mixture region one	Mixture region two	Common region	Rare region	Low-frequency region	Mixture region one	Mixture region two	Common region	Rare region	Low-frequency region	Mixture region one	Mixture region two
Case 1	LE	0	0.094	0.940	0.652	0.301	0.095	4.263	53.520	23.161	44.892	14.530	20.067	8.967	21.179	11.246	17.846
			(0.132)	(1.256)	(0.897)	(0.437)	(0.123)	(0.878)	(5.466)	(2.597)	(6.130)	(4.935)	(1.694)	(0.758)	(1.117)	(1.399)	(1.858)
		20	0.095	0.882	0.594	0.292	0.102	4.344	55.046	23.742	45.444	14.881	20.240	9.086	21.481	11.305	17.961
			(0.132)	(1.211)	(0.792)	(0.430)	(0.147)	(0.907)	(5.469)	(2.519)	(5.994)	(5.050)	(1.890)	(0.771)	(1.152)	(1.392)	(1.882)
		50	0.093	0.893	0.658	0.305	0.102	4.382	55.385	24.188	45.453	14.475	20.398	9.159	21.587	11.319	18.199
			(0.128)	(1.177)	(0.937)	(0.466)	(0.134)	(0.872)	(5.589)	(2.774)	(5.847)	(4.688)	(1.738)	(0.814)	(1.180)	(1.339)	(1.893)
	LD1	0	0.072	0.867	0.502	0.345	0.104	3.570	50.547	22.150	31.986	6.287	20.154	9.112	21.366	16.162	21.750
			(0.100)	(1.089)	(0.709)	(0.493)	(0.144)	(0.607)	(3.914)	(2.014)	(4.871)	(1.444)	(1.617)	(0.647)	(1.021)	(1.702)	(1.639)
		20	0.068	0.867	0.496	0.342	0.100	3.625	51.869	22.681	32.242	6.434	20.348	9.179	21.705	16.390	22.008
			(0.092)	(1.211)	(0.686)	(0.470)	(0.138)	(0.583)	(4.084)	(2.083)	(4.817)	(1.489)	(1.562)	(0.666)	(1.063)	(1.758)	(1.633)
		50	0.071	0.817	0.501	0.348	0.102	3.679	52.621	23.049	32.364	6.558	20.545	9.232	21.754	16.488	22.139
			(0.096)	(1.118)	(0.703)	(0.473)	(0.139)	(0.584)	(3.878)	(2.112)	(4.895)	(1.580)	(1.549)	(0.640)	(1.148)	(1.804)	(1.594)
	LD2	0	0.061	0.871	0.444	0.270	0.080	3.072	49.213	21.558	22.395	4.428	19.331	8.883	20.777	20.075	21.867
			(0.081)	(1.120)	(0.580)	(0.371)	(0.106)	(0.354)	(3.871)	(1.747)	(3.489)	(0.603)	(1.483)	(0.688)	(1.386)	(1.890)	(1.755)
		20	0.062	0.853	0.474	0.304	0.078	3.111	50.440	22.191	22.617	4.489	19.424	8.973	21.022	20.434	21.967
			(0.084)	(1.143)	(0.638)	(0.440)	(0.107)	(0.337)	(4.512)	(1.834)	(3.493)	(0.601)	(1.430)	(0.662)	(1.339)	(1.987)	(1.785)
		50	0.059	0.864	0.432	0.310	0.078	3.173	51.085	22.448	22.797	4.601	19.634	9.008	21.251	20.501	22.222
			(0.077)	(1.199)	(0.579)	(0.420)	(0.110)	(0.356)	(3.620)	(1.779)	(3.516)	(0.655)	(1.454)	(0.671)	(1.319)	(2.002)	(1.772)
Case 2	LE	0	0.035	0.357	0.244	0.128	0.046	2.429	30.721	13.170	25.563	8.196	11.782	5.561	12.572	6.809	10.601
			(0.048)	(0.480)	(0.345)	(0.202)	(0.060)	(0.479)	(2.844)	(1.155)	(3.300)	(2.727)	(1.009)	(0.454)	(0.644)	(0.767)	(1.104)
		20	0.039	0.358	0.241	0.121	0.043	2.479	31.295	13.446	25.666	8.430	11.987	5.578	12.666	6.861	10.603
			(0.050)	(0.480)	(0.322)	(0.171)	(0.055)	(0.488)	(2.569)	(1.227)	(3.197)	(2.843)	(1.005)	(0.431)	(0.651)	(0.788)	(1.046)
		50	0.036	0.355	0.245	0.119	0.041	2.507	31.339	13.552	25.849	8.106	12.044	5.630	12.715	6.867	10.740
			(0.048)	(0.496)	(0.361)	(0.182)	(0.058)	(0.475)	(2.575)	(1.123)	(3.401)	(2.628)	(0.991)	(0.459)	(0.647)	(0.863)	(1.066)
	LD1	0	0.028	0.366	0.199	0.118	0.037	2.026	28.752	12.660	17.391	3.731	11.978	5.712	12.698	9.943	12.798
			(0.037)	(0.513)	(0.268)	(0.163)	(0.053)	(0.299)	(1.915)	(0.947)	(2.573)	(0.887)	(0.900)	(0.363)	(0.570)	(0.958)	(0.878)
		20	0.028	0.351	0.203	0.135	0.036	2.068	29.114	12.845	17.498	3.759	12.078	5.761	12.801	10.026	12.851
			(0.038)	(0.467)	(0.284)	(0.181)	(0.045)	(0.320)	(1.858)	(0.811)	(2.616)	(0.916)	(0.902)	(0.383)	(0.618)	(1.017)	(0.925)
		50	0.027	0.376	0.204	0.116	0.037	2.074	29.618	13.026	17.808	3.781	12.143	5.741	12.865	9.947	12.952
			(0.037)	(0.510)	(0.284)	(0.153)	(0.048)	(0.297)	(2.271)	(0.974)	(2.595)	(0.900)	(0.880)	(0.368)	(0.596)	(1.029)	(0.889)
	LD2	0	0.024	0.344	0.174	0.117	0.030	1.771	28.726	12.259	12.307	2.711	11.549	5.487	12.611	12.333	13.343
			(0.032)	(0.450)	(0.231)	(0.155)	(0.041)	(0.185)	(1.572)	(0.795)	(1.803)	(0.349)	(0.852)	(0.375)	(0.768)	(1.101)	(0.999)
		20	0.023	0.323	0.160	0.123	0.034	1.810	29.327	12.513	12.495	2.737	11.626	5.456	12.575	12.266	13.416
			(0.031)	(0.416)	(0.210)	(0.191)	(0.046)	(0.186)	(1.668)	(0.823)	(1.804)	(0.369)	(0.851)	(0.351)	(0.733)	(1.083)	(1.001)
		50	0.023	0.381	0.176	0.119	0.031	1.840	29.771	12.644	12.547	2.780	11.702	5.444	12.575	12.276	13.494
			(0.031)	(0.537)	(0.255)	(0.161)	(0.042)	(0.184)	(1.907)	(0.723)	(1.756)	(0.359)	(0.848)	(0.395)	(0.775)	(1.079)	(0.950)

Note: Common denotes gene regions only with common variants, Rare denotes gene regions only with rare variants, Low denotes gene regions only with low-frequency variants, Mixture one denotes gene regions with 20% of common variants and 80% of rare variants, and Mixture two denotes gene regions with 80% of common variants and 20% of rare variants. LE, denotes linkage equilibrium simulation, LD1 denotes linkage disequilibrium simulation when *r* ^*2*^ is between 0.01 and 0.25, LD2 denotes linkage disequilibrium simulation when *r* ^*2*^ is between 0.25 and 0.64.

In two cases, as the *r* ^2^ measure of linkage disequilibrium increase, the means and standard errors of ISE0 of common gene region, low-frequency gene region, and mixture gene region two gradually decrease, and the standard errors of ISE1 of the five gene regions decrease. The means of PMSE of common gene region, rare gene region, and low-frequency gene region first increase and then decrease, but the change is not large. These phenomena may be caused by the fact that the fitting errors of the LFDAT to the time-varying effect function gradually decreases as the *r* ^2^ measure of linkage disequilibrium increases. Although LFDAT has a little bias for fitting of the time-varying effect function, it does not affect its detection efficiency on a gene region.

In general, LFDAT performs well for both linkage equilibrium and linkage disequilibrium simulations, and has a lower type I error rates with a higher power for gene regions.

## 4 Application to PSA Data Set

We apply LFDAT to a longitudinal data set (Campbell et al., 2018) of an Oryza sativa projected shoot area (PSA) to demonstrate the applicability of LFDAT. That data set selected 378 lines of RDP1 (Zhao et al., 2011). All experiments were carried on at the Plant Accelerator in the Australian Plant Phenomics Facility at the University of Adelaide, SA, Australia. The experiments were repeated three times from February to April 2016. For details of the experimental design, see Campbell et al. (2018). Briefly, we first transplanted three uniformly germinated seedlings into pots. Seven days after the transplant (DAT), the plants were thinned to one seedling per pot. The plants were imaged daily from 13 to 33 DAT using a red-green-blue camera from two side-view angles, separated by 90° and a single top view. Each experiment adopted a partially replicated design with 54 lines selected randomly, and they were repeated twice. Three experiments produced 73,537 images, and “Plant pixels” were extracted from RGB images using the LemnaGrid software. The sum of the “plant pixels” extracted from the three RGB images is used as an indicator to measure shoot biomass. This indicator is referred to as PSA. PSA has been proved to be an accurate expression of shoot biomass (Golzarian et al., 2011; Campbell et al., 2015; Knecht et al., 2016), which can describe the morphology and dynamic growth of plants.

**FIGURE 4:**
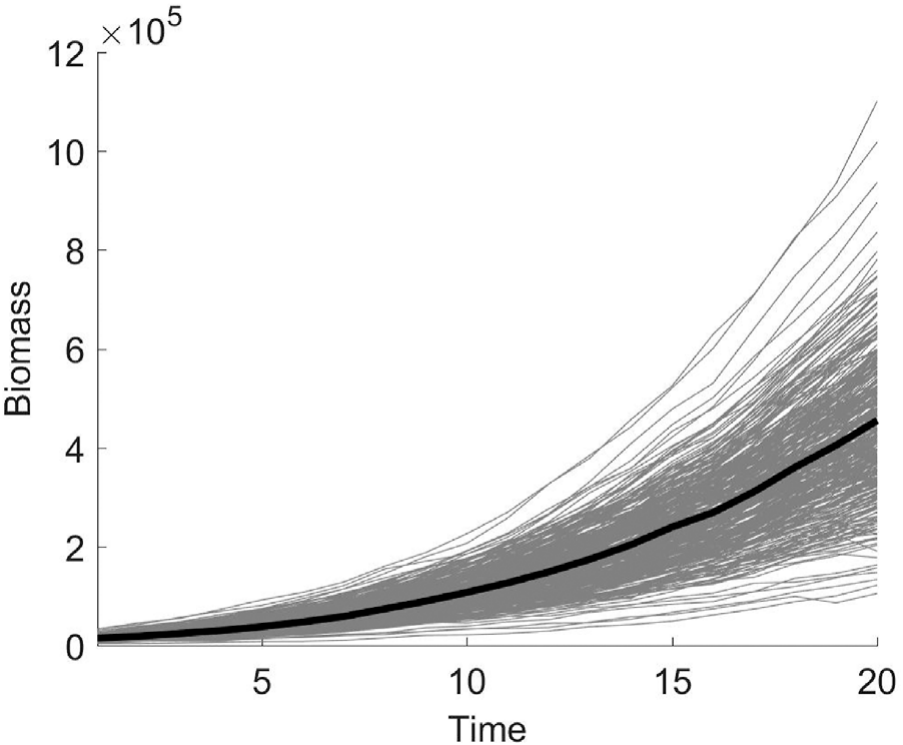
The shoot biomass development trajectory of 350 samples. The solid gray line represents the trajectory curve of 350 samples, and the solid black line represents the average trajectory curve.

The first set of data from the first repeated experiment is selected as the phenotypic data, and samples with missing values are eliminated. The 350 samples remaining are used for the subsequent analysis. The development trajectories of the shoot biomass are shown in Figure 4, with the shoot biomass trajectories for all individuals indicated in the background. The genotype data contains a total of 36,901 markers on 12 chromosomes. The missing genotype is estimated, and SNPs with a minimum allele frequency of less than 0.005 are deleted. Finally, 36,058 SNPs remained. In order to be consistent with the results of Campbell et al. (2015), we treat each chromosome as a gene region for the association analysis. The number of SNPs and the *p*-value of the association analysis of each gene region are shown in Table 10. The correlation coefficients between the measured traits at each time point are close to one. Significant SNP sites have been identified on each chromosome. Further, the SNP sites of chromosome 3 are more significant. In the type I error rates simulation, it can be seen from Table 1 that the type I error rates is low, which indicates that the LFDAT method is less likely to identify false gene region. Therefore, the detection of the significant SNPs on each chromosome is basically credible in our study, which is consistent with the results of Campbell et al. (2015). However, significant SNP sites were not detected at the first two time points. It may be that the PSA growth trajectory is exponentially increasing, and the value is too large, leading to the variation range of PSA being too small at the first and second time points. Then, the difference between the rice populations cannot be identified.

**TABLE 10:** The number of SNPs and the *p*-value of the association analysis of each gene region based on LFDAT at significance level of 0.05

Chr	No. Of SNPs in test	*p*-value									
		t = 1	t = 2	t = 3	t = 4	t = 5	t = 6	t = 7	t = 8	t = 9	t = 10
1	6,332	1	1	2.0 × 10^−2^	3.2 × 10^−6^	6.6 × 10^−8^	3.0 × 10^−8^	8.3 × 10^−9^	3.2 × 10^−10^	2.3 × 10^−11^	1.7 × 10^−12^
2	3,808	1	1	6.7 × 10^−1^	1.5 × 10^−5^	7.2 × 10^−7^	1.1 × 10^−6^	2.4 × 10^−7^	6.8 × 10^−8^	3.3 × 10^−9^	1.2 × 10^−9^
3	4,298	1	1	1.9 × 10^−2^	1.2 × 10^−8^	3.6 × 10^−10^	2.2 × 10^−10^	5.9 × 10^−11^	3.0 × 10^−12^	7.0 × 10^−14^	5.7 × 10^−14^
4	2,802	1	1	9.8 × 10^−3^	1.4 × 10^−6^	7.0 × 10^−8^	5.2 × 10^−8^	6.6 × 10^−9^	3.9 × 10^−9^	2.4 × 10^−10^	1.2 × 10^−10^
5	2,800	1	1	1.2 × 10^−2^	5.1 × 10^−7^	1.1 × 10^−8^	9.3 × 10^−9^	7.5 × 10^−10^	7.0 × 10^−11^	4.6 × 10^−12^	4.8 × 10^−12^
6	3,177	1	1	9.1 × 10^−4^	2.7 × 10^−8^	2.5 × 10^−10^	1.2 × 10^−10^	2.2 × 10^−11^	3.1 × 10^−12^	4.8 × 10^−14^	1.8 × 10^−13^
7	2024	1	1	1.2 × 10^−5^	1.0 × 10^−9^	4.5 × 10^−11^	7.0 × 10^−11^	3.4 × 10^−12^	1.8 × 10^−12^	3.1 × 10^−13^	2.8 × 10^−13^
8	2,233	1	1	5.8 × 10^−3^	2.6 × 10^−6^	1.9 × 10^−7^	6.7 × 10^−8^	4.9 × 10^−9^	1.1 × 10^−9^	1.0 × 10^−10^	2.7 × 10^−11^
9	1939	1	1	1.7 × 10^−2^	4.6 × 10^−6^	4.2 × 10^−7^	1.6 × 10^−7^	4.9 × 10^−9^	7.7 × 10^−9^	7.8 × 10^−10^	3.3 × 10^−10^
10	1,672	1	1	1.5 × 10^−3^	4.5 × 10^−8^	2.2 × 10^−9^	3.3 × 10^−9^	3.6 × 10^−10^	7.2 × 10^−11^	2.4 × 10^−12^	1.4 × 10^−12^
11	2,857	1	1	1.4 × 10^−1^	1.2 × 10^−5^	7.4 × 10^−7^	5.4 × 10^−7^	1.1 × 10^−7^	1.7 × 10^−8^	1.3 × 10^−9^	2.2 × 10^−10^
12	2,121	1	1	2.5 × 10^−2^	1.0 × 10^−7^	5.6 × 10^−9^	1.7 × 10^−9^	2.5 × 10^−10^	7.2 × 10^−11^	5.2 × 10^−12^	3.6 × 10^−12^

Chr	No. Of SNPs in test	*p*-value									
		t = 11	t = 12	t = 13	t = 14	t = 15	t = 16	t = 17	t = 18	t = 19	t = 20
1	6,332	1.2 × 10^−11^	5.7 × 10^−13^	1.2 × 10^−13^	9.9 × 10^−14^	5.7 × 10^−14^	4.0 × 10^−14^	1.5 × 10^−14^	4.3 × 10^−15^	6.9 × 10^−15^	7.5 × 10^−15^
2	3,808	2.0 × 10^−9^	1.5 × 10^−10^	2.9 × 10^−11^	1.0 × 10^−11^	5.1 × 10^−12^	1.3 × 10^−11^	4.1 × 10^−12^	1.4 × 10^−12^	1.3 × 10^−12^	8.7 × 10^−13^
3	4,298	2.9 × 10^−14^	2.9 × 10^−15^	3.3 × 10^−16^	2.9 × 10^−16^	3.8 × 10^−17^	6.0 × 10^−17^	7.6 × 10^−18^	3.8 × 10^−18^	4.8 × 10^−18^	8.1 × 10^−19^
4	2,802	5.0 × 10^−10^	6.5 × 10^−11^	4.1 × 10^−11^	1.6 × 10^−11^	5.5 × 10^−12^	1.4 × 10^−11^	3.3 × 10^−12^	1.5 × 10^−12^	3.5 × 10^−12^	9.3 × 10^−13^
5	2,800	5.8 × 10^−12^	4.5 × 10^−13^	2.8 × 10^−13^	1.7 × 10^−13^	7.4 × 10^−14^	1.2 × 10^−13^	1.4 × 10^−13^	9.3 × 10^−14^	2.4 × 10^−13^	2.9 × 10^−13^
6	3,177	2.4 × 10^−13^	1.4 × 10^−14^	2.5 × 10^−15^	1.0 × 10^−15^	9.1 × 10^−16^	2.0 × 10^−15^	3.2 × 10^−16^	1.5 × 10^−16^	2.3 × 10^−16^	9.4 × 10^−17^
7	2024	2.0 × 10^−13^	1.6 × 10^−14^	3.2 × 10^−14^	3.8 × 10^−14^	3.8 × 10^−15^	1 × 10^−14^	8.3 × 10^−15^	6.8 × 10^−15^	5.0 × 10^−15^	1.4 × 10^−15^
8	2,233	1.4 × 10^−10^	1.6 × 10^−11^	1.3 × 10^−11^	6.7 × 10^−12^	2.3 × 10^−12^	3.8 × 10^−12^	3.5 × 10^−12^	2.4 × 10^−12^	4.5 × 10^−12^	2.0 × 10^−12^
9	1939	1.5 × 10^−9^	1.2 × 10^−10^	5.4 × 10^−11^	3.4 × 10^−11^	3.8 × 10^−12^	7.4 × 10^−12^	2.9 × 10^−12^	1.0 × 10^−12^	8.6 × 10^−13^	3.0 × 10^−13^
10	1,672	4.2 × 10^−12^	2.7 × 10^−13^	1.6 × 10^−13^	6.1 × 10^−14^	1.0 × 10^−14^	1.1 × 10^−14^	4.5 × 10^−15^	2.7 × 10^−15^	6.3 × 10^−15^	5.2 × 10^−15^
11	2,857	1.6 × 10^−10^	1.2 × 10^−11^	3.7 × 10^−12^	1.9 × 10^−12^	2.0 × 10^−13^	3.7 × 10^−13^	1.5 × 10^−13^	6.9 × 10^−14^	1.2 × 10^−13^	5.9 × 10^−14^
12	2,121	1.0 × 10^−11^	8.5 × 10^−13^	1.6 × 10^−13^	4.8 × 10^−14^	5.7 × 10^−14^	2.3 × 10^−13^	8.3 × 10^−14^	1.0 × 10^−13^	3.4 × 10^−13^	5.0 × 10^−13^

The calculation of the whole process was taken 15 s on the Intel Core 3.40 GHz CPU. This result indicates that the genetic region association analysis method based on the LFDAT method is computationally feasible.

## 5 Discussion

Considering the association analysis of quantitative traits at multiple time points, it is possible to better observe the influence of time-changing genes on quantitative traits. Further, longitudinal trait research based on gene regions can improve the power. We propose the LFDAT method and that the function-on-function regression model is applied to detect the association between gene regions and longitudinal traits. This can simultaneously lead to continuous phenotypic traits and marker information and make full use of the information carried by the traits to explore the influence of genes on longitudinal traits. Compared with other dynamic association analysis methods, the LFDAT method considers the genetic effects of variants in the entire gene region and the time effects of genes. It can also accurately detect the selective expression function of genes. The gene region association analysis based on the LFDAT method has few restrictions on the direction of gene effects, low computational cost, fast detection speed, low false positives, and high power. It further has a stronger explanatory for the effect of genes on the quantitative traits concerning time.

We consider linkage equilibrium simulation and linkage disequilibrium simulation, the powers of the five gene regions are compared to prove the feasibility of LFDAT for a longitudinal trait association analysis in two simulation studies. At the same time, two cases are set for the time-varying function of the genetic effects to explore whether LFDAT can detect the selective expression of genes at different time points. The simulated results show that LFDAT has a lower type I error rates and higher power on the association analysis of the five gene regions and can accurately detect the selective expression of genes in two simulations. In addition, different settings for the variance and correlation coefficient of the random error are simulated. When the variance is 25, compared with the variance of 1, the powers of linkage equilibrium and linkage disequilibrium are significantly reduced, three indicators of linkage disequilibrium increase, and ISE0 and PMSE of linkage equilibrium increase. However, ISE1 of linkage equilibrium decrease. When the correlation coefficient is 0.95, compared with the correlation coefficient of 0.5, power of linkage disequilibrium increase, and three indicators of linkage disequilibrium decrease, however the changes of power and three indicators of linkage equilibrium are not obvious.

For the continuous effect function, we try to plot the figure the estimated time-varying function of the genetic effect in linkage equilibrium and linkage disequilibrium simulation at first time point, in which time effect function and genetic effect function are fixed to constants and causal variants are 55, 66, 77, 88, 99, 110, 121, 132, 143, and 154-th SNP respectively. We find that the fitting of the time-varying function of the genetic effect in the linkage disequilibrium simulation is smoother than that of the linkage equilibrium simulation in most figures (As shown in Supplementary Data S7). This is because there is an association between each SNP, which makes the fitting of the time-varying function more constrained. Furthermore, Haseman and Elston (1972) proposed a linear model for detecting linkage between a marker and a QTL in a full-sib design. Then, the mathematical expectation of the regression coefficient is expressed by the additive genetic variance of the QTL. Chen (2014, 2016) proposed that variance component methods, such as the Haseman-Elston (HE) regression and the linear mixed model (LMM), provide valid estimate of heritability based gene effect in GWAS data for complex traits. The estimated heritability may reveal the genetic architecture underlying a complex trait. For the study about between the heritability and the continuous effect function within a gene region based on functional data analysis, there is currently no relevant research in this field. In the future, we will conduct in-depth research in this direction.

Of course, LFDA that converts gene loci into continuous variables has some shortcomings. First, the covariates, population structure, and locus weights are not considered. In gene regions, the weak effects of rare variants are difficult to find, making it challenging to identify the gene regions of rare variants. The common solution is to assign different weights to different types of variants. In the research on LSKAT and LBT methods proposed by Wang et al. (2017), covariates and population structures were considered, and common and rare variants were given different weights. We sought to study the growth and development mechanism of plants in this paper mainly. However, we could consider adding factors, such as the population structure, and introduce the idea of weight to improve the detection ability of LFDAT in future research.

Second, the fitting errors of indicators ISE0, ISE1, and PMSE are relatively large. It might be because of the limitations of LFDAT, which cannot compress the time-varying function of genetic effects to a state close to null, as stated by Lin et al. (2017). This is one direction of our future research.

Third, in the simulation of the selective gene expression, it can be seen that the powers of linkage equilibrium for five gene region are unstable, and the powers are lower at some time points of the gene opening. We find it is related to the time-varying function of genetic effects by simulating a different time-varying function of the genetic effects. Therefore, accurately grasping how genetic effects change over time is a direction worth studying.

Fourth, for the application on the PSA of the Oryza sativa data set, no significant SNP loci are detected at the first two time points. This indicates that the gene region association analysis based on LFDAT needs to be further improved to make the detection effect more accurate. Then, it could be better applied to the gene region association analysis of different longitudinal traits.

This paper applies the LFDAT method to the real data process, and each chromosome is analyzed as an independent gene region. However, the variants that control longitudinal traits might be distributed in different gene regions. If there is a correlation between the causal variants in different gene regions, it is necessary to perform association analysis on multiple gene regions. This is also true if each chromosome is regarded as a gene region and the region is too large to accurately detect genes that control longitudinal traits. Then, the SNP sequence needs to be refined into multiple gene regions. The extension of the longitudinal trait association analysis based on the functional data analysis to multiple gene regions will be a future research direction.

## Data Availability

Publicly available datasets were analyzed in this study. The data can be found here: http://www.ricediversity.org/ and https://doi.org/10.5281/zenodo.1313684.
