# Supplementary figures and images for: Gene Region Association Analysis of Longitudinal Quantitative Traits Based on a Function-On-Function Regression Model (part 2 of 2)
Source: Front Genet. 2022 Feb 21;13:781740. doi: 10.3389/fgene.2022.781740 (PMC8899465; doi:10.3389/fgene.2022.781740)

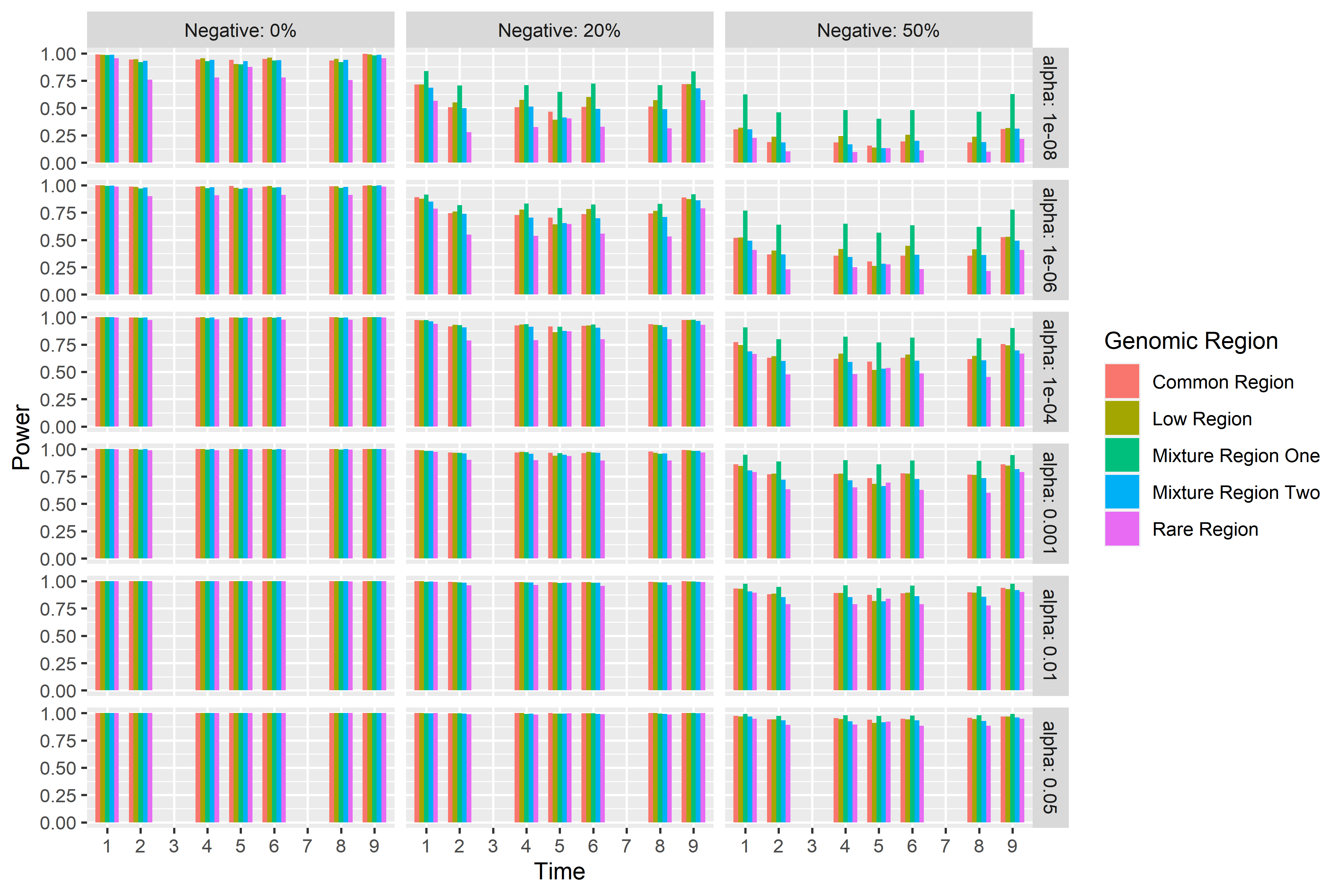

Supplement: Supplementary file 1 [file DataSheet1.ZIP › data in brief/S4/Sample 2000(Case2), c is 3 and the proportion of causal variants is 2%.png]

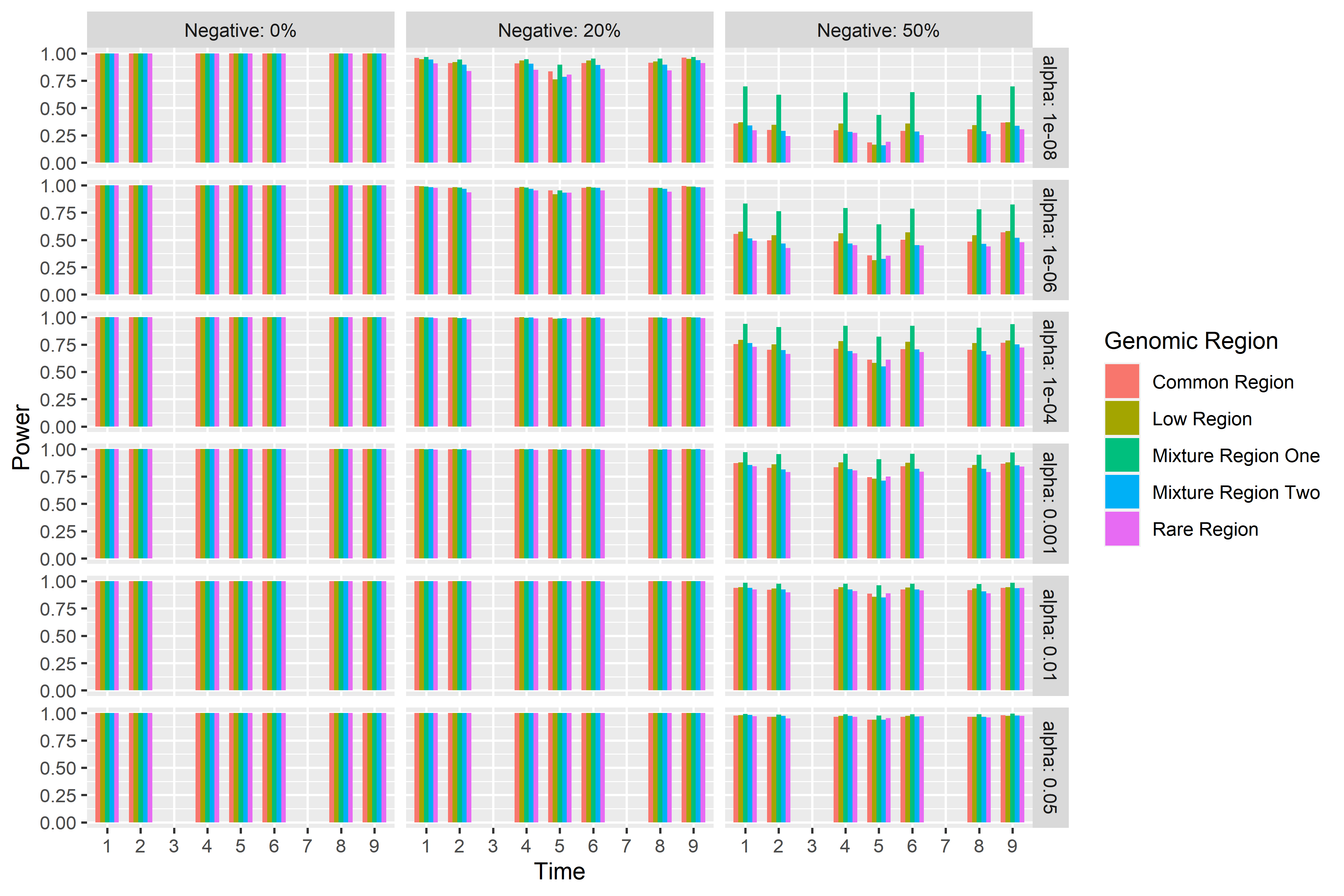

Supplement: Supplementary file 1 [file DataSheet1.ZIP › data in brief/S4/Sample 2000(Case2), c is 3 and the proportion of causal variants is 4%.png]

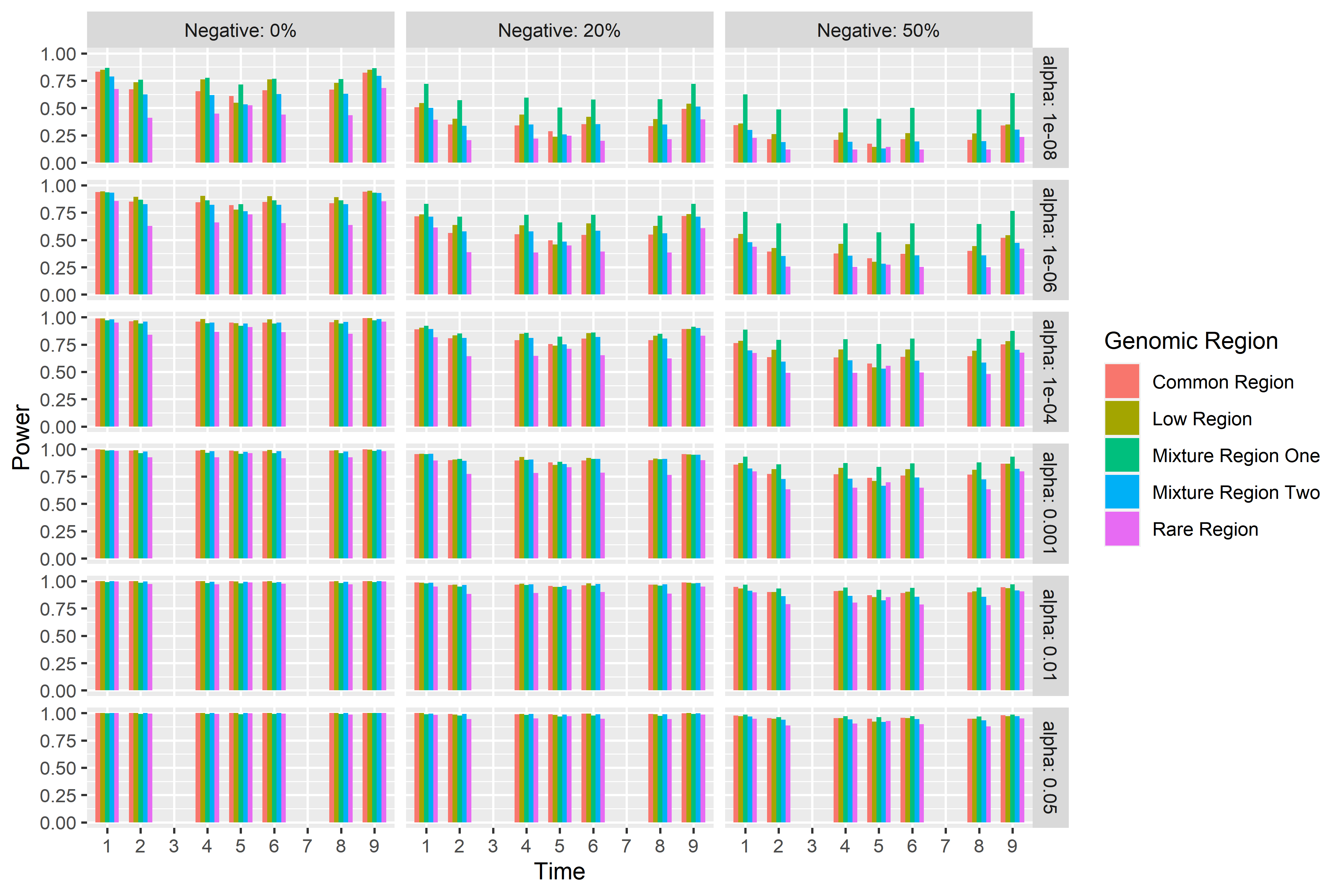

Supplement: Supplementary file 1 [file DataSheet1.ZIP › data in brief/S4/Sample 2000(Case2), c is 5 and the proportion of causal variants is 1%.png]

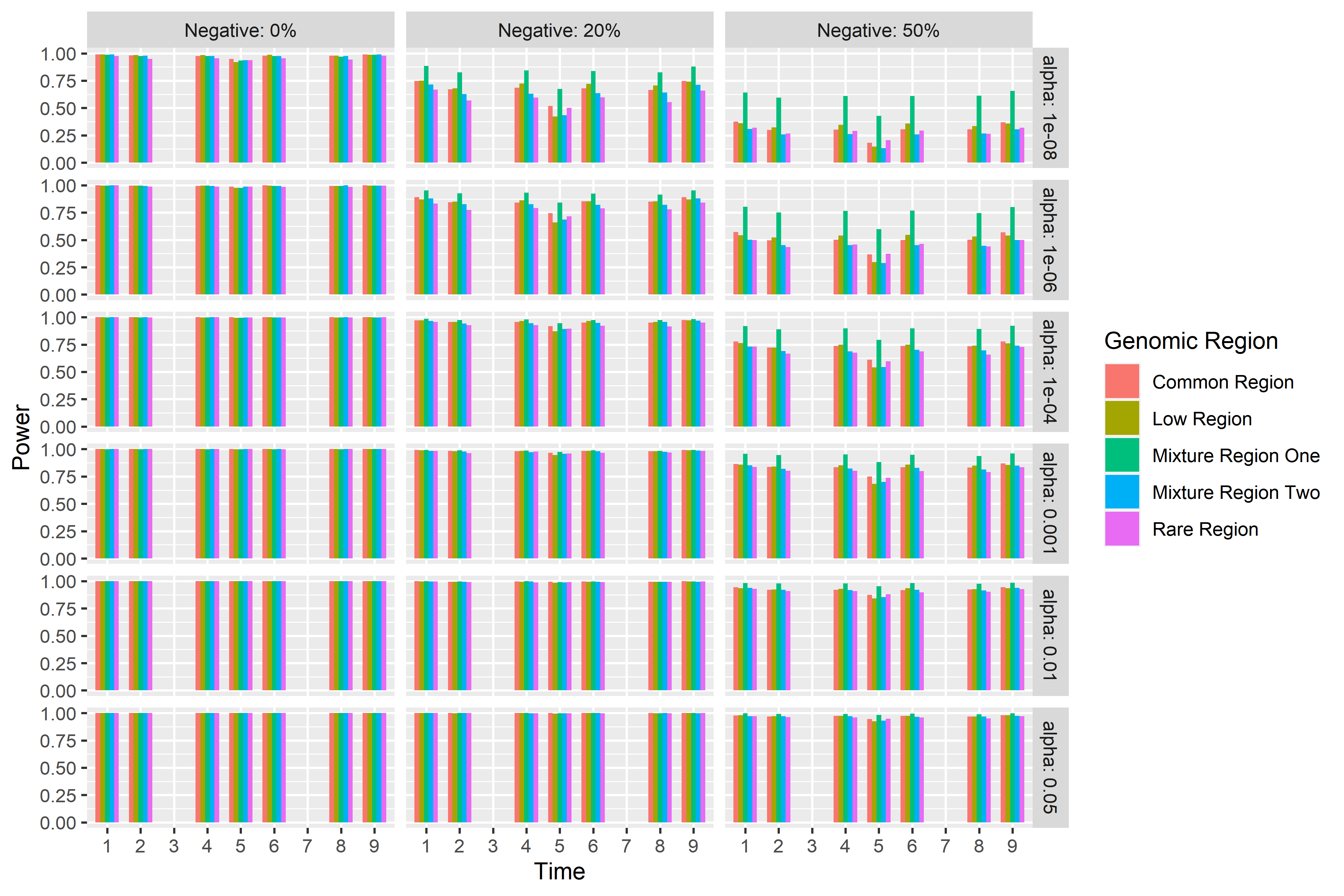

Supplement: Supplementary file 1 [file DataSheet1.ZIP › data in brief/S4/Sample 2000(Case2), c is 5 and the proportion of causal variants is 2%.png]

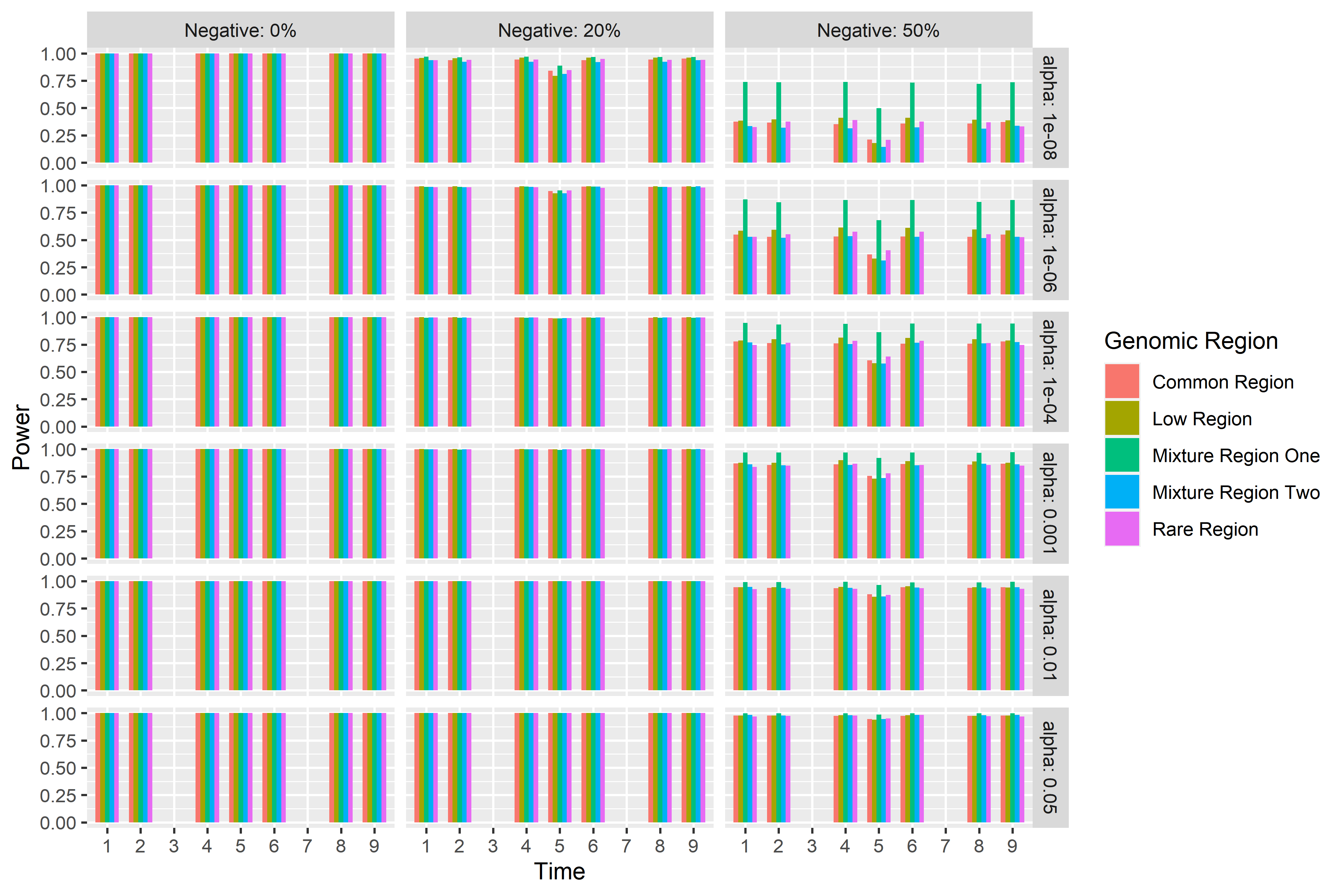

Supplement: Supplementary file 1 [file DataSheet1.ZIP › data in brief/S4/Sample 2000(Case2), c is 5 and the proportion of causal variants is 4%.png]

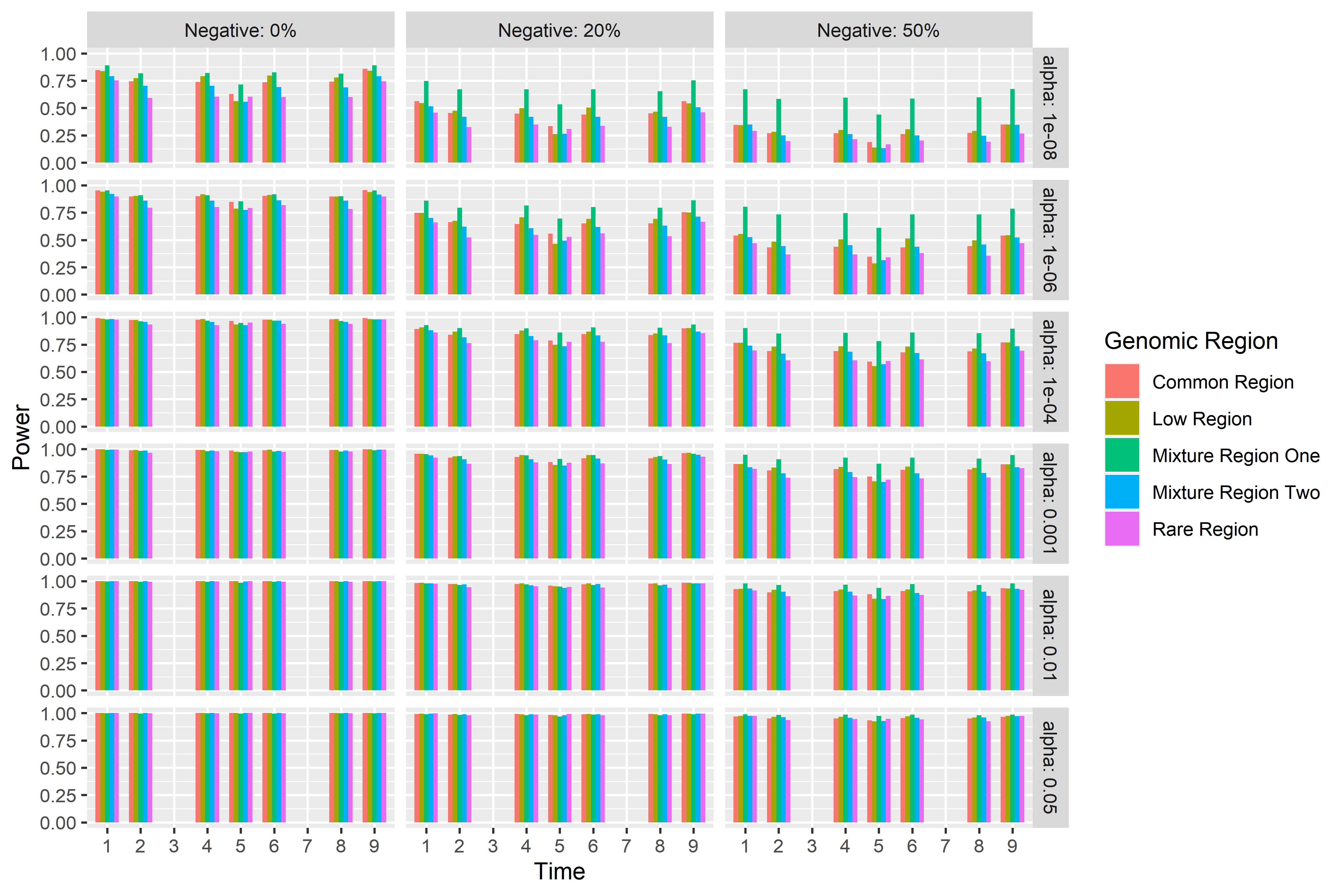

Supplement: Supplementary file 1 [file DataSheet1.ZIP › data in brief/S4/Sample 2000(Case2), c is 7 and the proportion of causal variants is 1%.png]

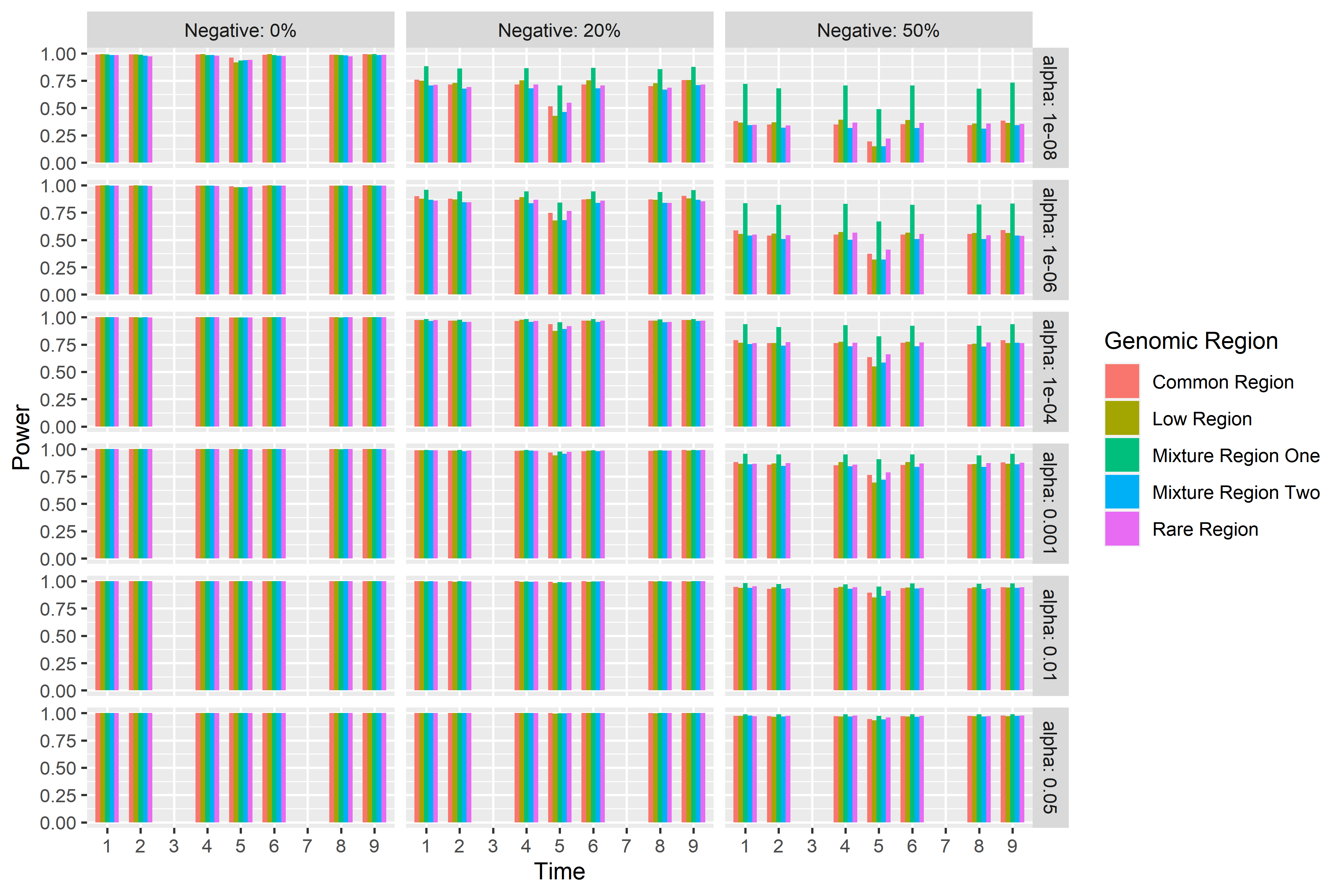

Supplement: Supplementary file 1 [file DataSheet1.ZIP › data in brief/S4/Sample 2000(Case2), c is 7 and the proportion of causal variants is 2%.png]

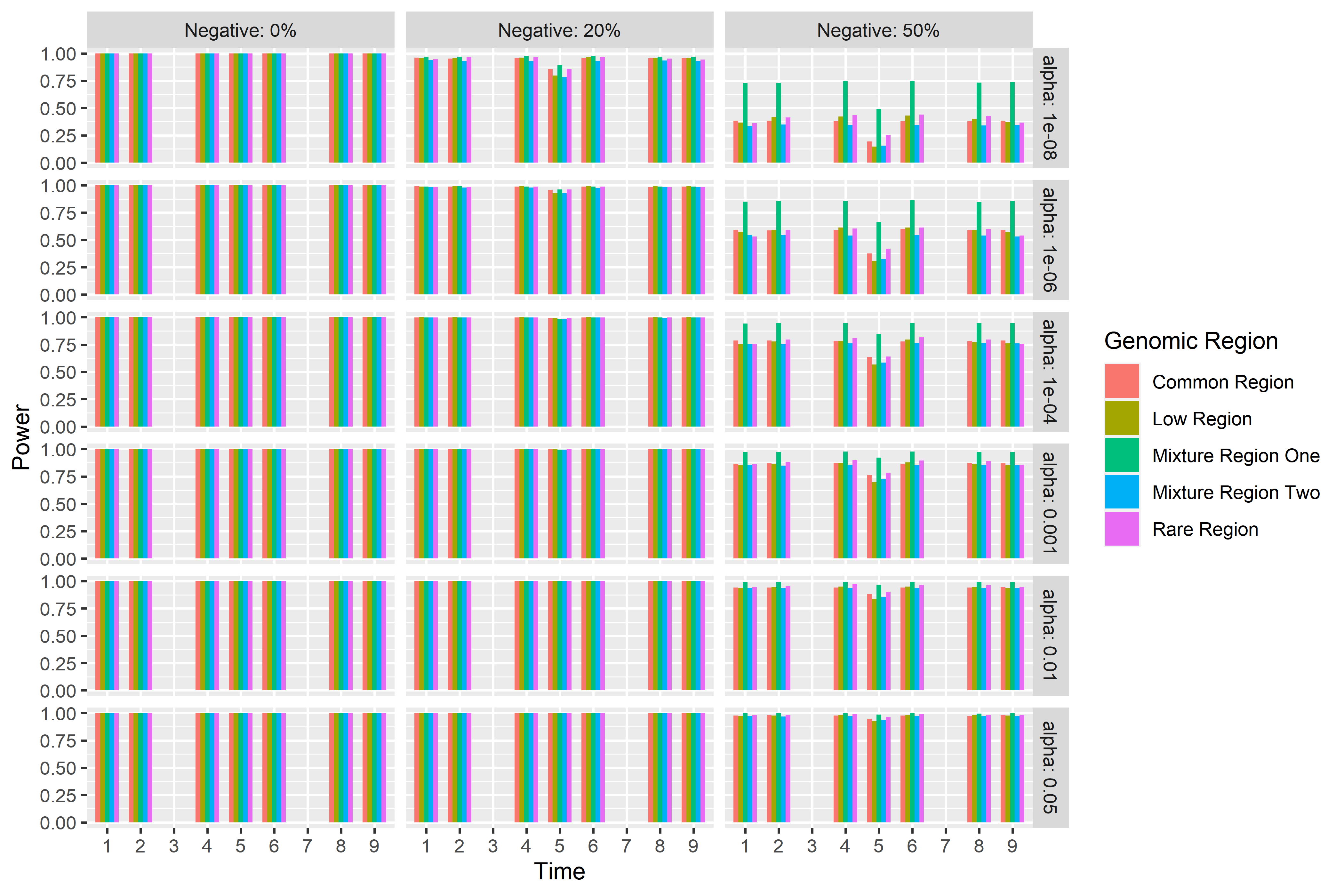

Supplement: Supplementary file 1 [file DataSheet1.ZIP › data in brief/S4/Sample 2000(Case2), c is 7 and the proportion of causal variants is 4%.png]

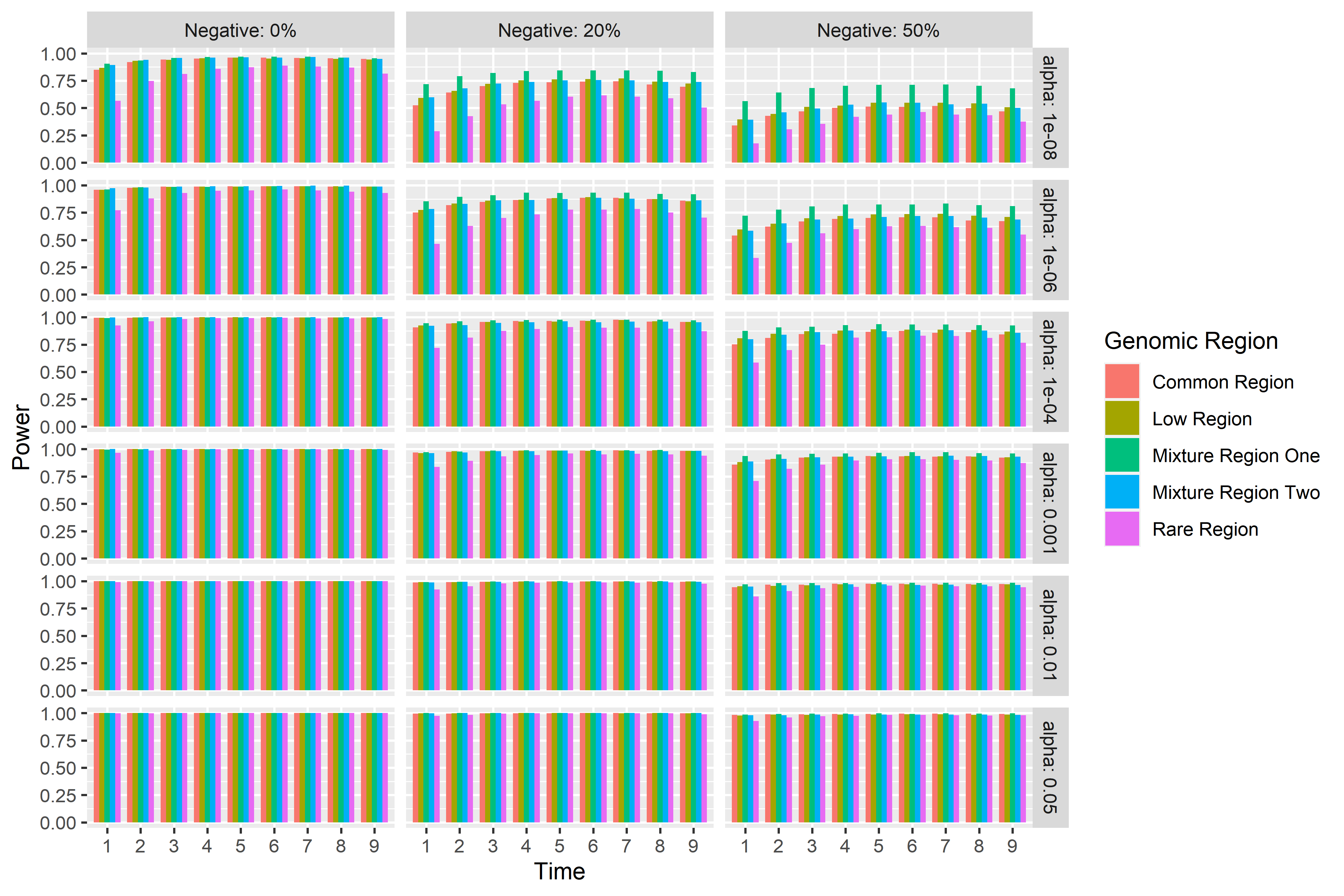

Supplement: Supplementary file 1 [file DataSheet1.ZIP › data in brief/S5/Sample 1000(Case1), c is 3 and the proportion of causal variants is 1%.png]

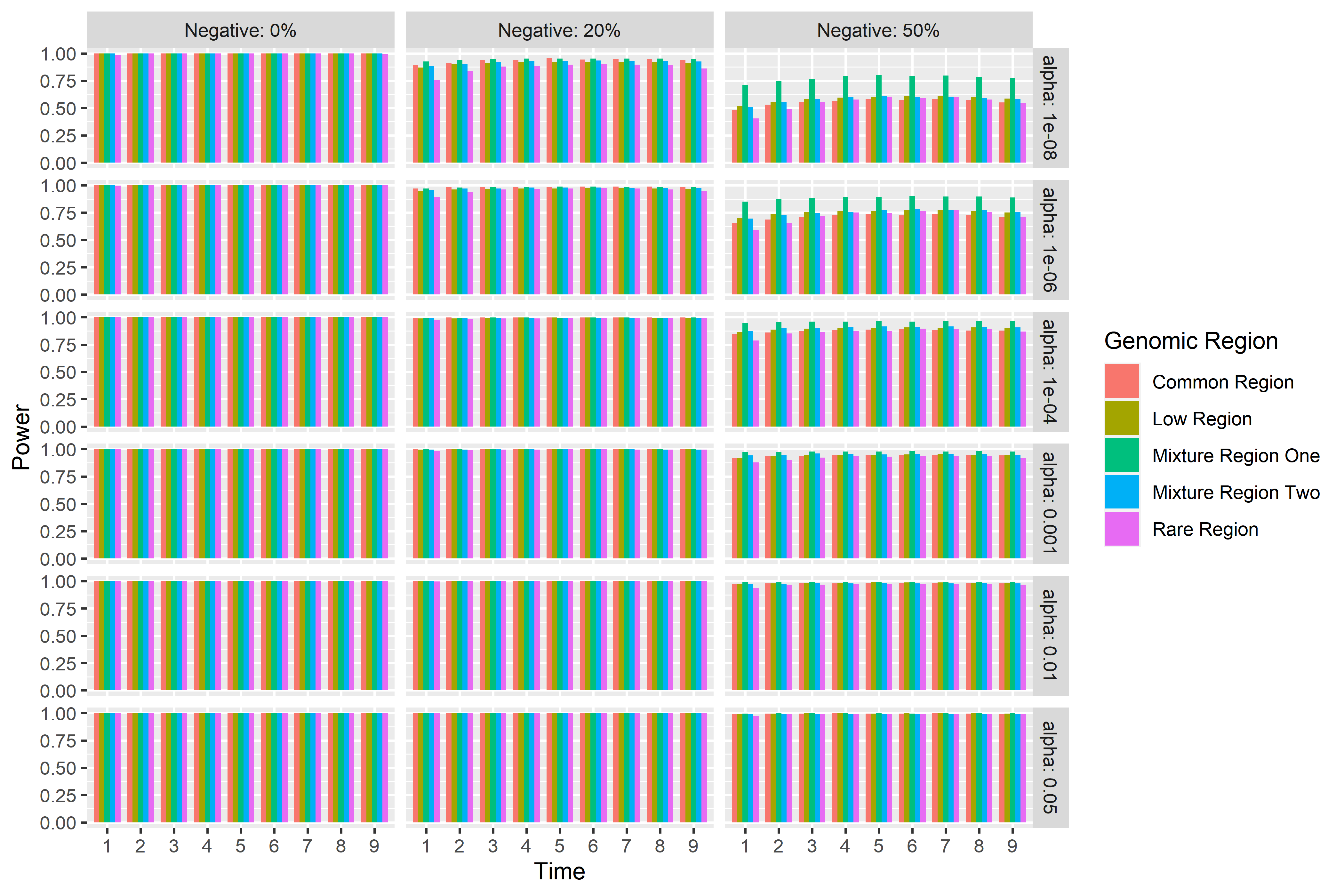

Supplement: Supplementary file 1 [file DataSheet1.ZIP › data in brief/S5/Sample 1000(Case1), c is 3 and the proportion of causal variants is 2%.png]

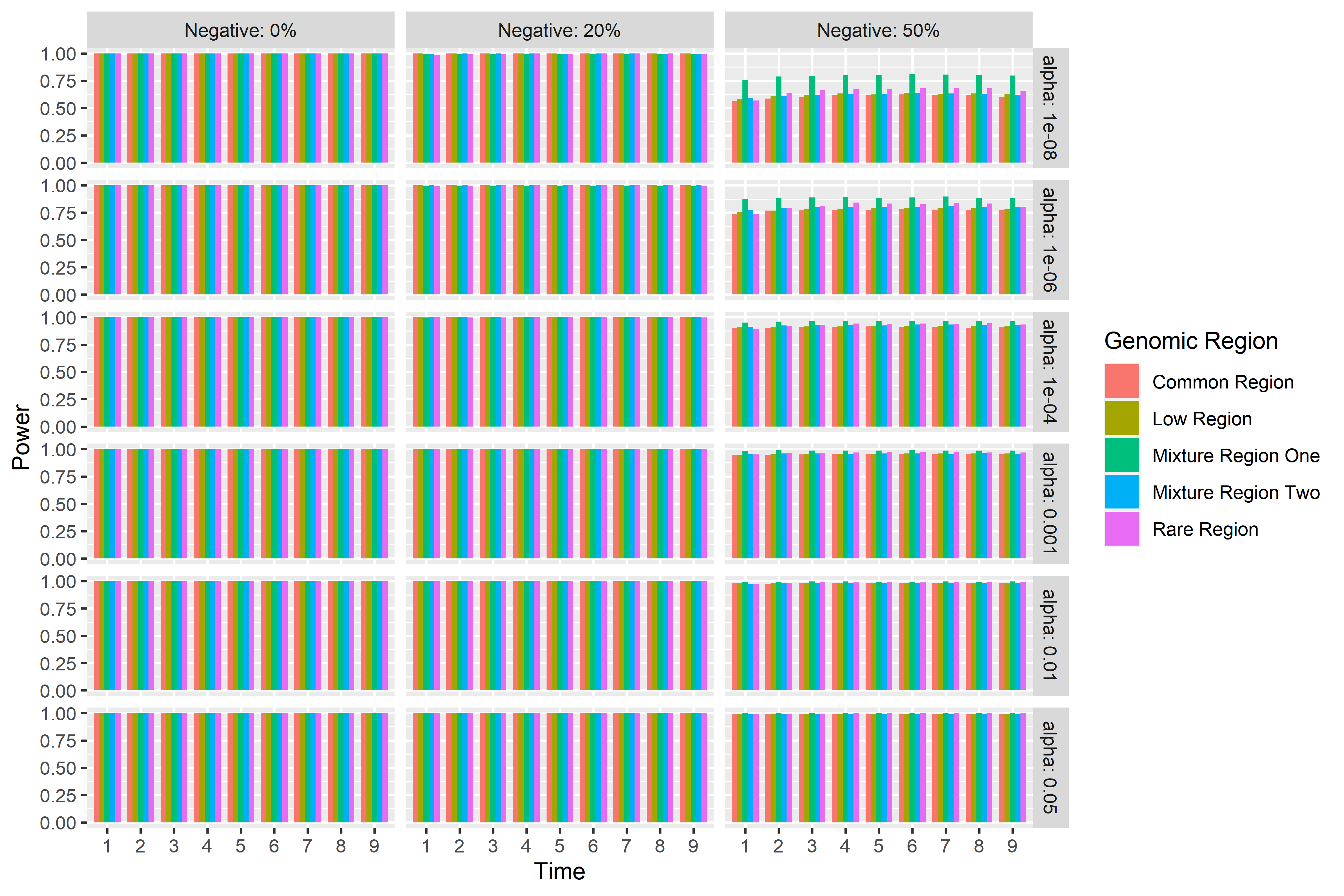

Supplement: Supplementary file 1 [file DataSheet1.ZIP › data in brief/S5/Sample 1000(Case1), c is 3 and the proportion of causal variants is 4%.png]

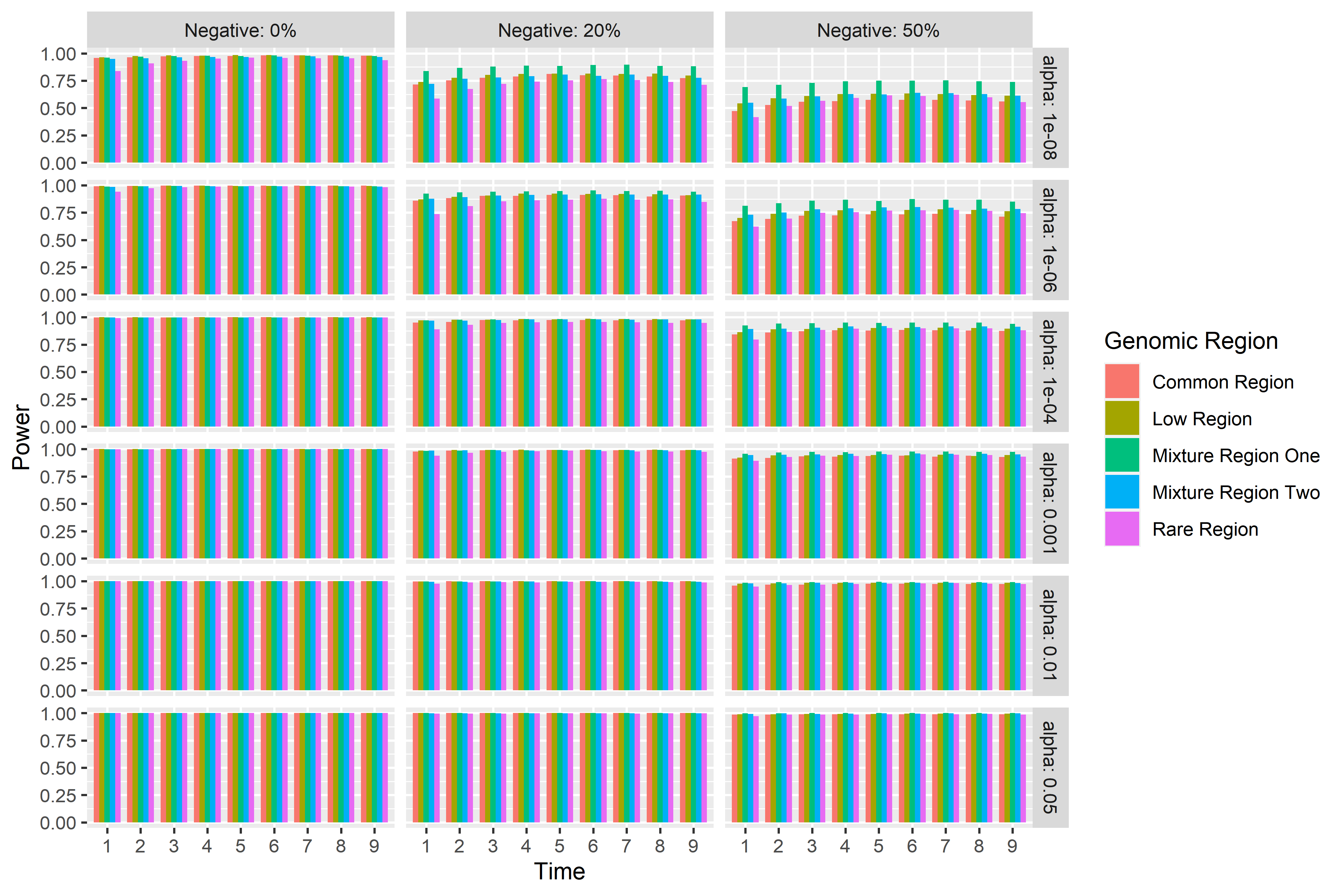

Supplement: Supplementary file 1 [file DataSheet1.ZIP › data in brief/S5/Sample 1000(Case1), c is 5 and the proportion of causal variants is 1%.png]

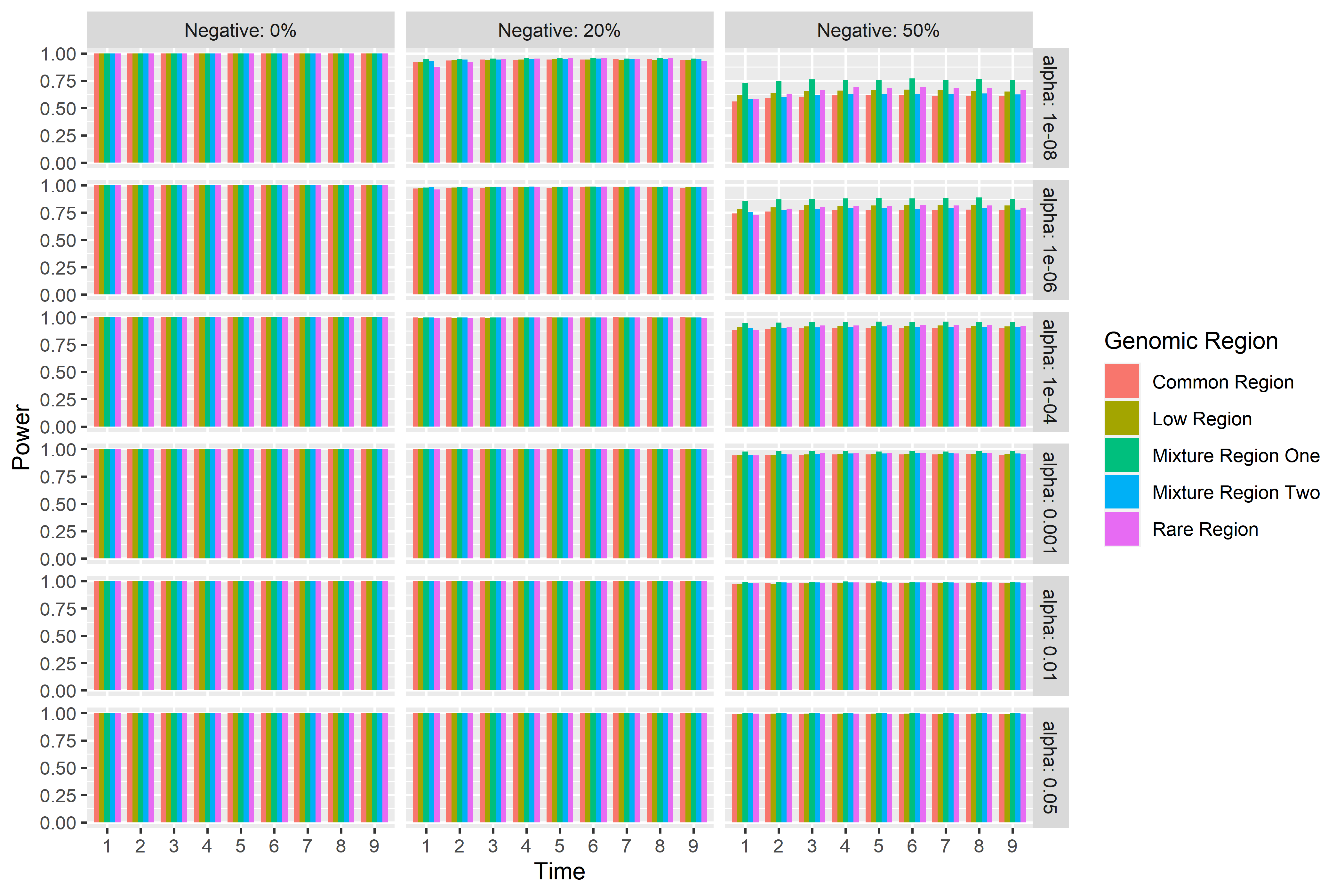

Supplement: Supplementary file 1 [file DataSheet1.ZIP › data in brief/S5/Sample 1000(Case1), c is 5 and the proportion of causal variants is 2%.png]

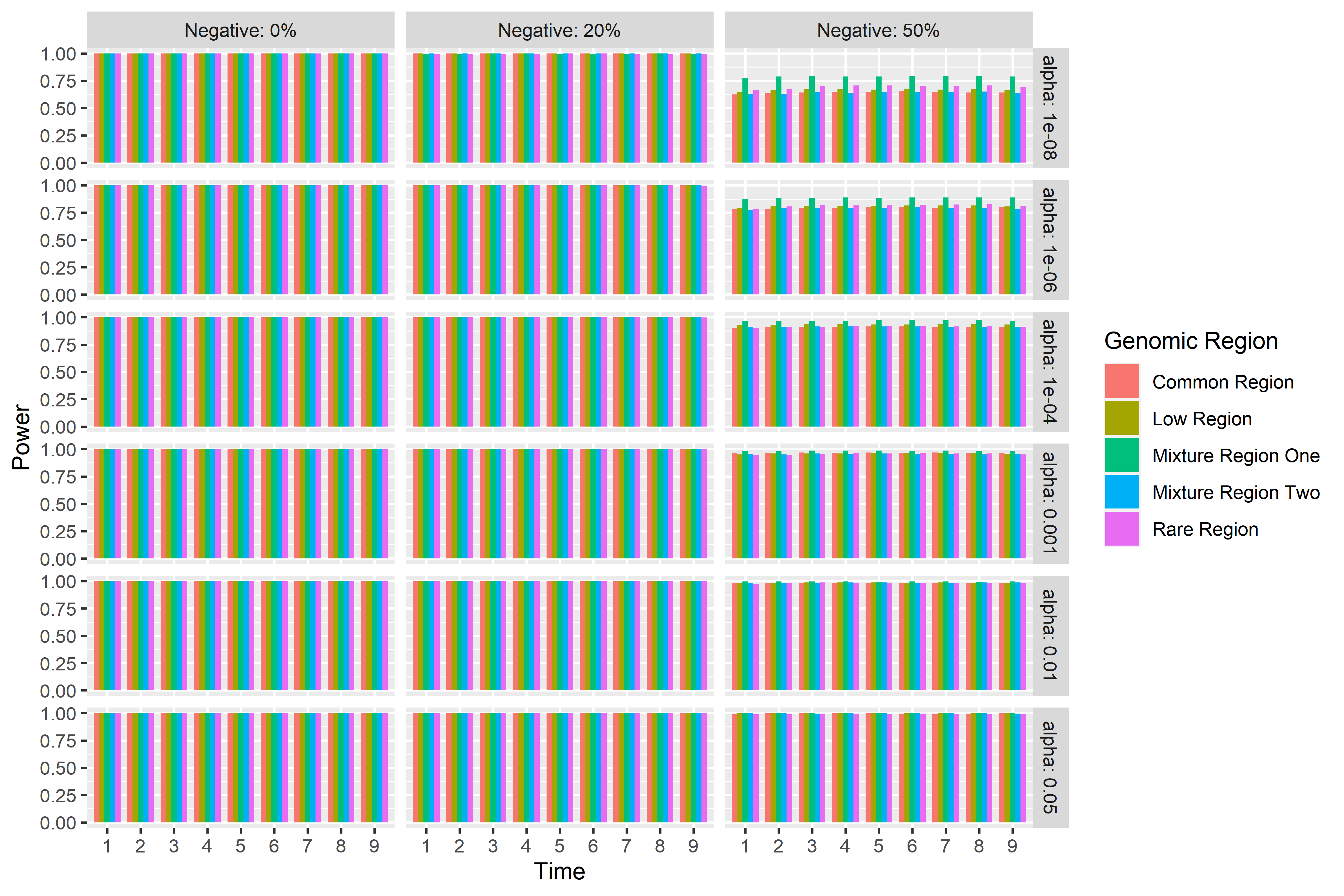

Supplement: Supplementary file 1 [file DataSheet1.ZIP › data in brief/S5/Sample 1000(Case1), c is 5 and the proportion of causal variants is 4%.png]

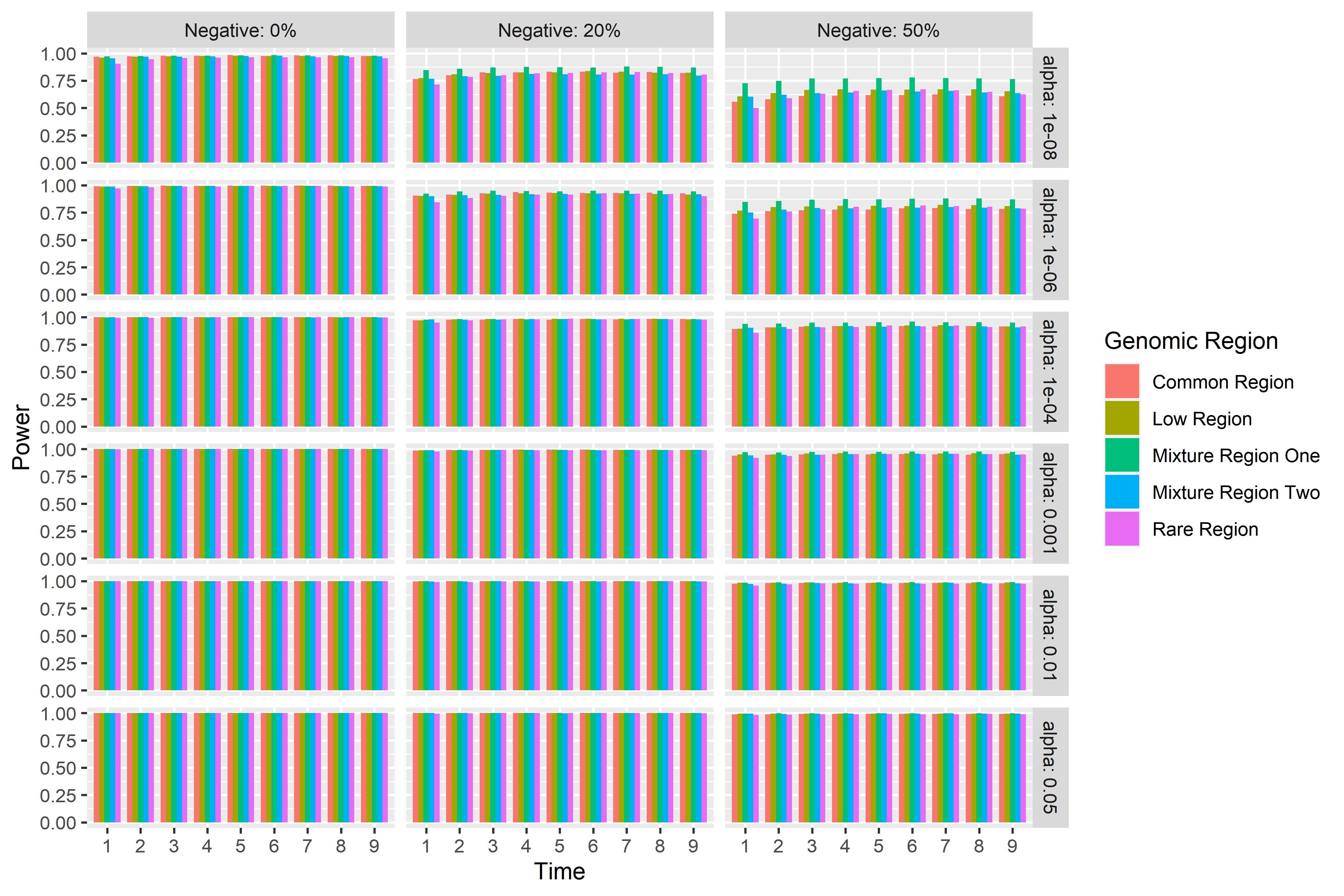

Supplement: Supplementary file 1 [file DataSheet1.ZIP › data in brief/S5/Sample 1000(Case1), c is 7 and the proportion of causal variants is 1%.png]

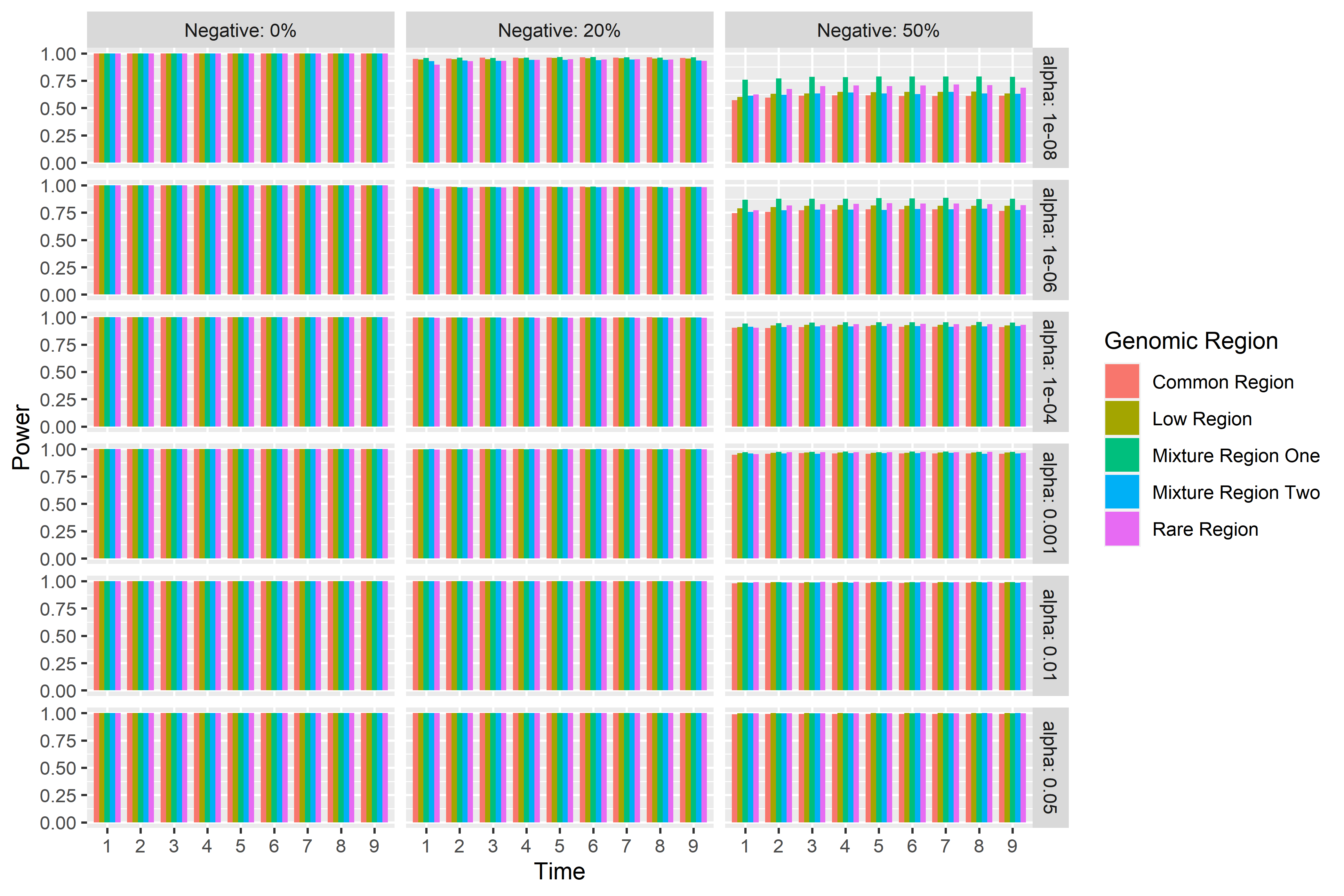

Supplement: Supplementary file 1 [file DataSheet1.ZIP › data in brief/S5/Sample 1000(Case1), c is 7 and the proportion of causal variants is 2%.png]

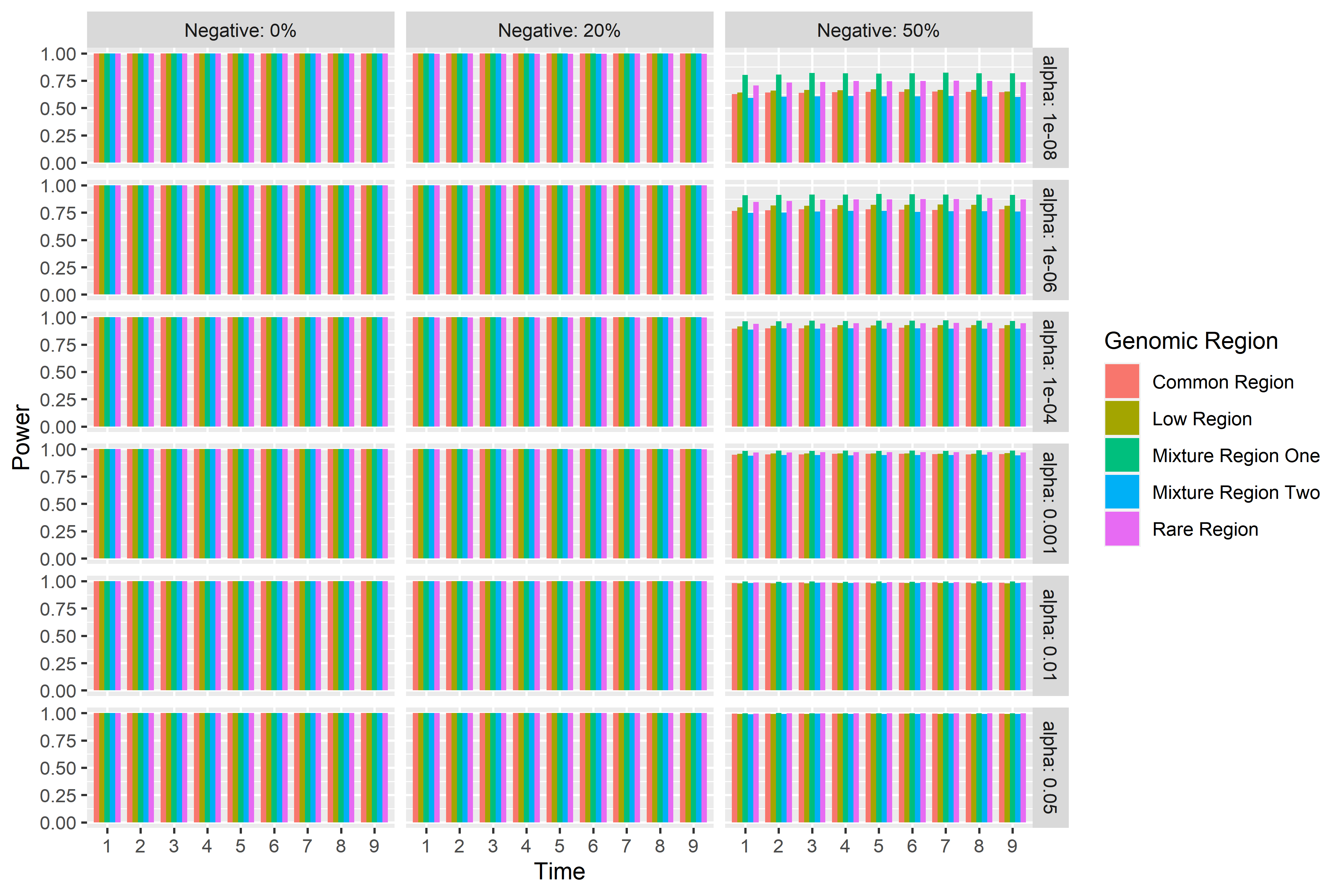

Supplement: Supplementary file 1 [file DataSheet1.ZIP › data in brief/S5/Sample 1000(Case1), c is 7 and the proportion of causal variants is 4%.png]

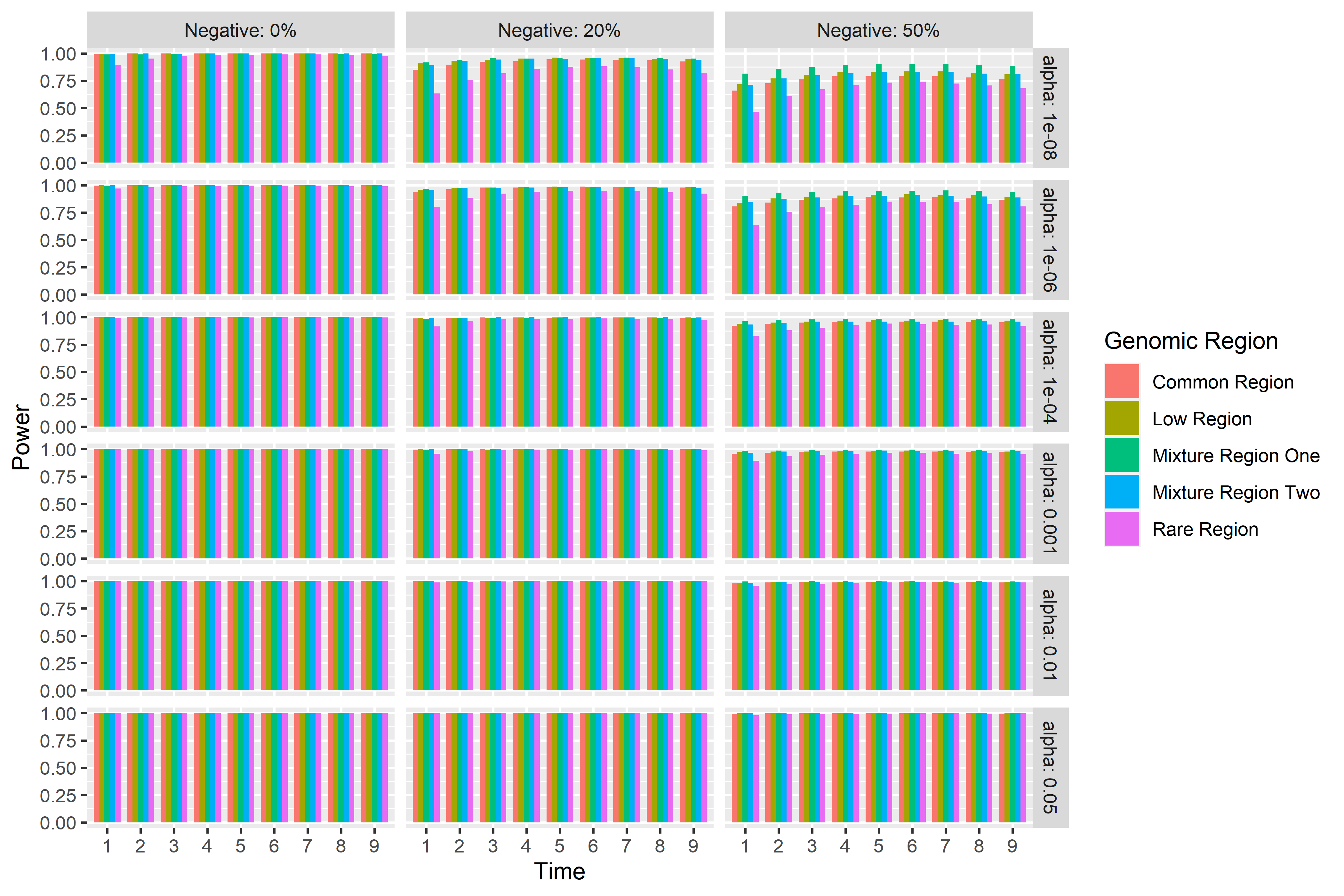

Supplement: Supplementary file 1 [file DataSheet1.ZIP › data in brief/S5/Sample 1500(Case1), c is 3 and the proportion of causal variants is 1%.png]

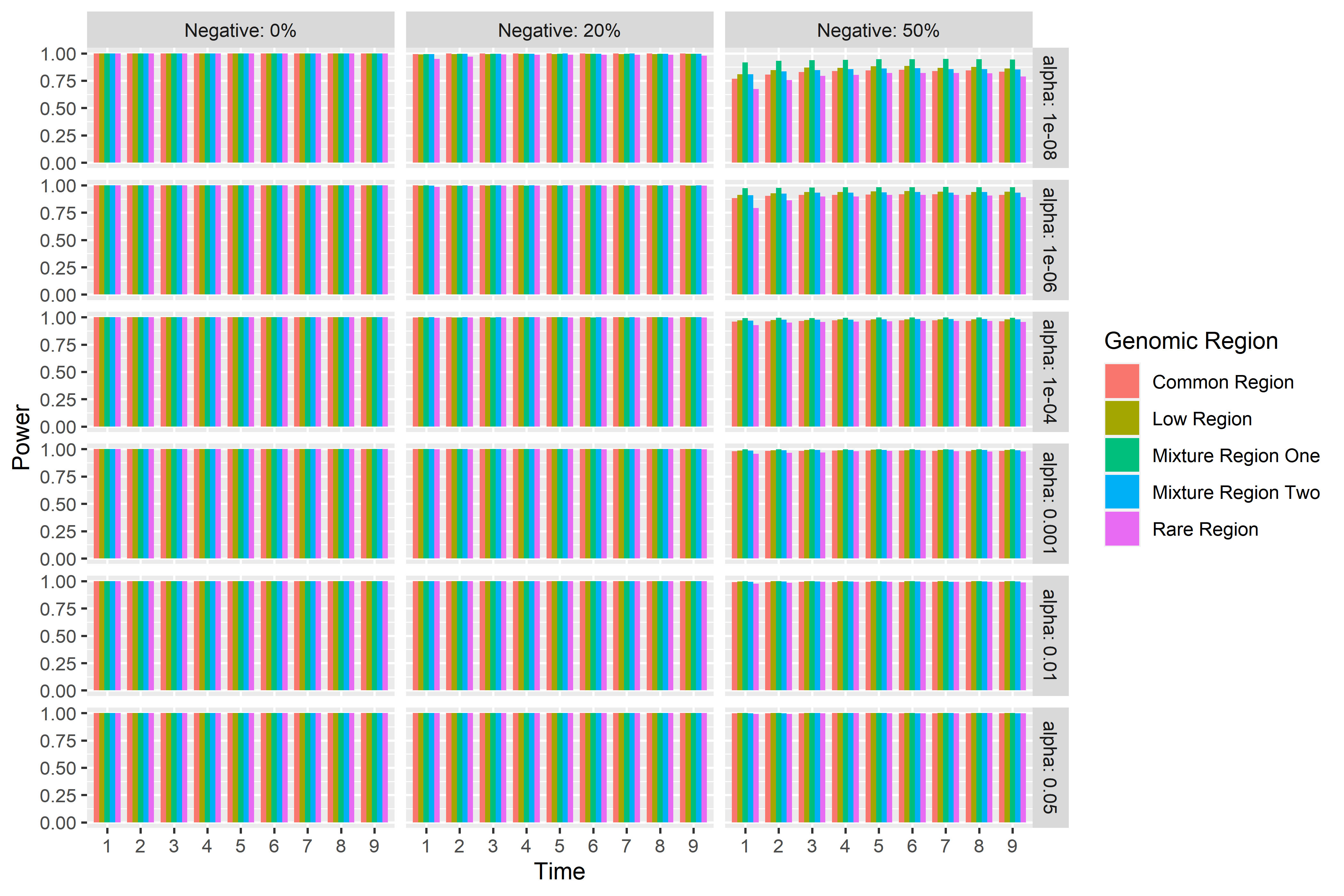

Supplement: Supplementary file 1 [file DataSheet1.ZIP › data in brief/S5/Sample 1500(Case1), c is 3 and the proportion of causal variants is 2%.png]

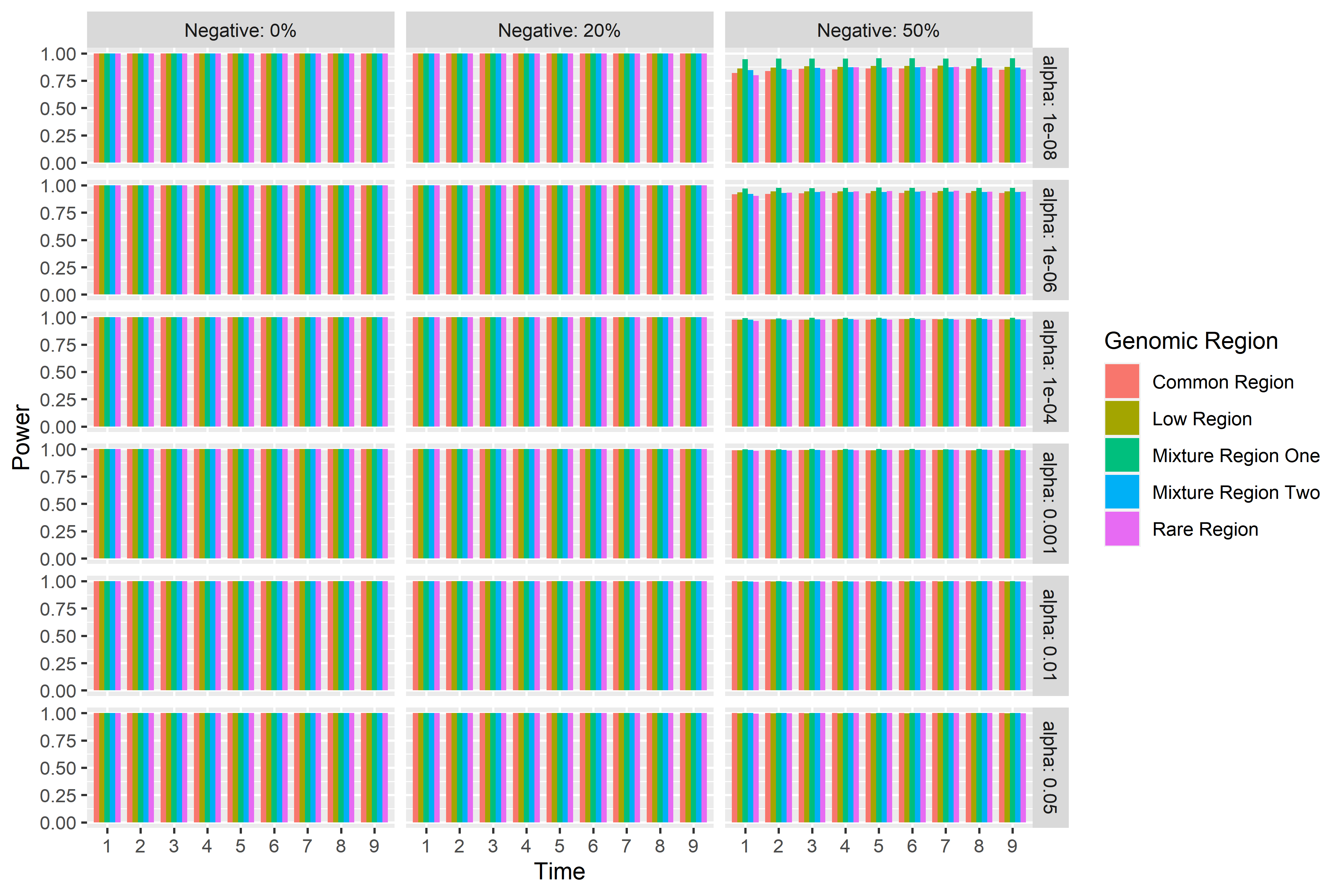

Supplement: Supplementary file 1 [file DataSheet1.ZIP › data in brief/S5/Sample 1500(Case1), c is 3 and the proportion of causal variants is 4%.png]

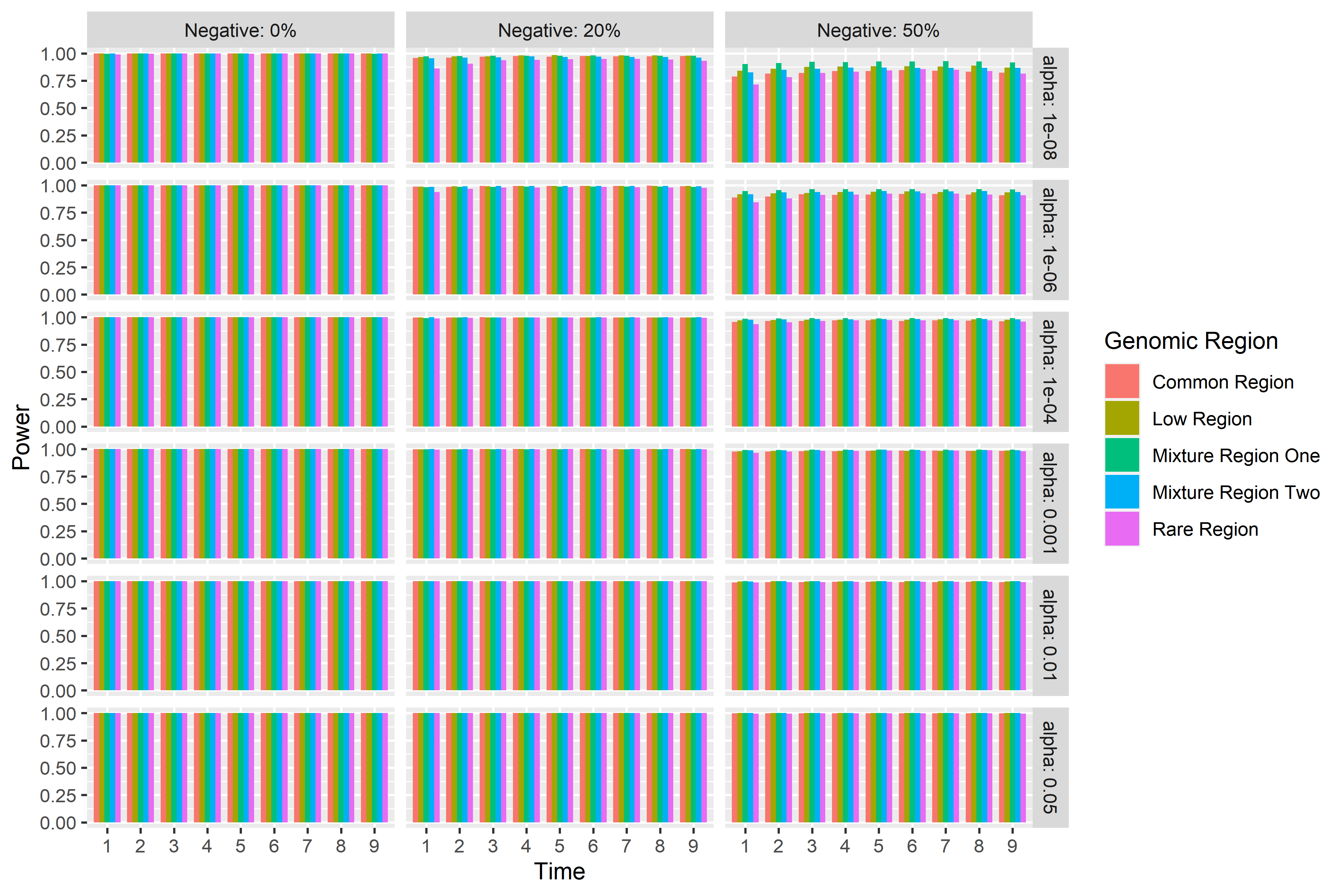

Supplement: Supplementary file 1 [file DataSheet1.ZIP › data in brief/S5/Sample 1500(Case1), c is 5 and the proportion of causal variants is 1%.png]

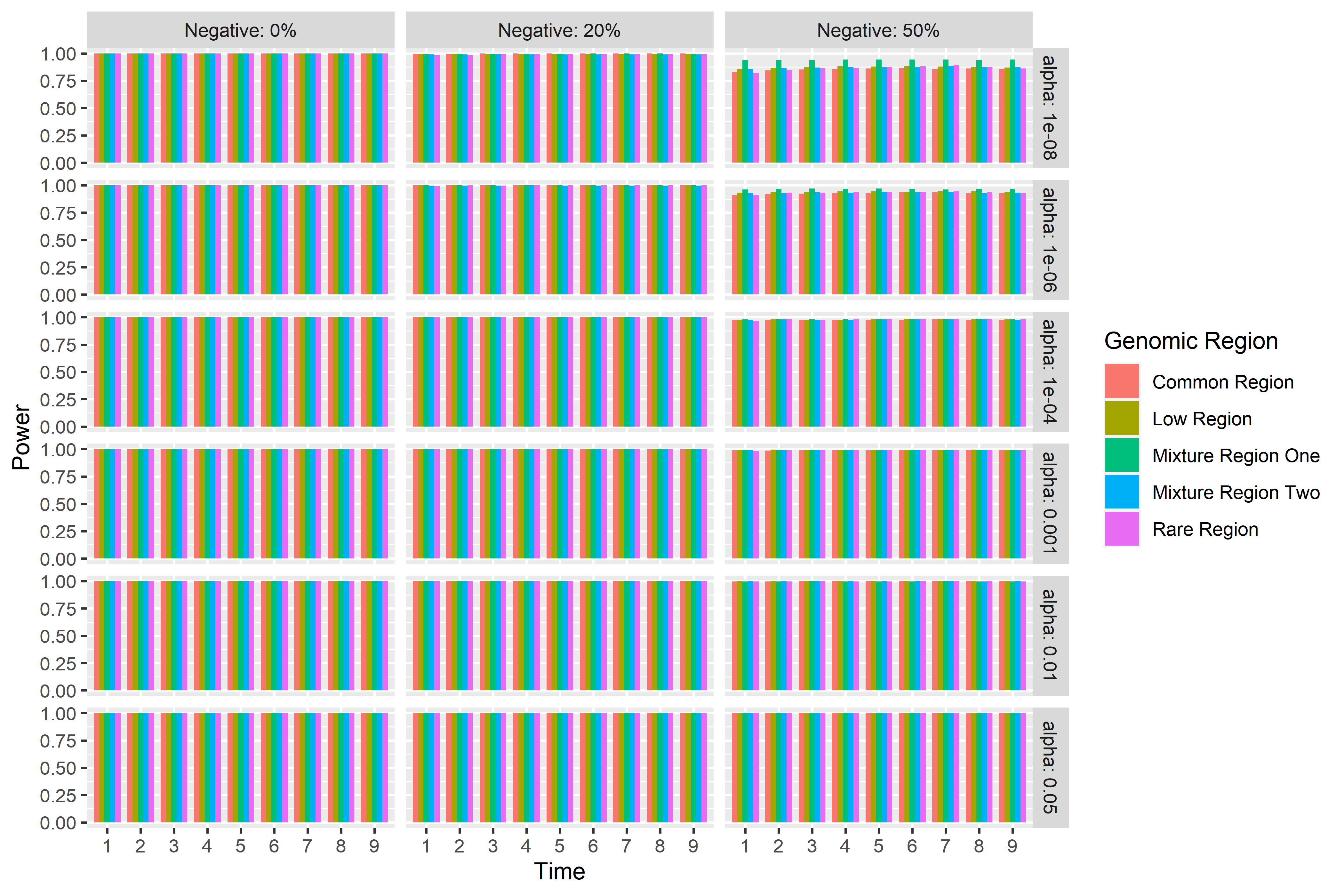

Supplement: Supplementary file 1 [file DataSheet1.ZIP › data in brief/S5/Sample 1500(Case1), c is 5 and the proportion of causal variants is 2%.png]

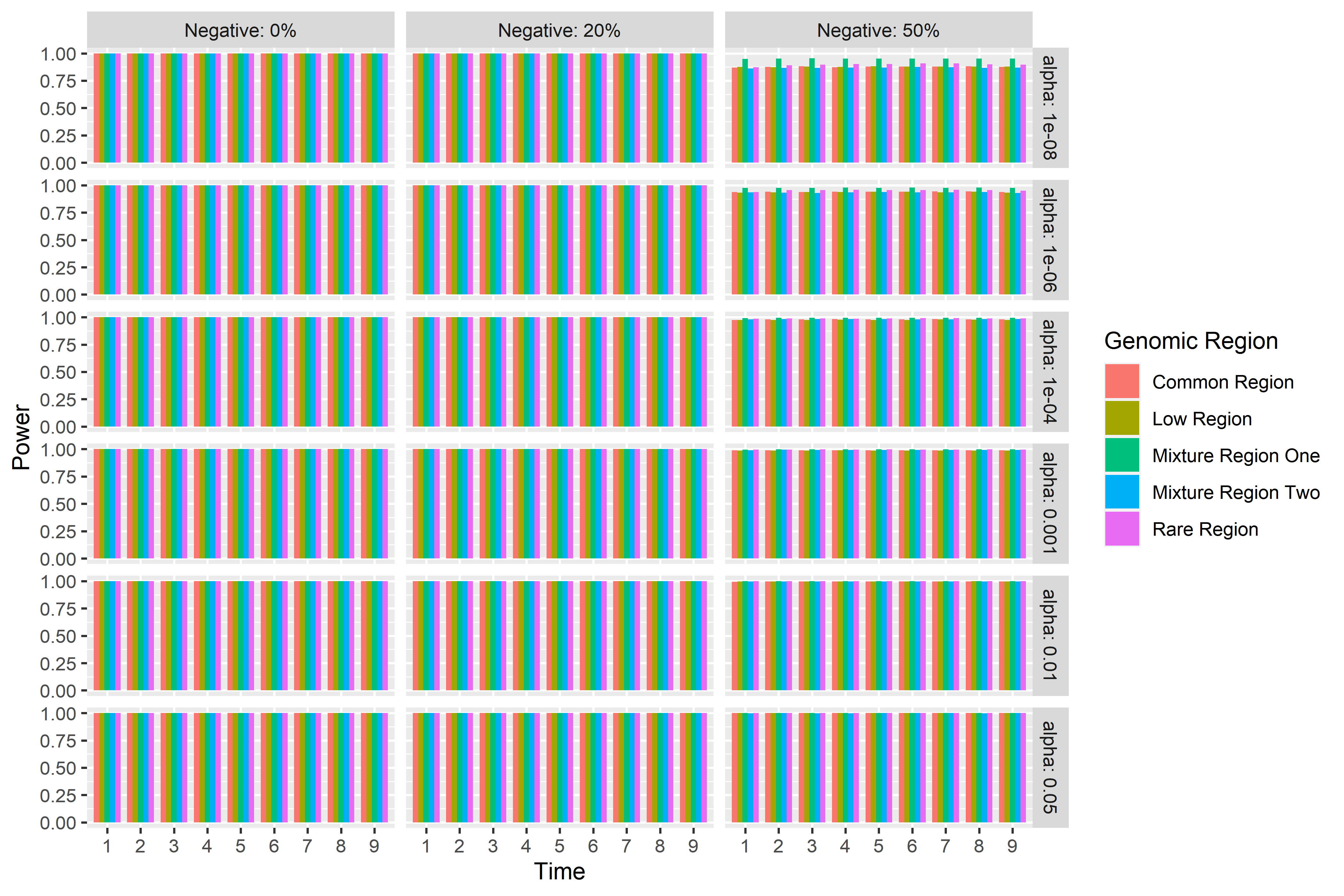

Supplement: Supplementary file 1 [file DataSheet1.ZIP › data in brief/S5/Sample 1500(Case1), c is 5 and the proportion of causal variants is 4%.png]

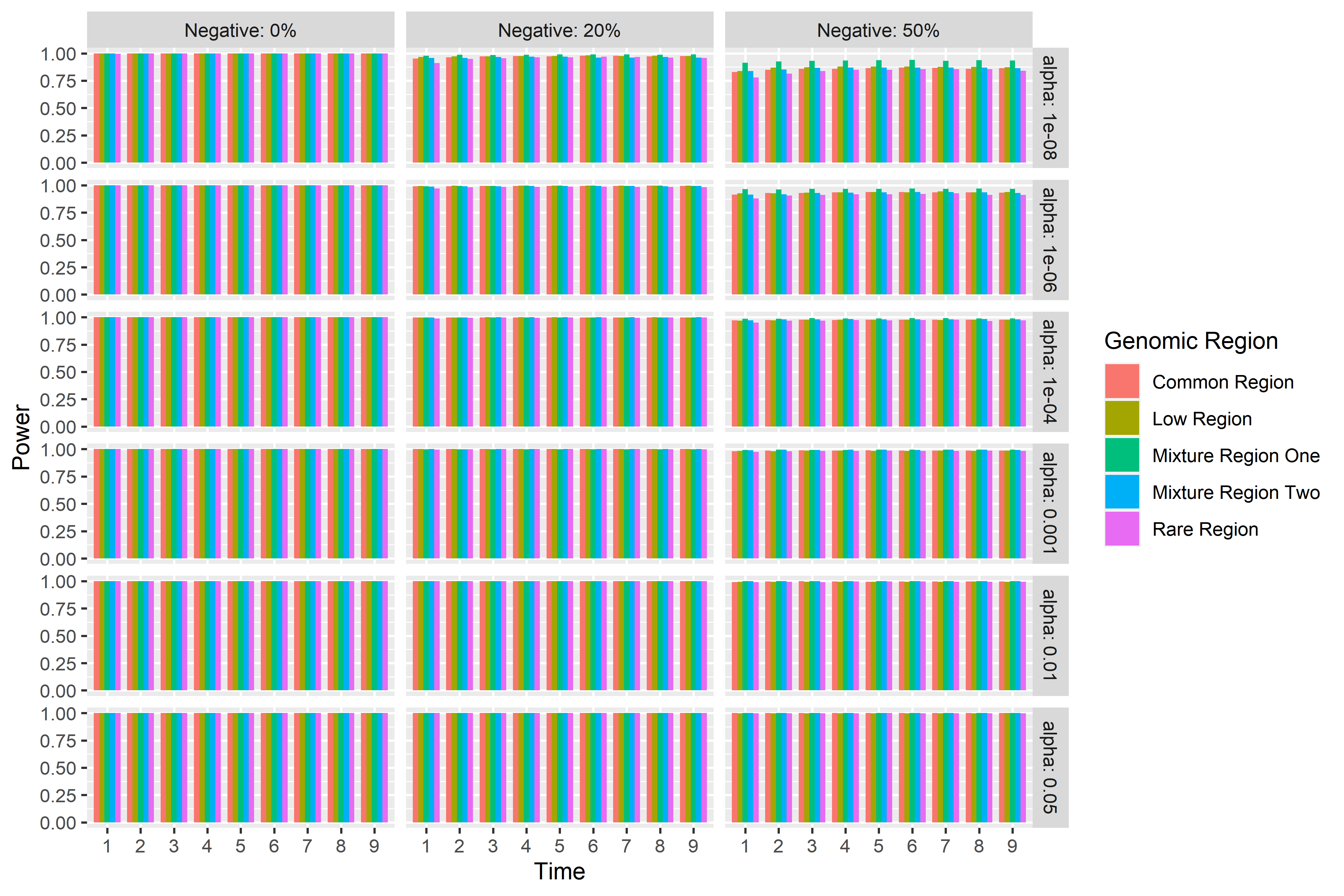

Supplement: Supplementary file 1 [file DataSheet1.ZIP › data in brief/S5/Sample 1500(Case1), c is 7 and the proportion of causal variants is 1%.png]

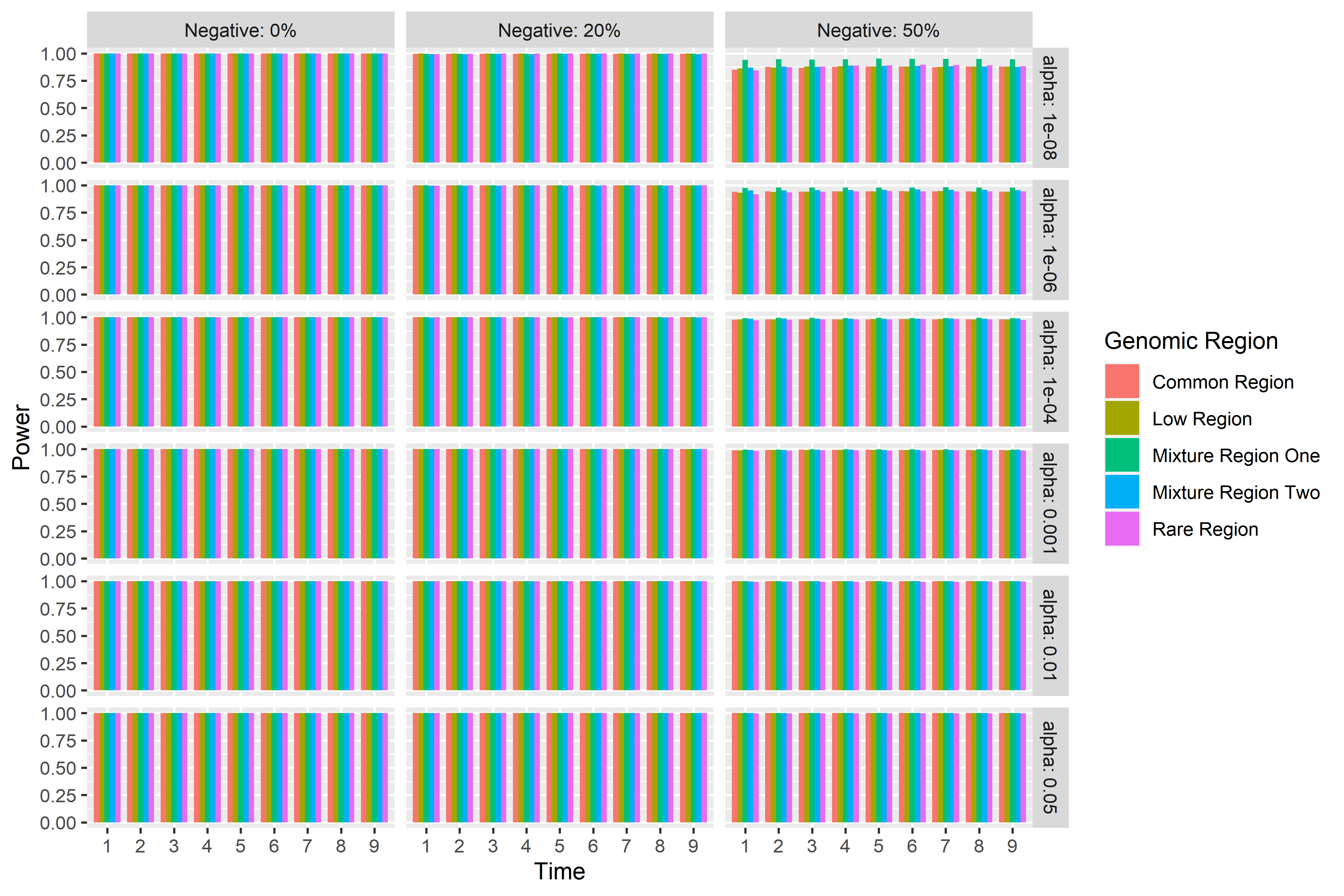

Supplement: Supplementary file 1 [file DataSheet1.ZIP › data in brief/S5/Sample 1500(Case1), c is 7 and the proportion of causal variants is 2%.png]

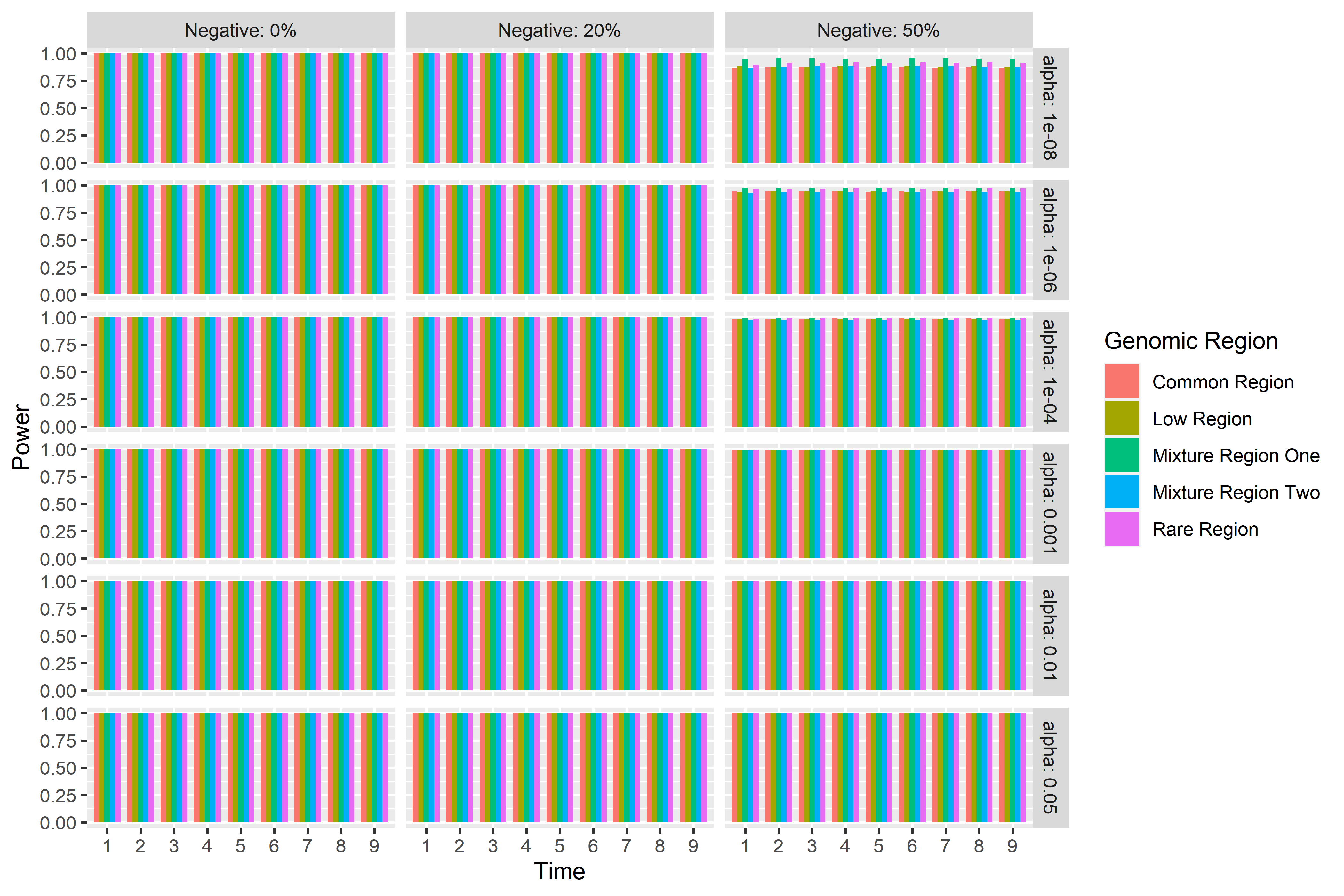

Supplement: Supplementary file 1 [file DataSheet1.ZIP › data in brief/S5/Sample 1500(Case1), c is 7 and the proportion of causal variants is 4%.png]

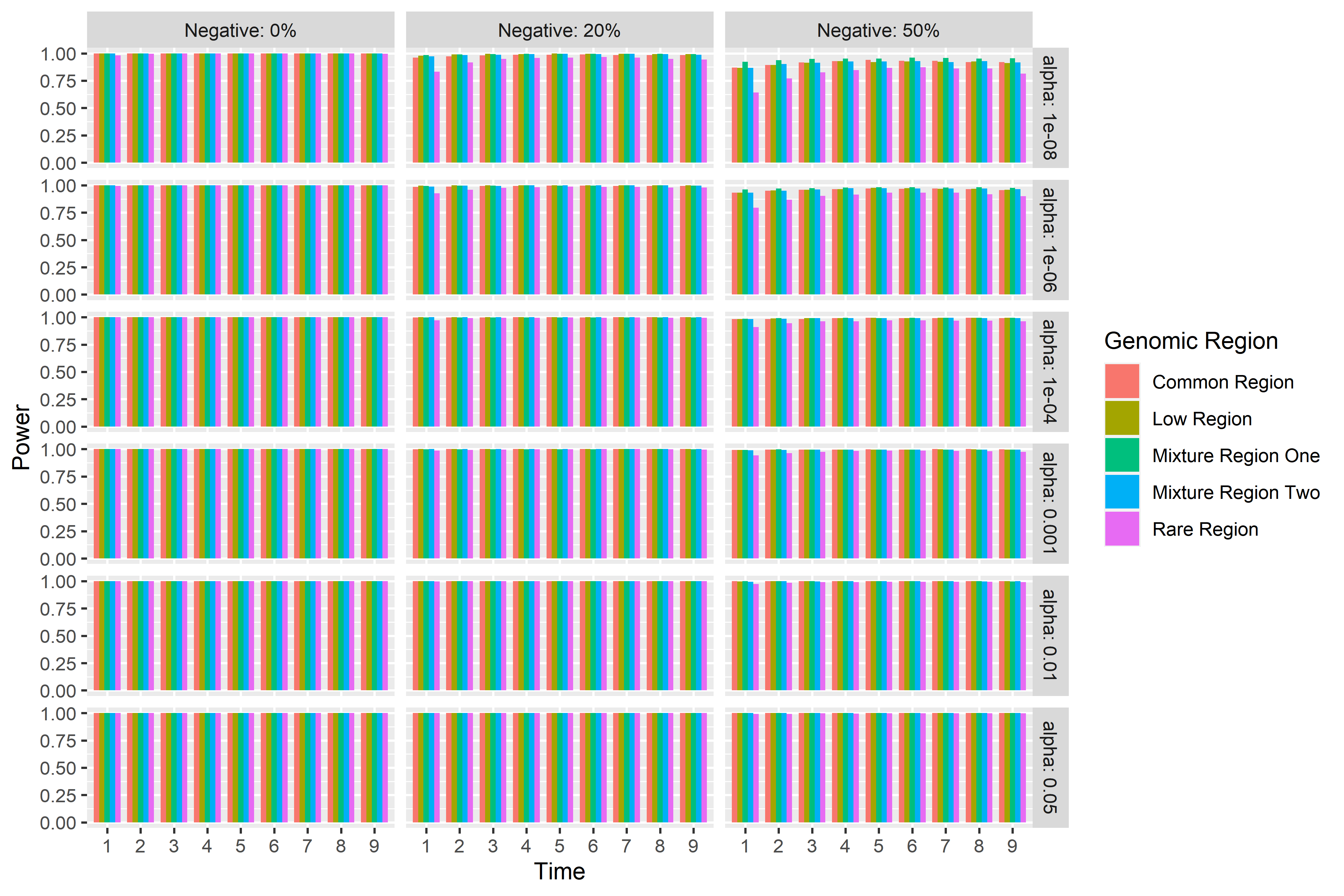

Supplement: Supplementary file 1 [file DataSheet1.ZIP › data in brief/S5/Sample 2000(Case1), c is 3 and the proportion of causal variants is 1%.png]

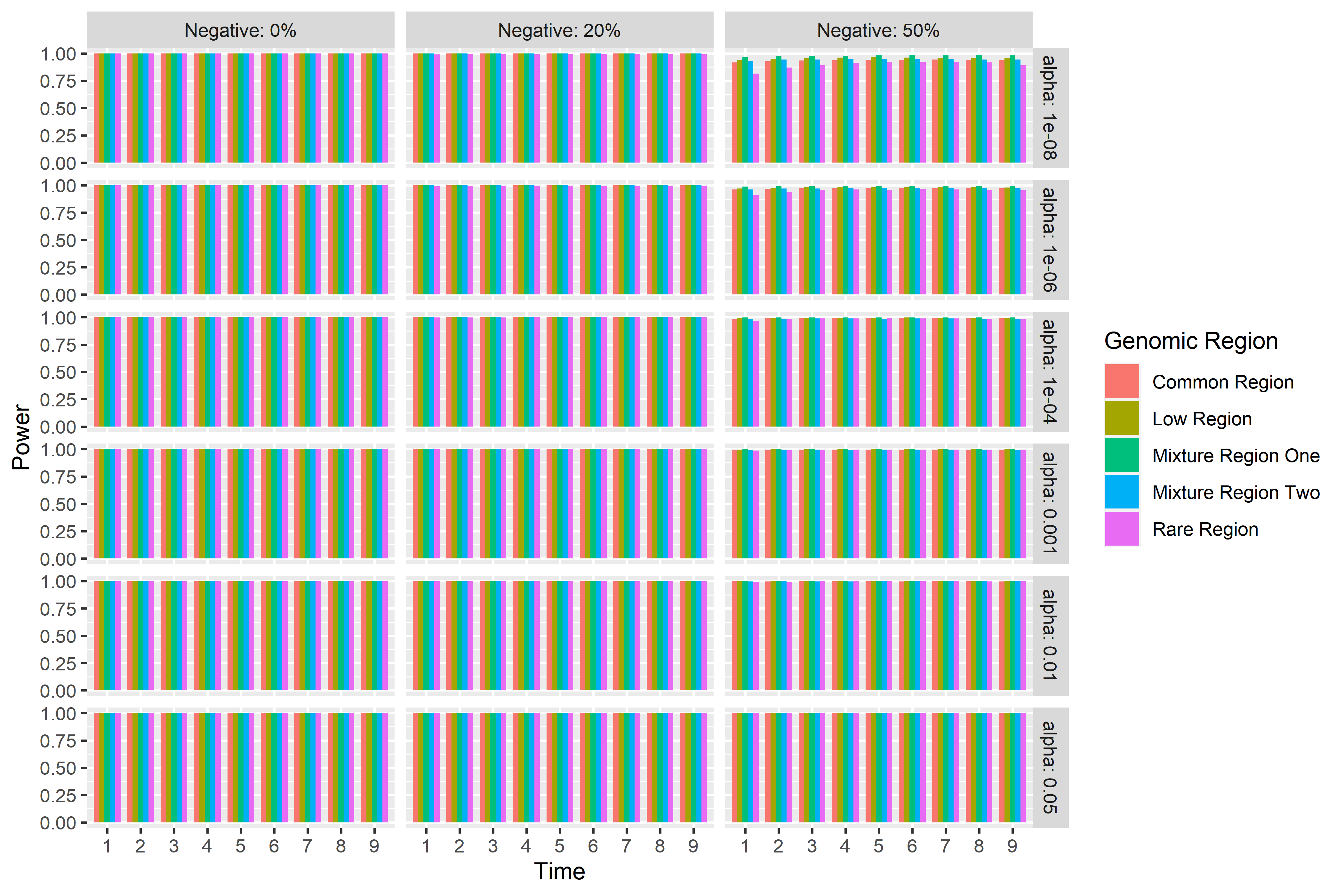

Supplement: Supplementary file 1 [file DataSheet1.ZIP › data in brief/S5/Sample 2000(Case1), c is 3 and the proportion of causal variants is 2%.png]

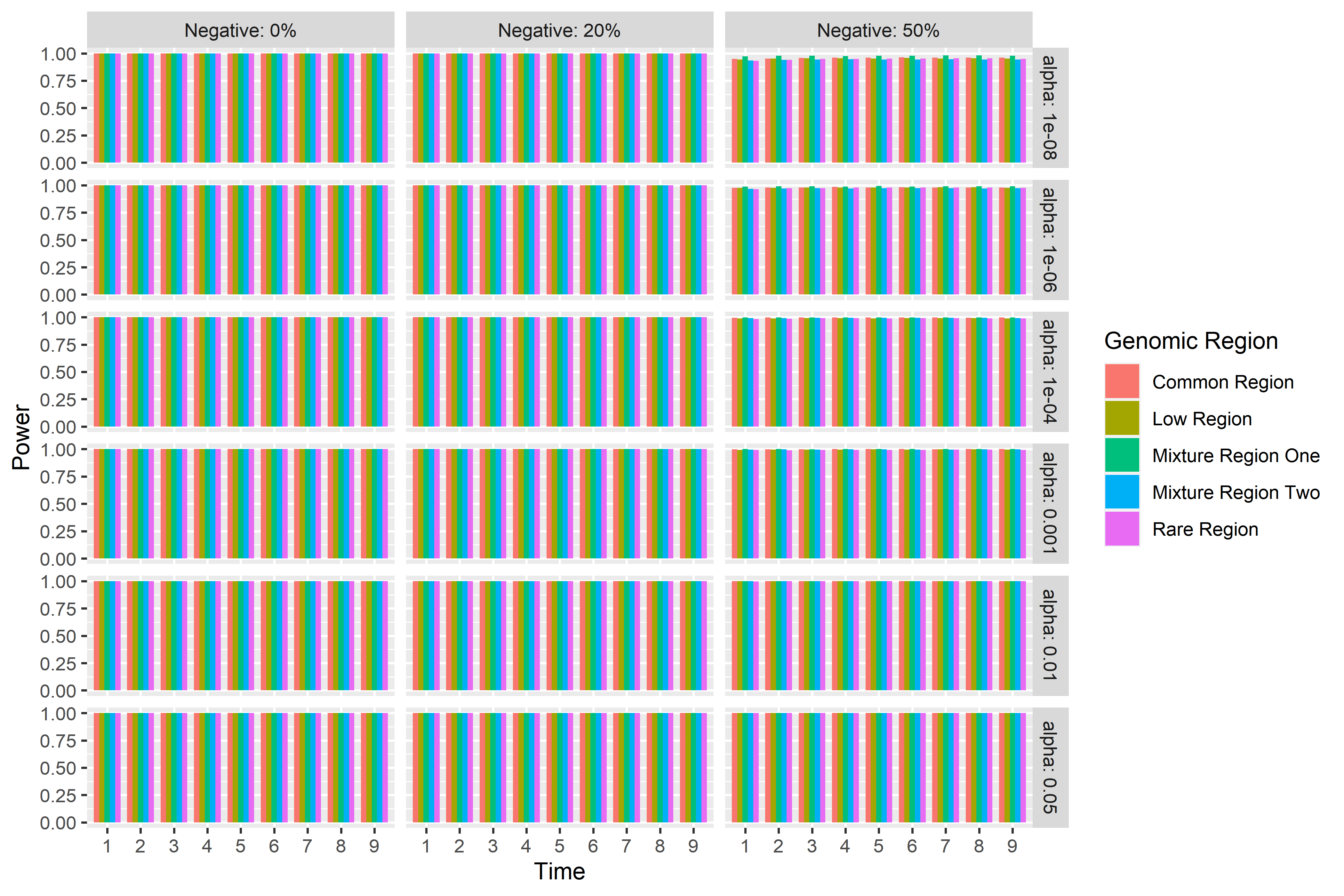

Supplement: Supplementary file 1 [file DataSheet1.ZIP › data in brief/S5/Sample 2000(Case1), c is 3 and the proportion of causal variants is 4%.png]

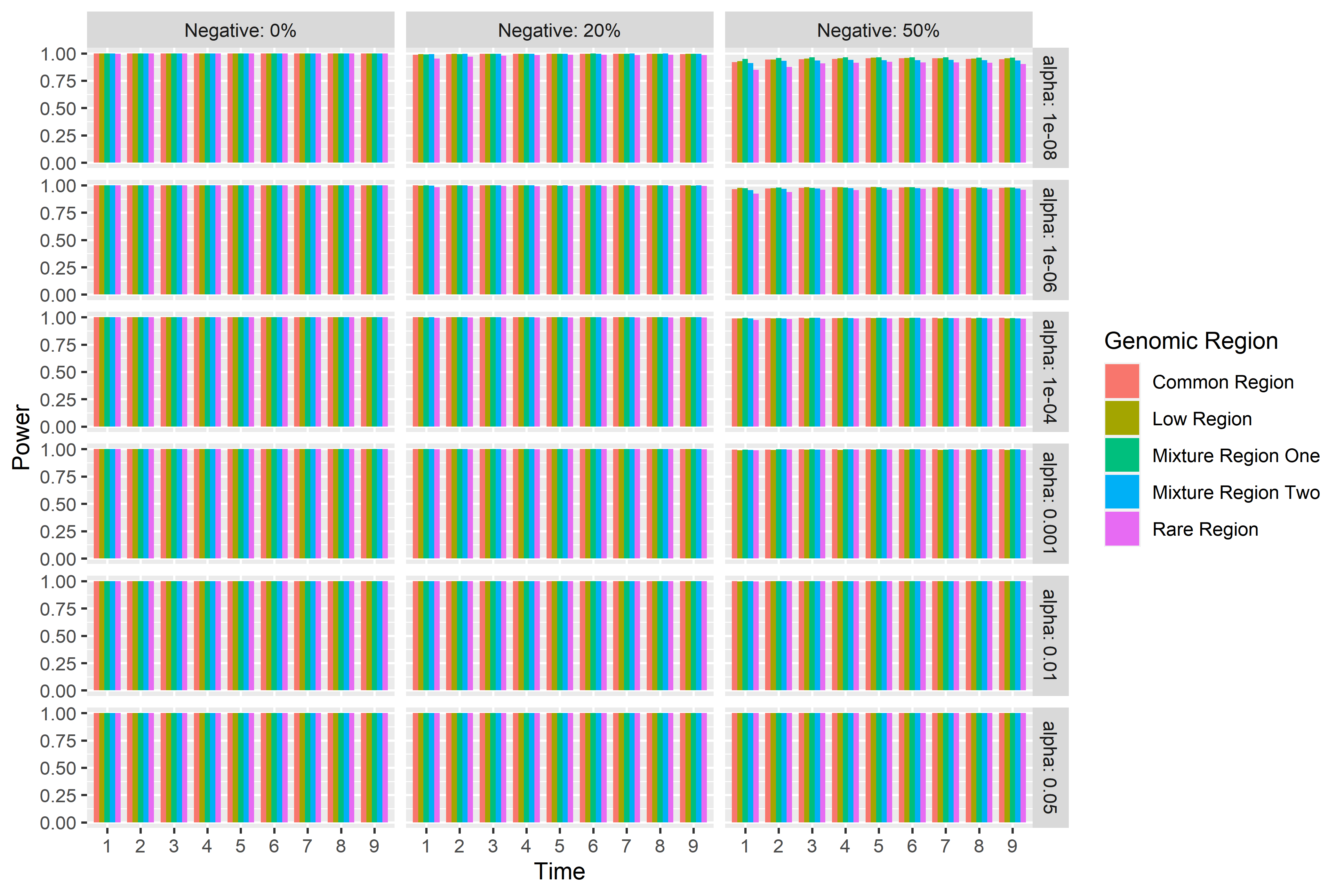

Supplement: Supplementary file 1 [file DataSheet1.ZIP › data in brief/S5/Sample 2000(Case1), c is 5 and the proportion of causal variants is 1%.png]

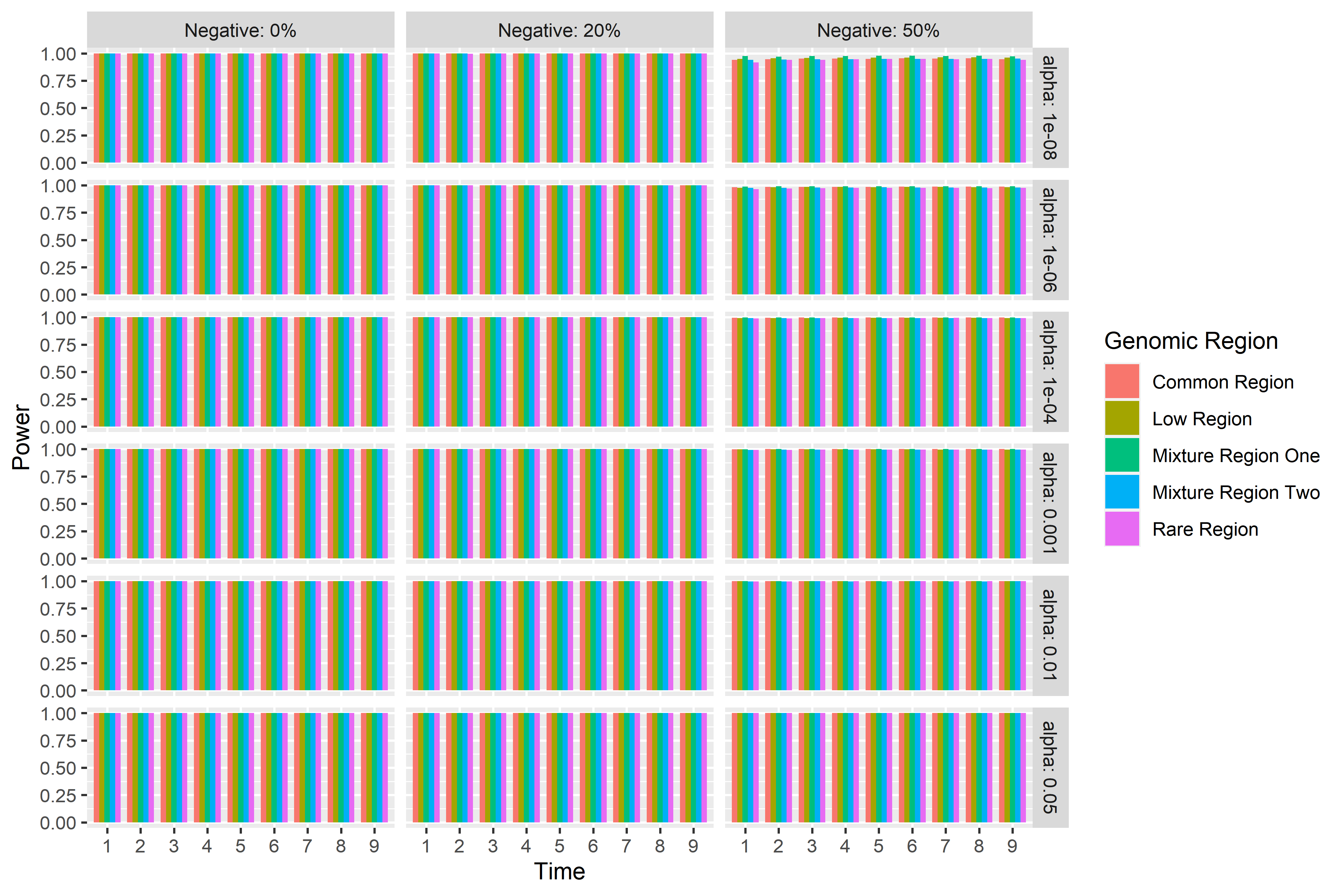

Supplement: Supplementary file 1 [file DataSheet1.ZIP › data in brief/S5/Sample 2000(Case1), c is 5 and the proportion of causal variants is 2%.png]

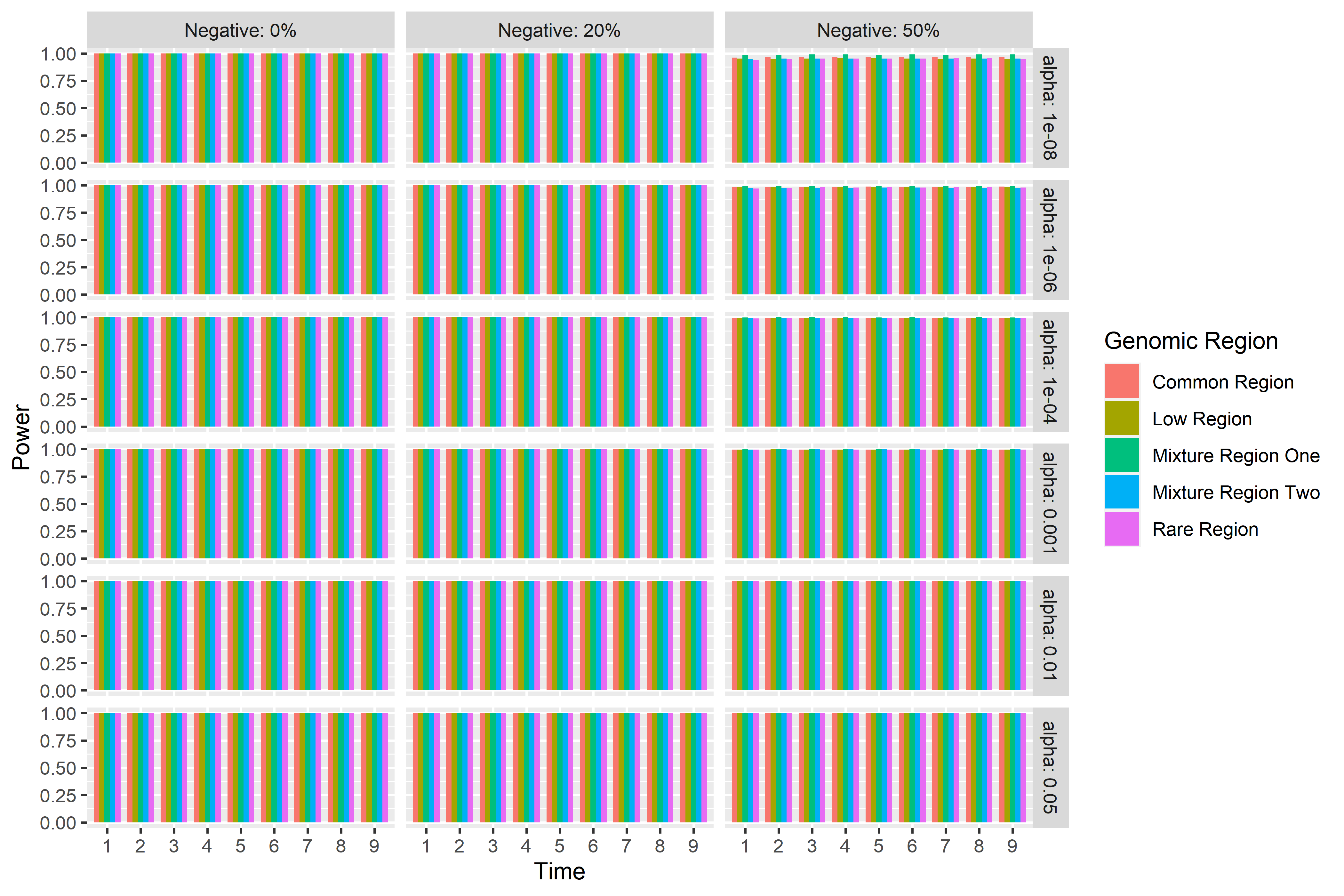

Supplement: Supplementary file 1 [file DataSheet1.ZIP › data in brief/S5/Sample 2000(Case1), c is 5 and the proportion of causal variants is 4%.png]

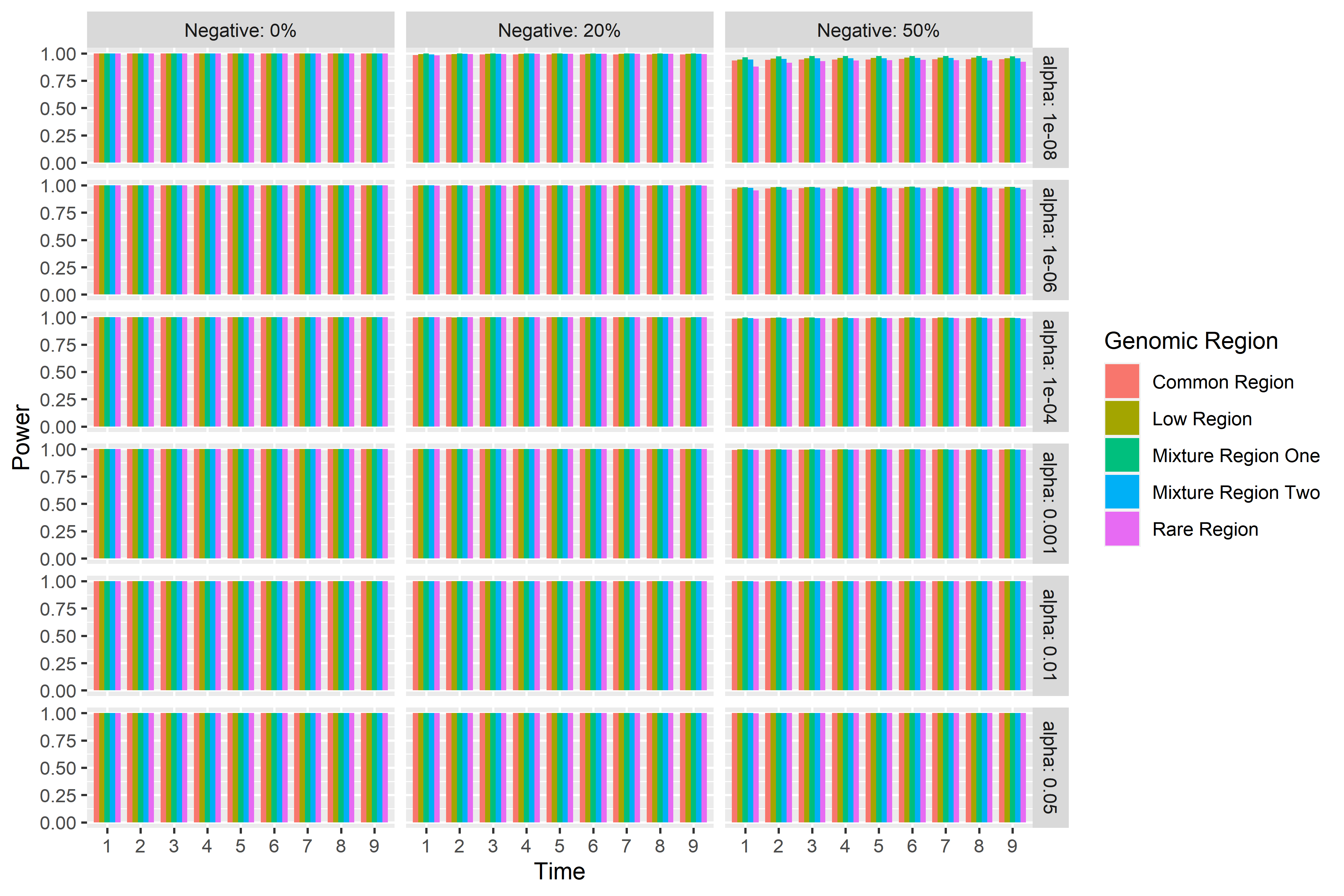

Supplement: Supplementary file 1 [file DataSheet1.ZIP › data in brief/S5/Sample 2000(Case1), c is 7 and the proportion of causal variants is 1%.png]

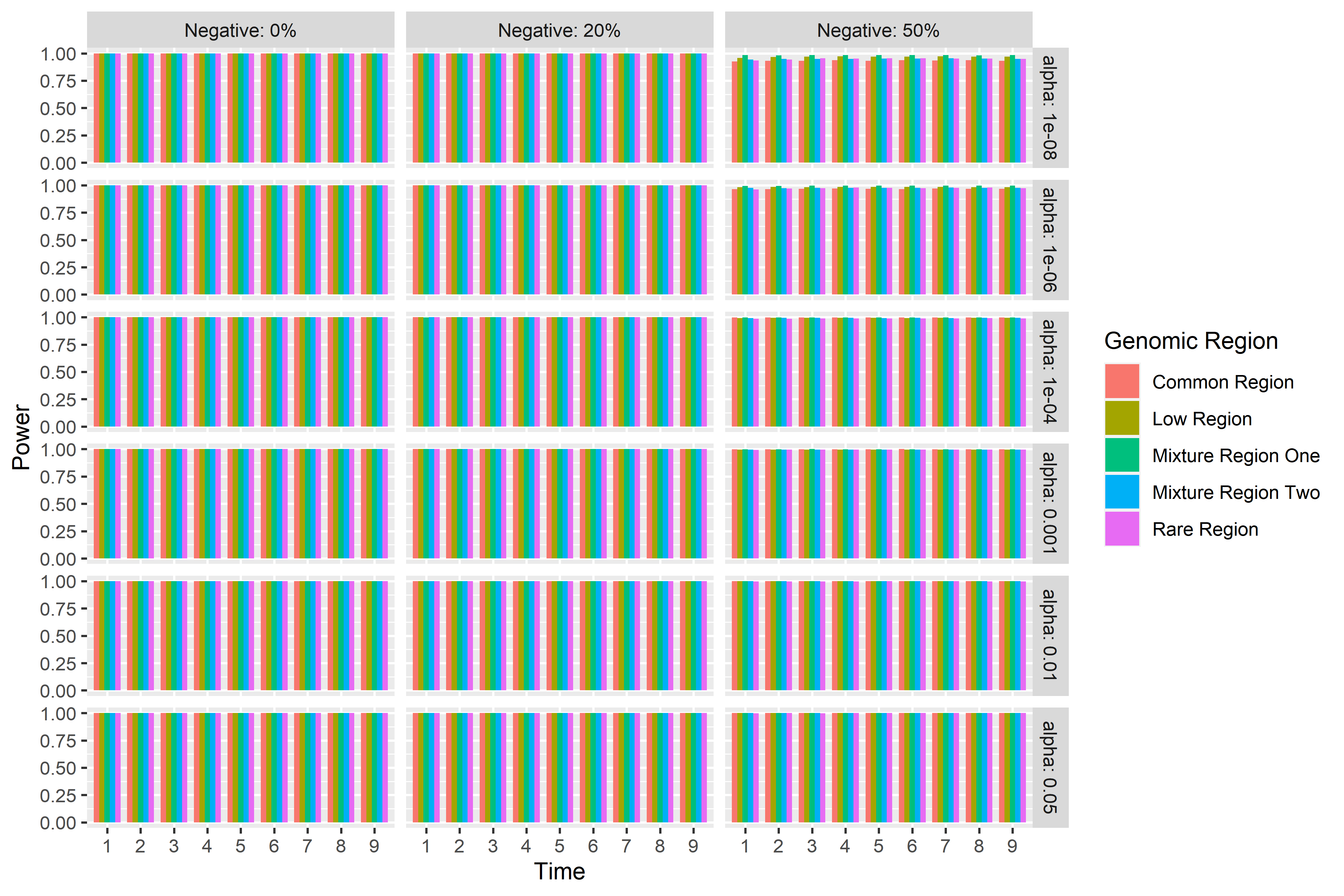

Supplement: Supplementary file 1 [file DataSheet1.ZIP › data in brief/S5/Sample 2000(Case1), c is 7 and the proportion of causal variants is 2%.png]

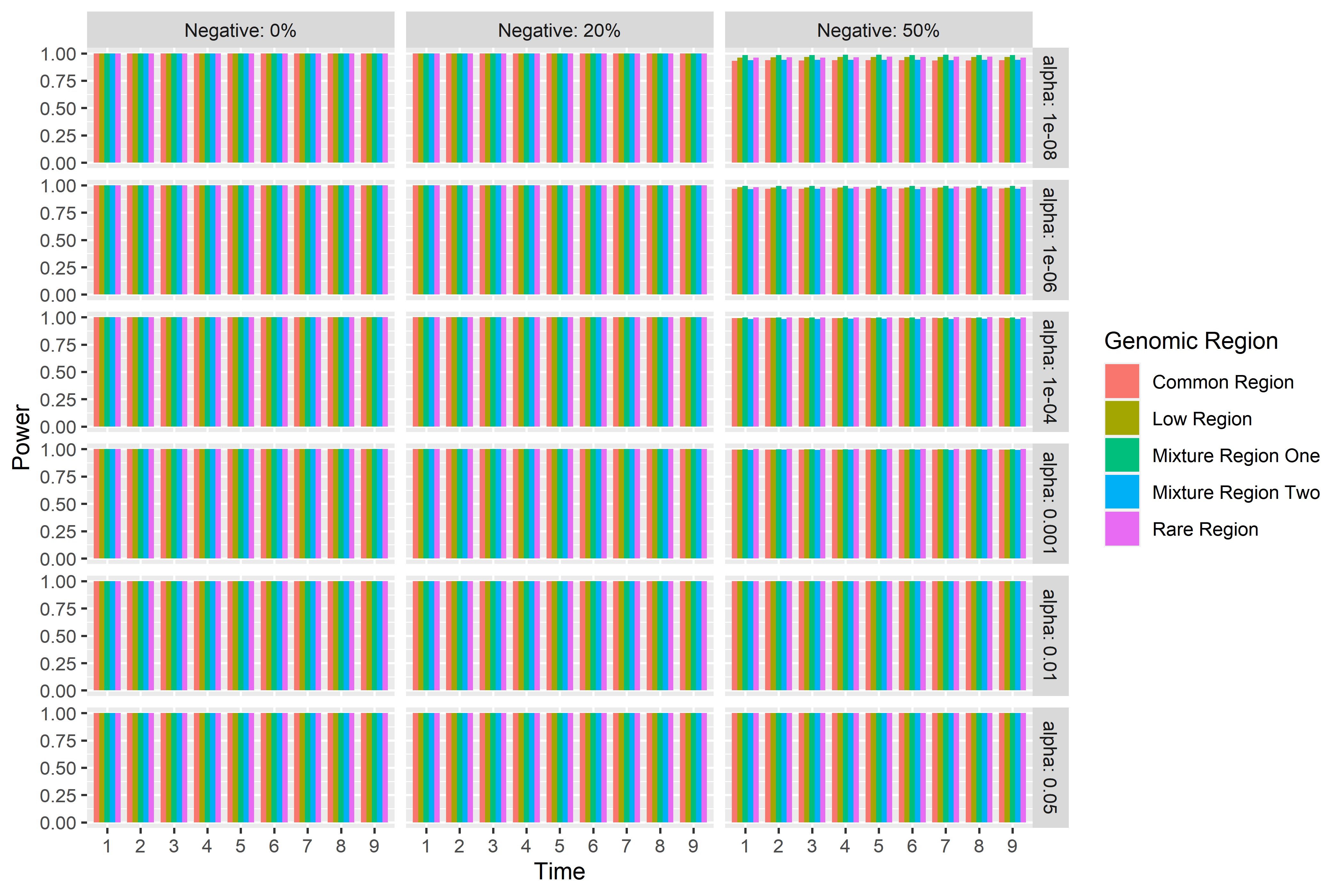

Supplement: Supplementary file 1 [file DataSheet1.ZIP › data in brief/S5/Sample 2000(Case1), c is 7 and the proportion of causal variants is 4%.png]

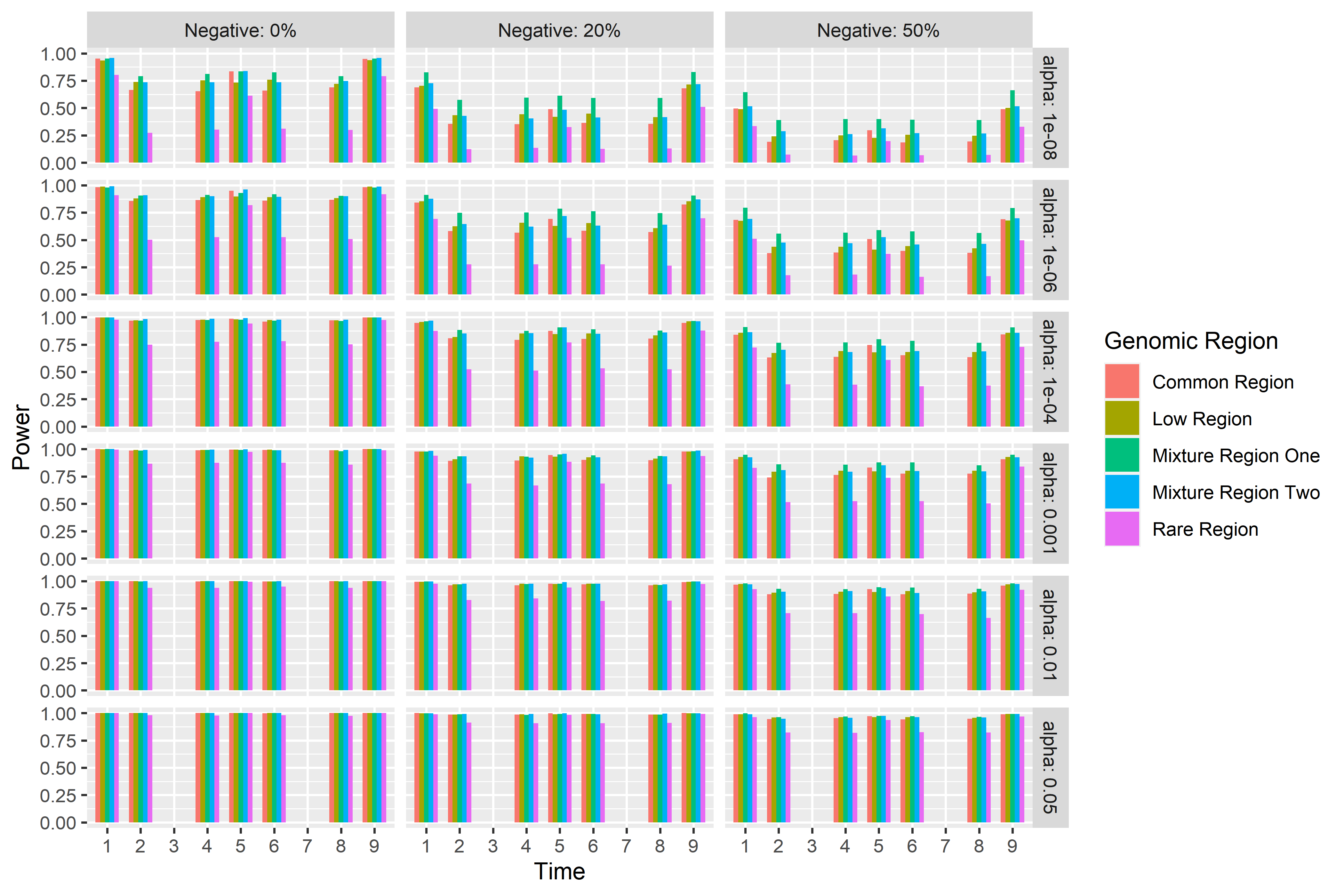

Supplement: Supplementary file 1 [file DataSheet1.ZIP › data in brief/S6/Sample 1000(Case2), c is 3 and the proportion of causal variants is 1%.png]

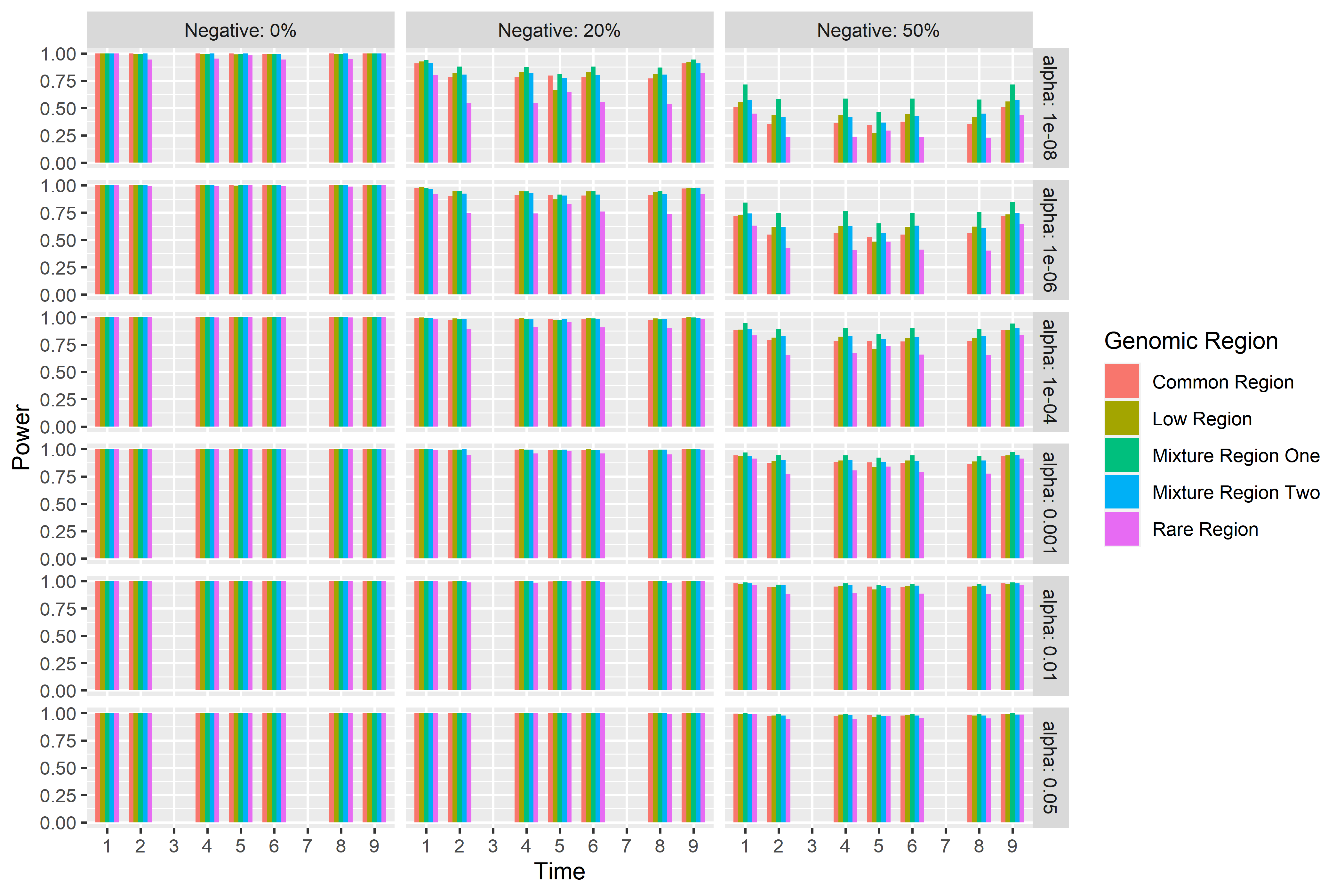

Supplement: Supplementary file 1 [file DataSheet1.ZIP › data in brief/S6/Sample 1000(Case2), c is 3 and the proportion of causal variants is 2%.png]

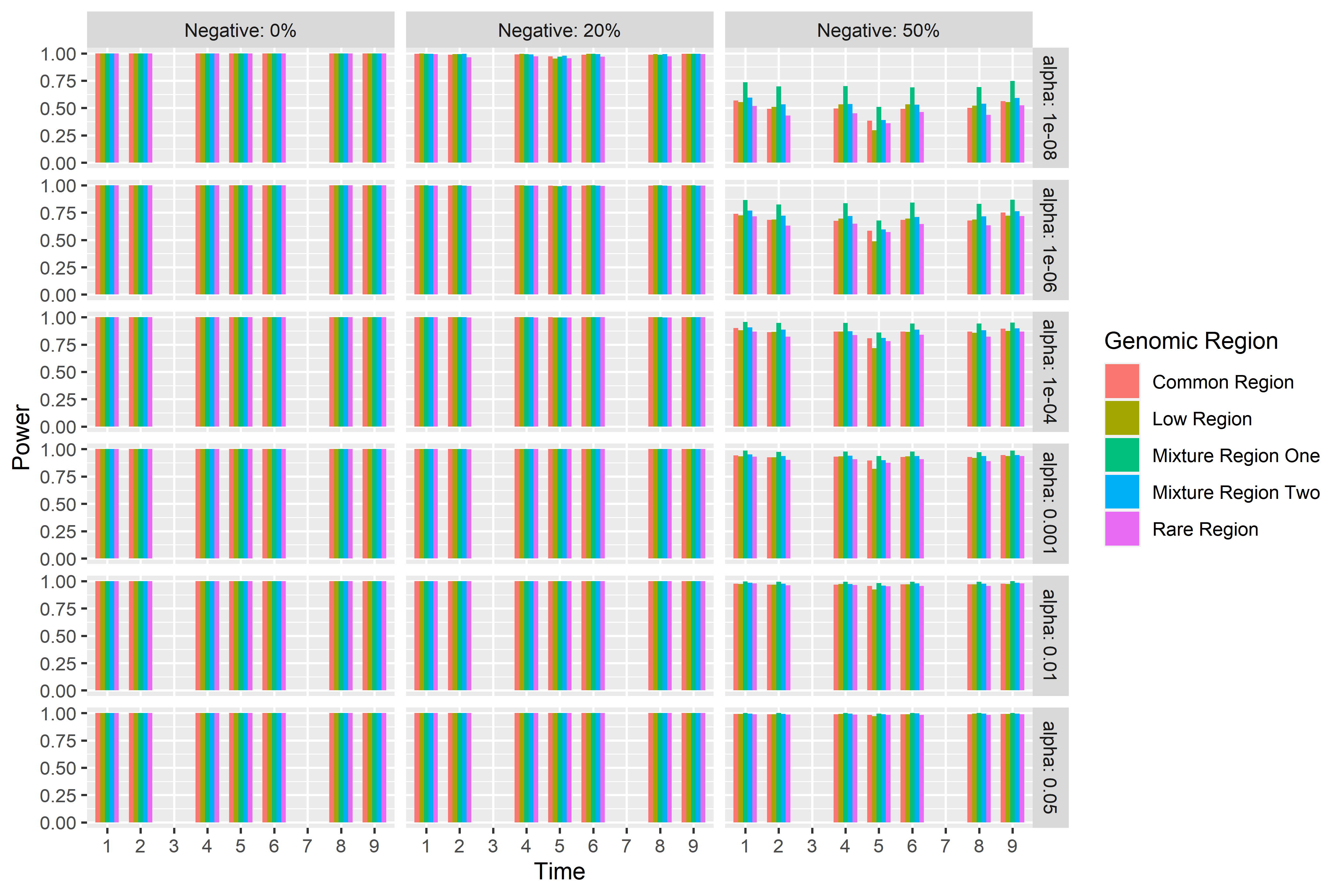

Supplement: Supplementary file 1 [file DataSheet1.ZIP › data in brief/S6/Sample 1000(Case2), c is 3 and the proportion of causal variants is 4%.png]

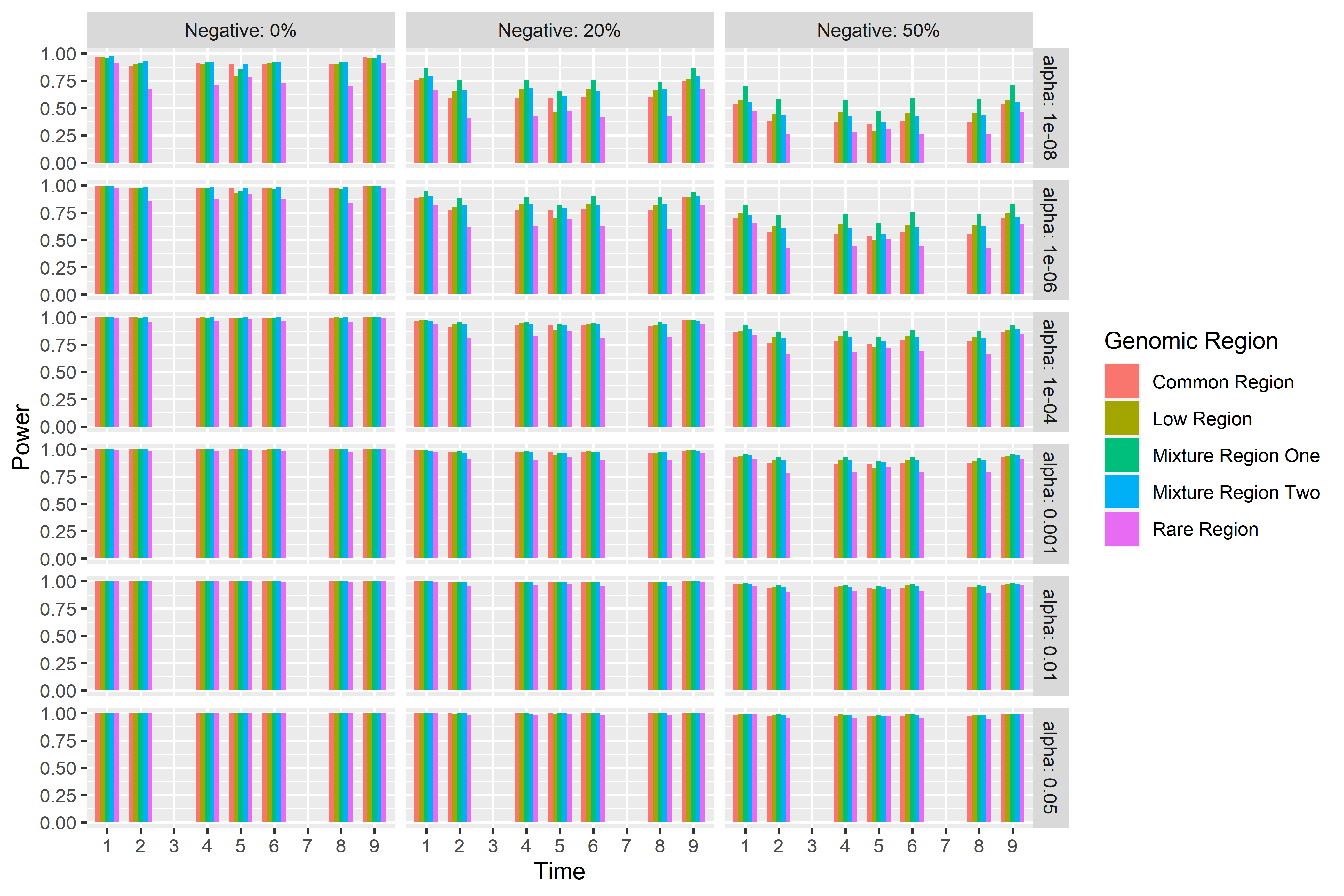

Supplement: Supplementary file 1 [file DataSheet1.ZIP › data in brief/S6/Sample 1000(Case2), c is 5 and the proportion of causal variants is 1%.png]

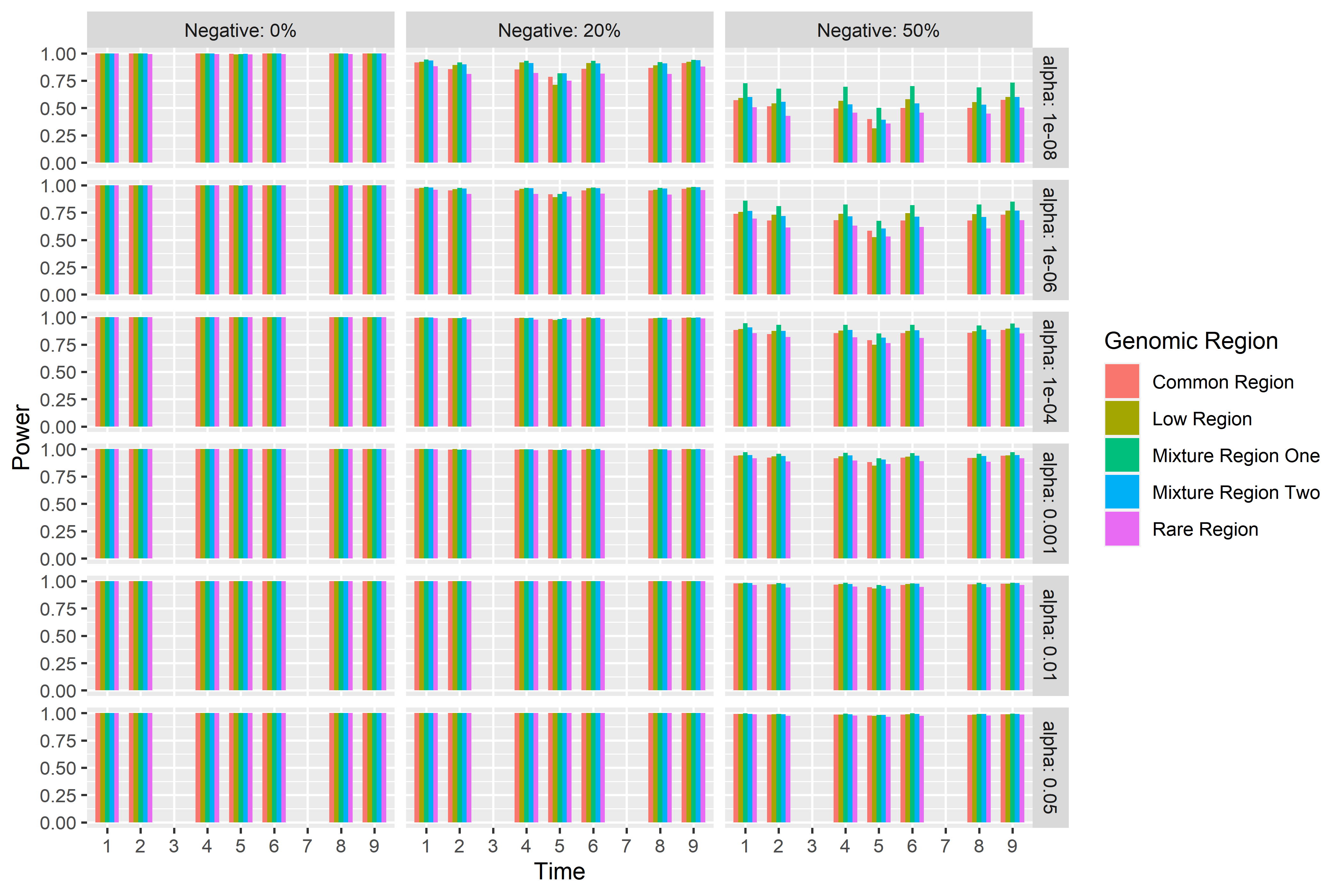

Supplement: Supplementary file 1 [file DataSheet1.ZIP › data in brief/S6/Sample 1000(Case2), c is 5 and the proportion of causal variants is 2%.png]

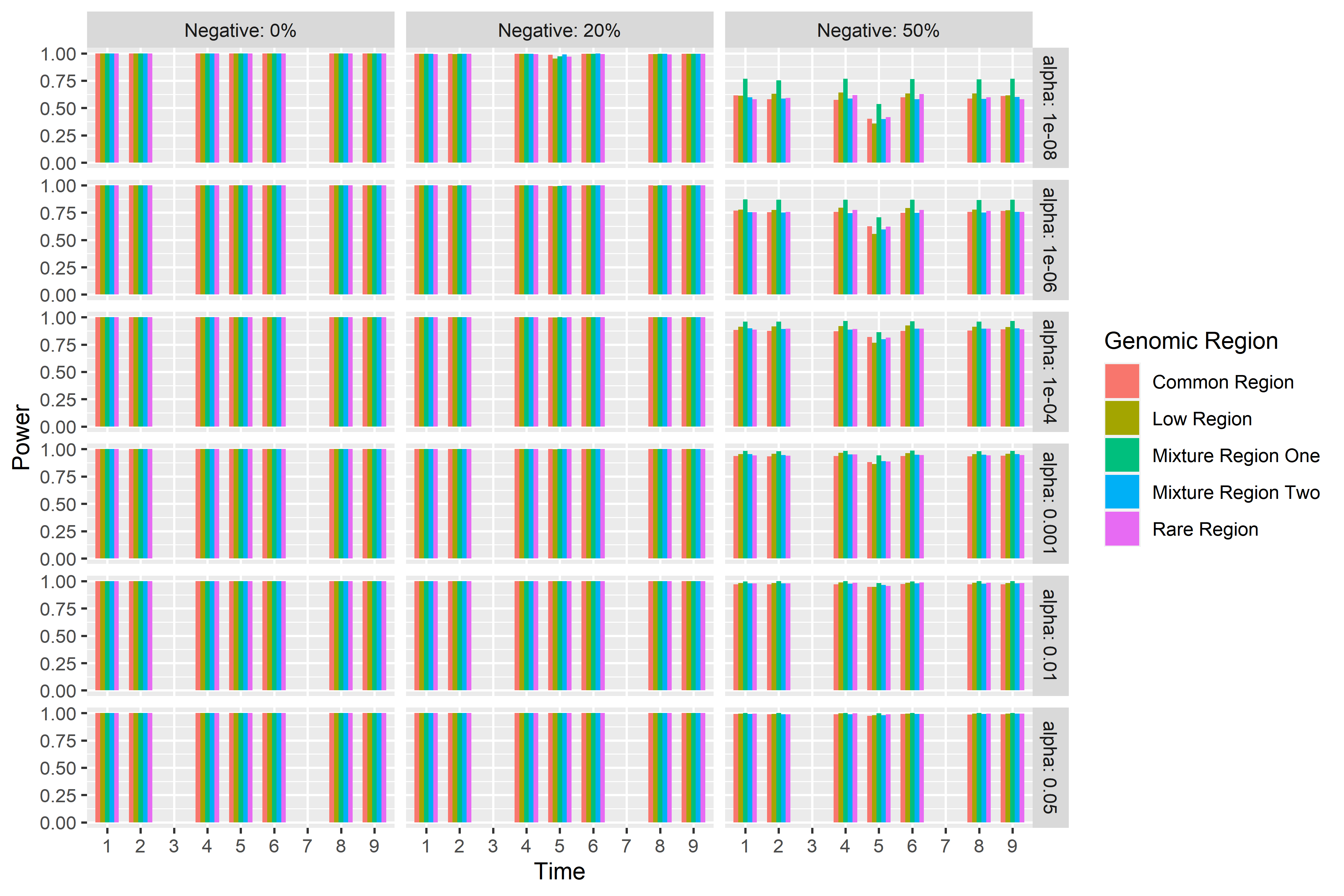

Supplement: Supplementary file 1 [file DataSheet1.ZIP › data in brief/S6/Sample 1000(Case2), c is 5 and the proportion of causal variants is 4%.png]

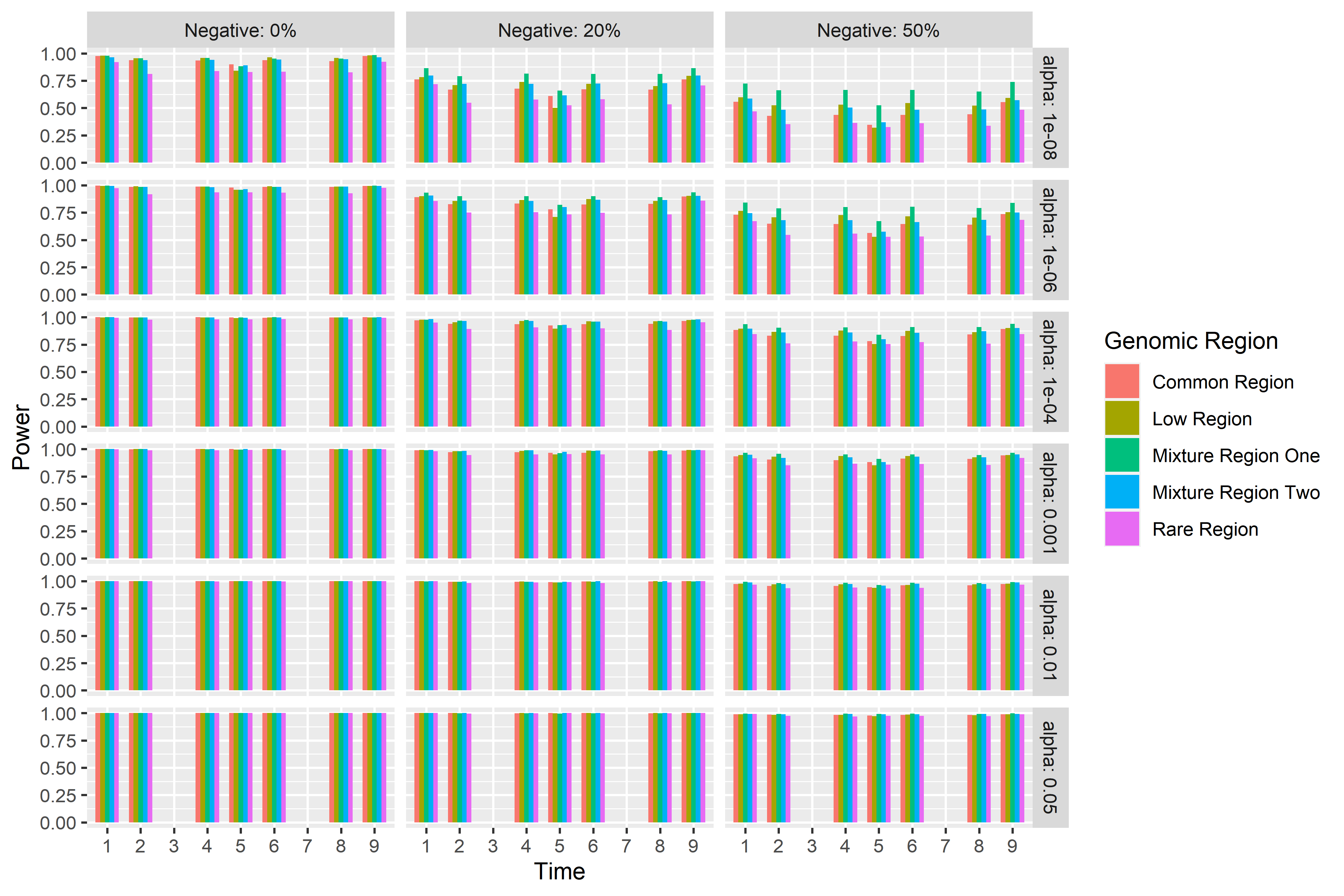

Supplement: Supplementary file 1 [file DataSheet1.ZIP › data in brief/S6/Sample 1000(Case2), c is 7 and the proportion of causal variants is 1%.png]

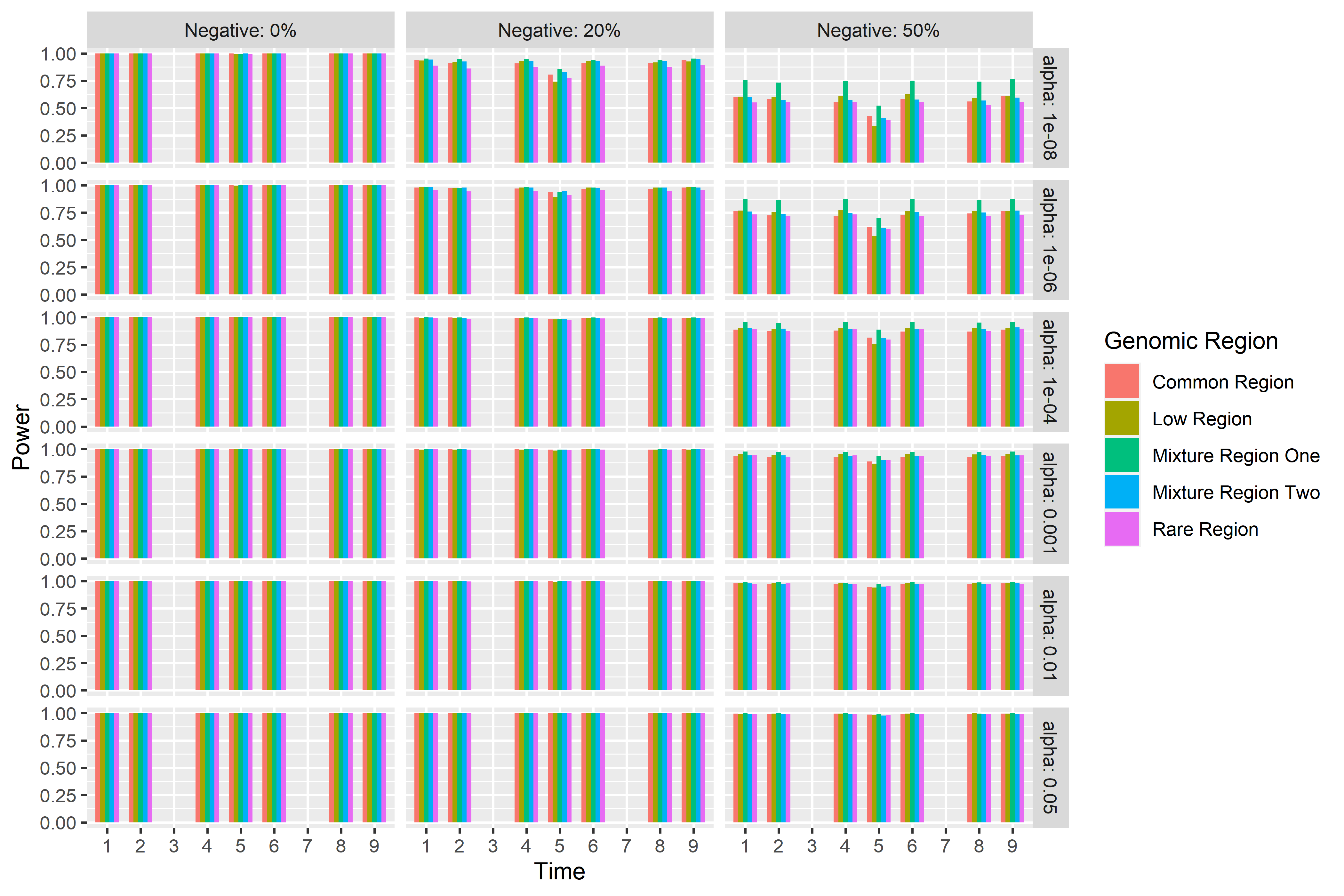

Supplement: Supplementary file 1 [file DataSheet1.ZIP › data in brief/S6/Sample 1000(Case2), c is 7 and the proportion of causal variants is 2%.png]

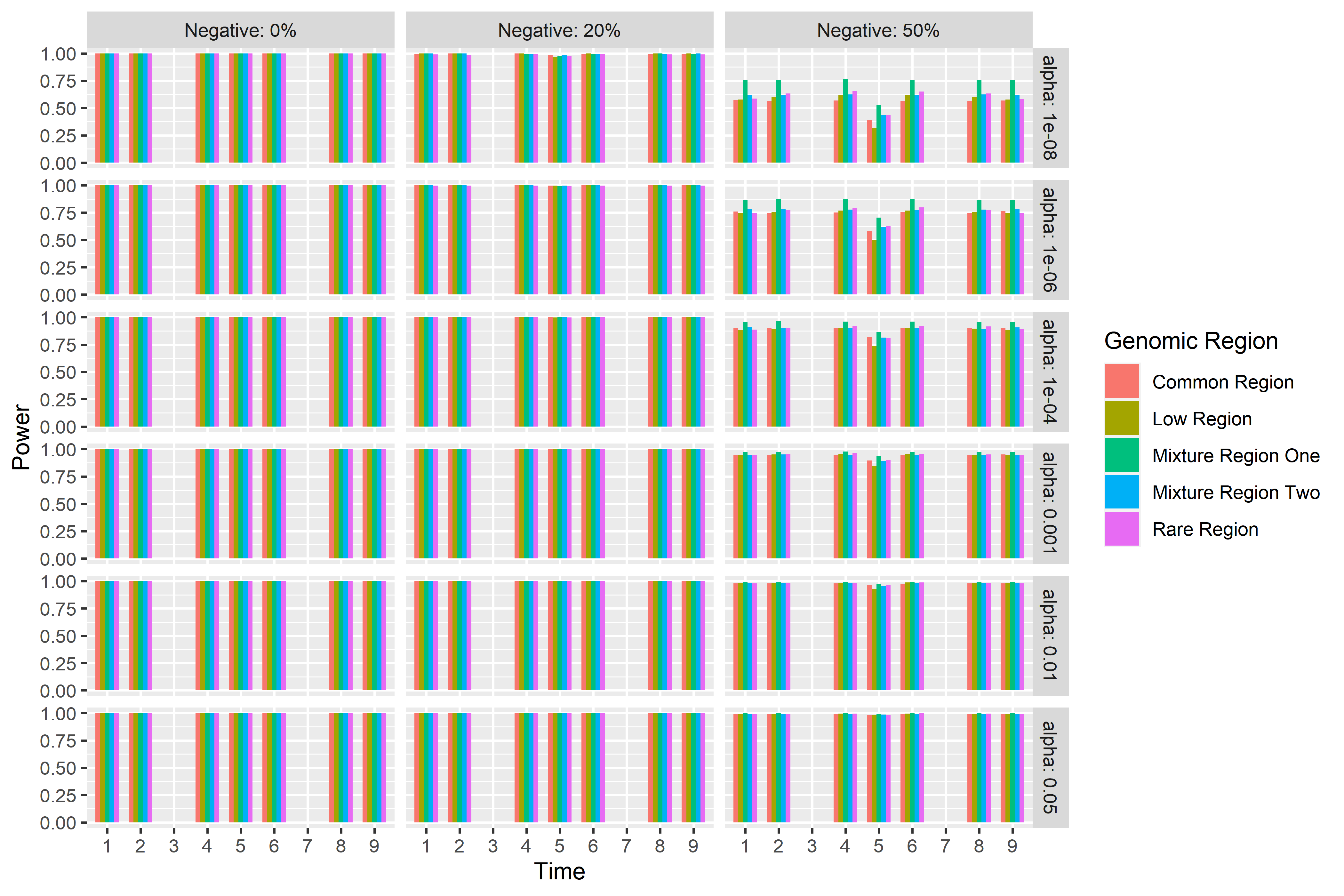

Supplement: Supplementary file 1 [file DataSheet1.ZIP › data in brief/S6/Sample 1000(Case2), c is 7 and the proportion of causal variants is 4%.png]

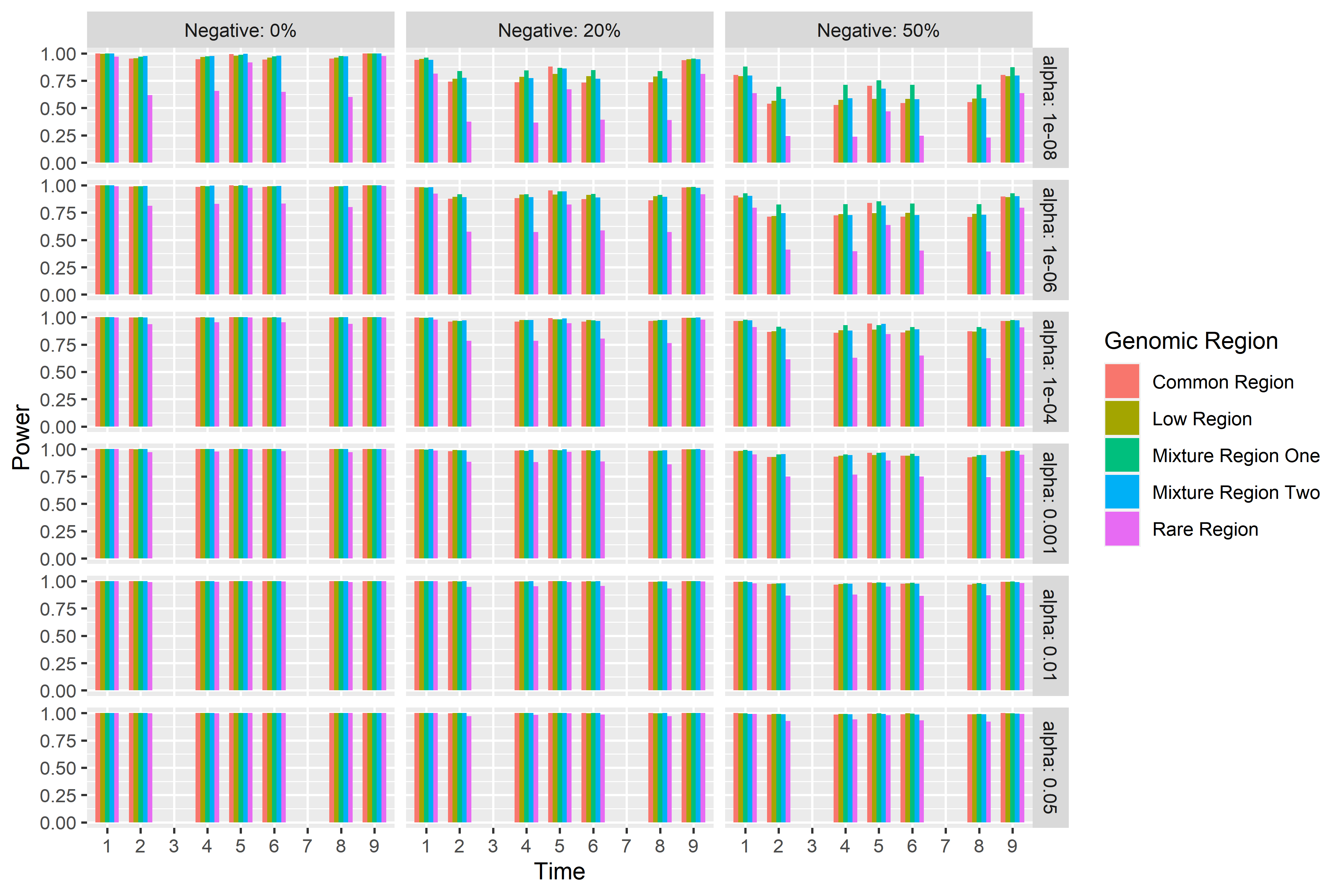

Supplement: Supplementary file 1 [file DataSheet1.ZIP › data in brief/S6/Sample 1500(Case2), c is 3 and the proportion of causal variants is 1%.png]

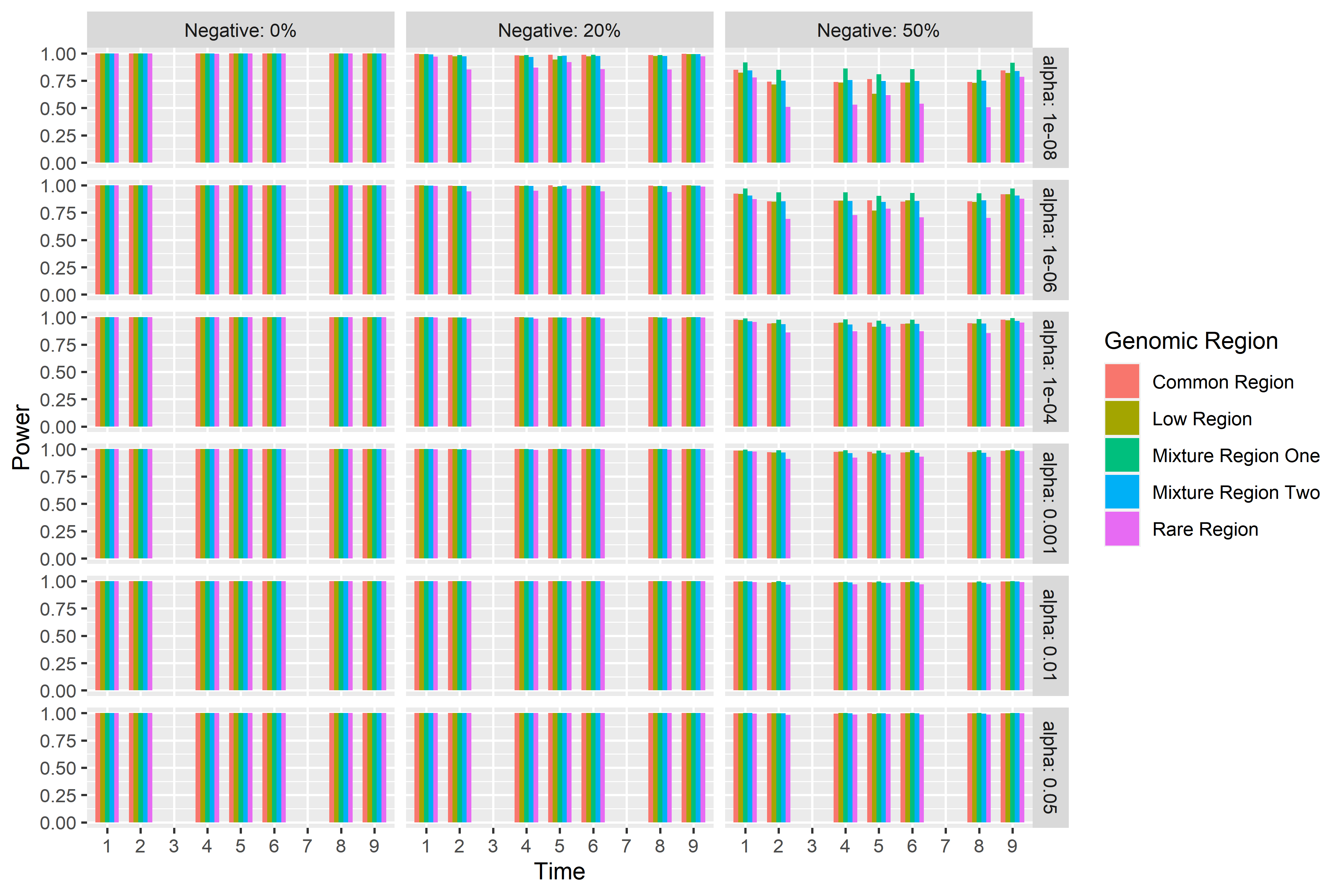

Supplement: Supplementary file 1 [file DataSheet1.ZIP › data in brief/S6/Sample 1500(Case2), c is 3 and the proportion of causal variants is 2%.png]

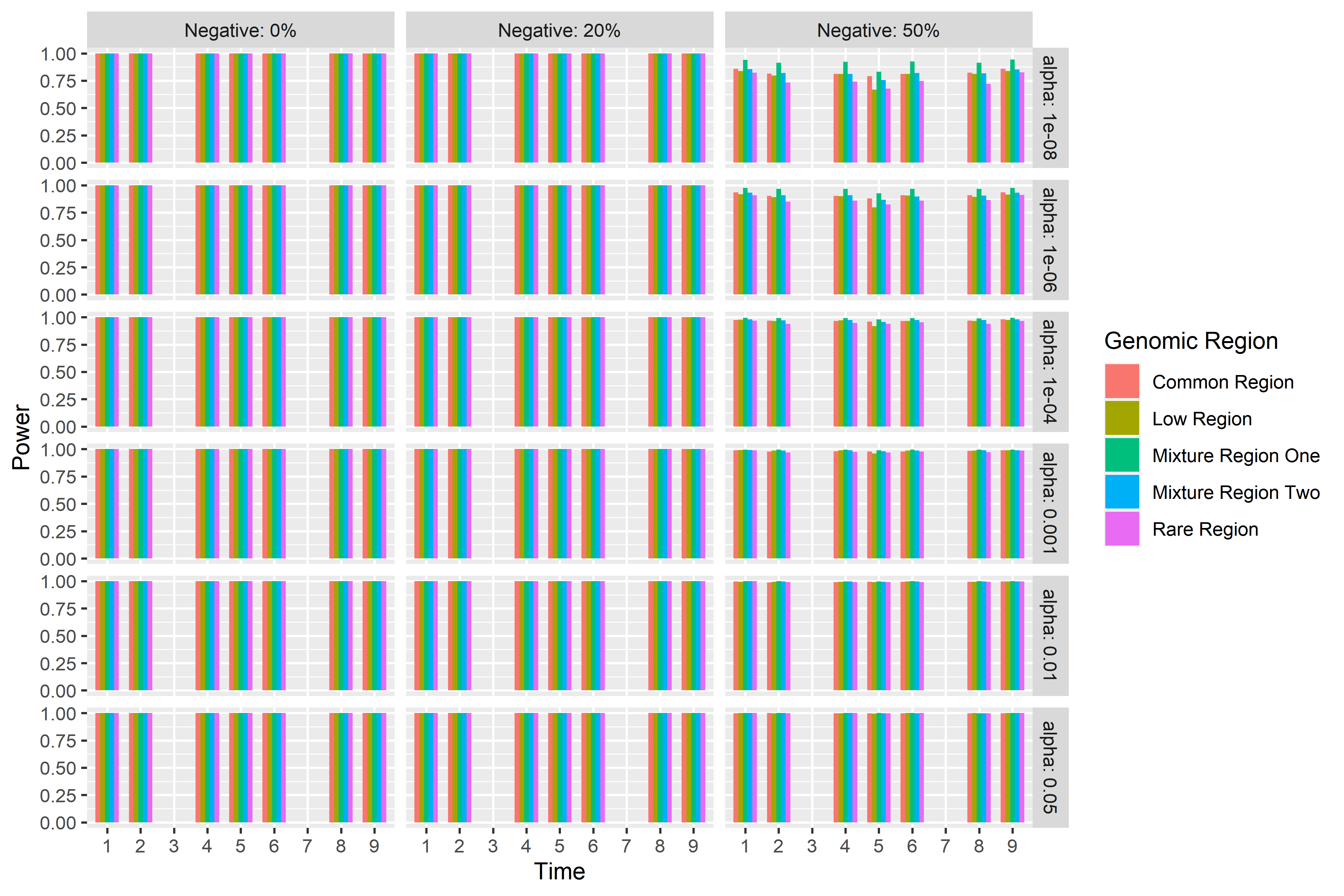

Supplement: Supplementary file 1 [file DataSheet1.ZIP › data in brief/S6/Sample 1500(Case2), c is 3 and the proportion of causal variants is 4%.png]

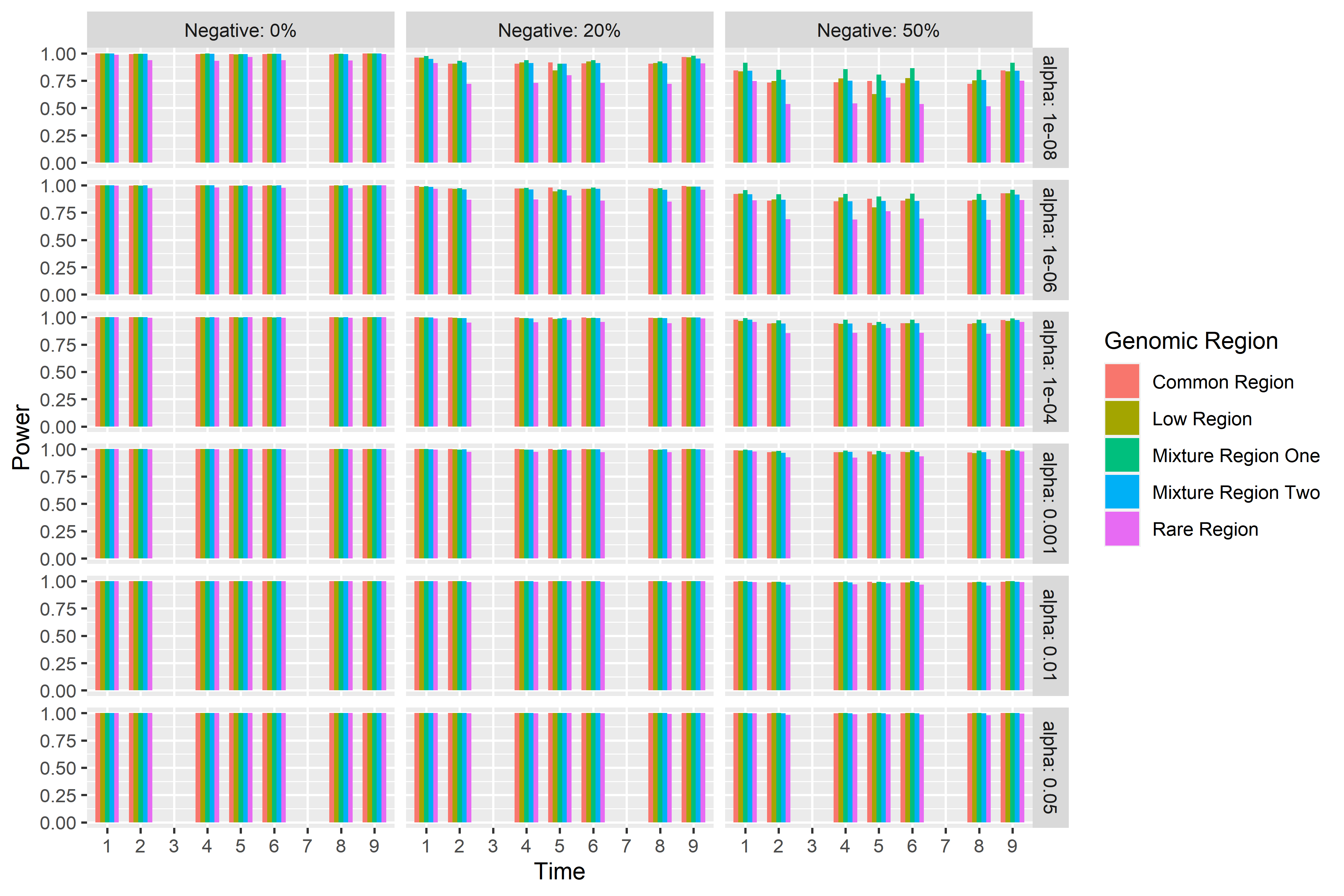

Supplement: Supplementary file 1 [file DataSheet1.ZIP › data in brief/S6/Sample 1500(Case2), c is 5 and the proportion of causal variants is 1%.png]

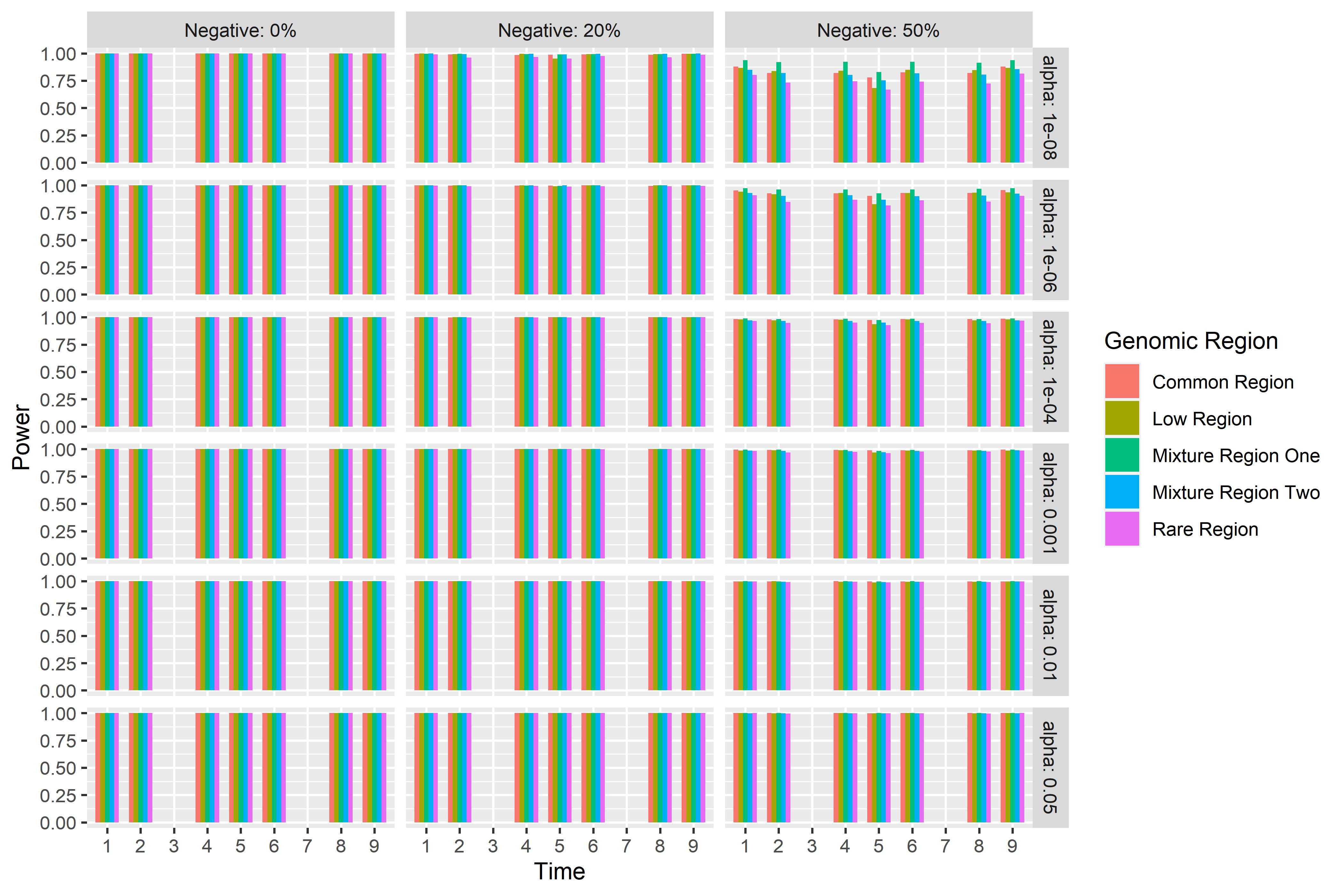

Supplement: Supplementary file 1 [file DataSheet1.ZIP › data in brief/S6/Sample 1500(Case2), c is 5 and the proportion of causal variants is 2%.png]

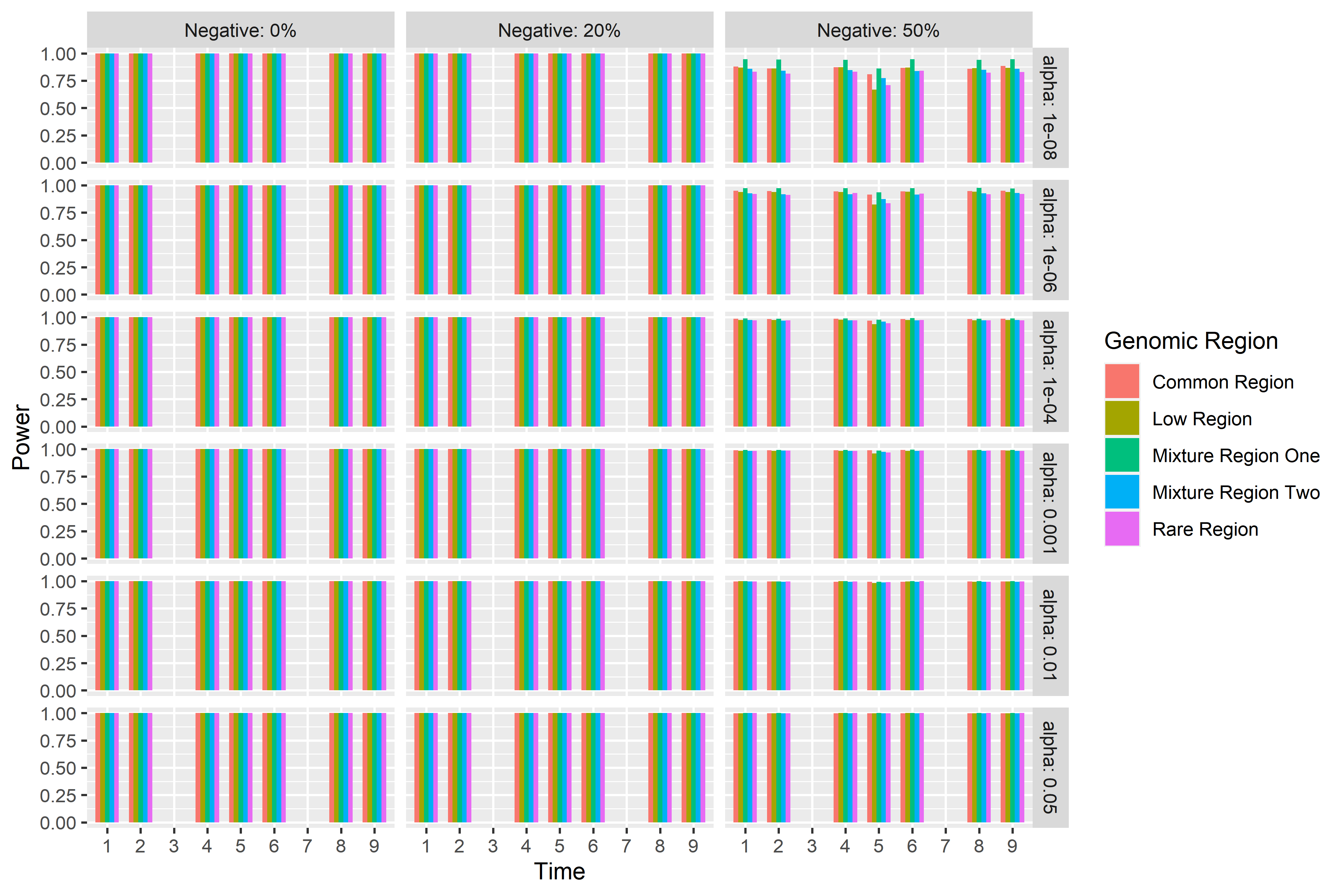

Supplement: Supplementary file 1 [file DataSheet1.ZIP › data in brief/S6/Sample 1500(Case2), c is 5 and the proportion of causal variants is 4%.png]

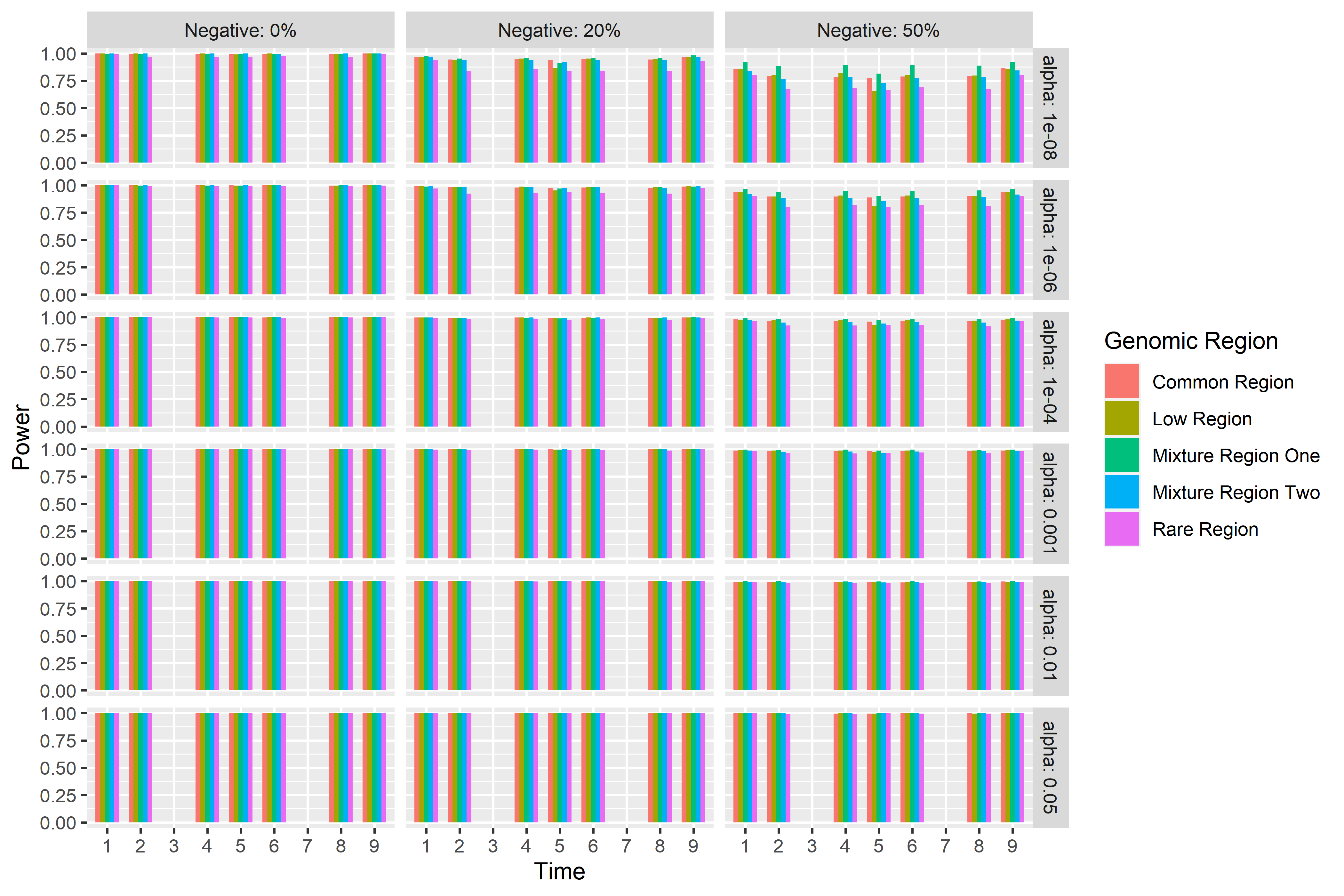

Supplement: Supplementary file 1 [file DataSheet1.ZIP › data in brief/S6/Sample 1500(Case2), c is 7 and the proportion of causal variants is 1%.png]

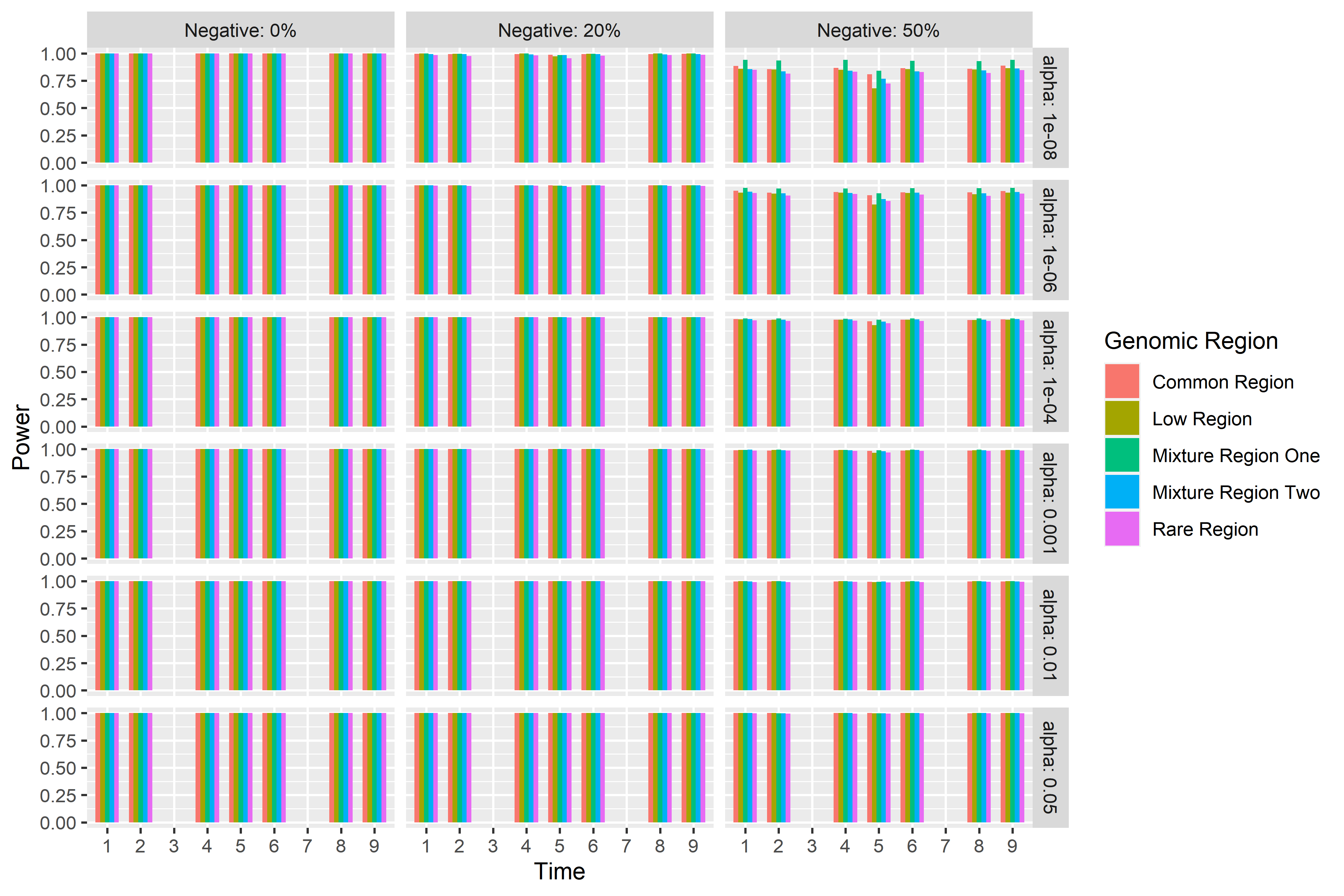

Supplement: Supplementary file 1 [file DataSheet1.ZIP › data in brief/S6/Sample 1500(Case2), c is 7 and the proportion of causal variants is 2%.png]

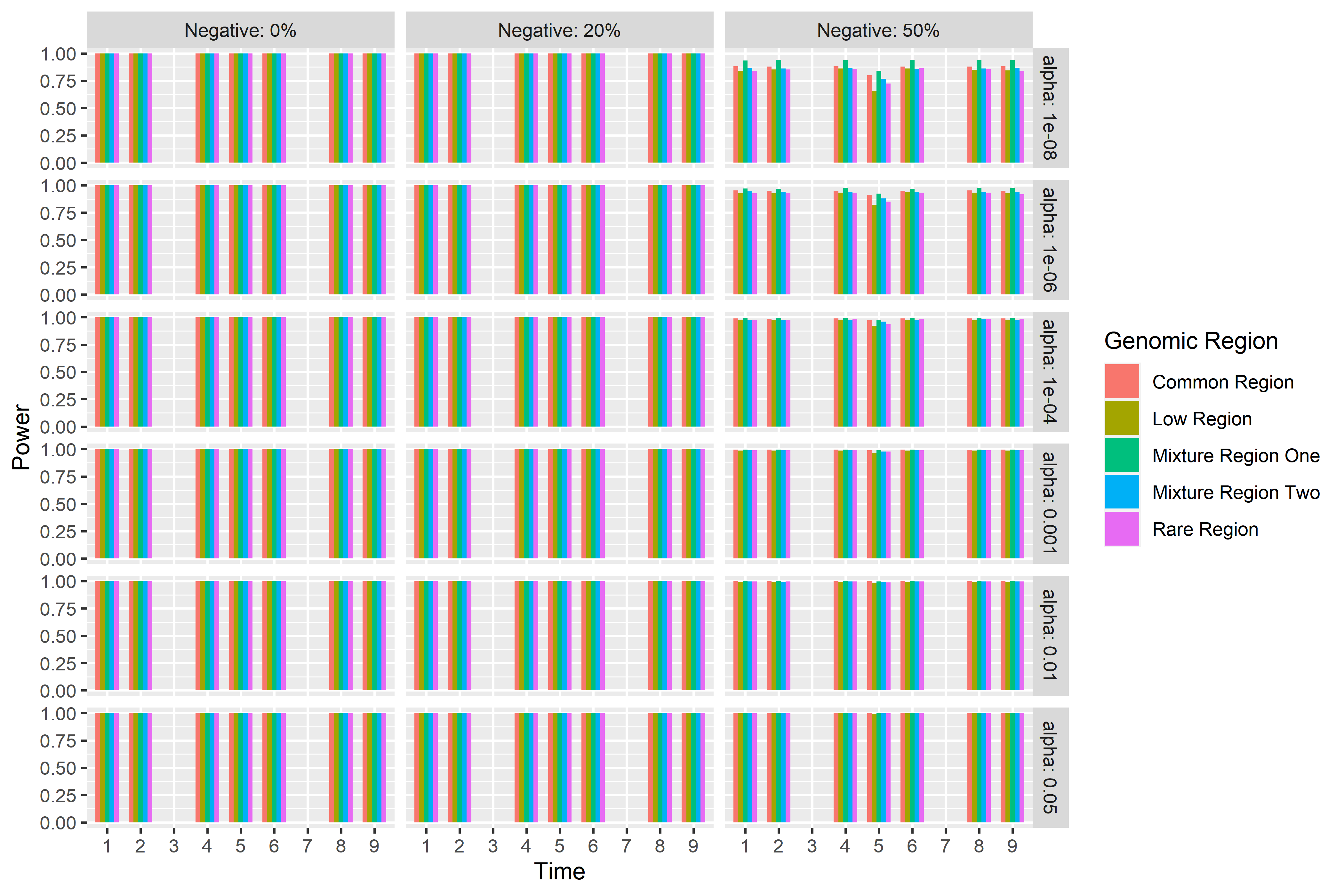

Supplement: Supplementary file 1 [file DataSheet1.ZIP › data in brief/S6/Sample 1500(Case2), c is 7 and the proportion of causal variants is 4%.png]

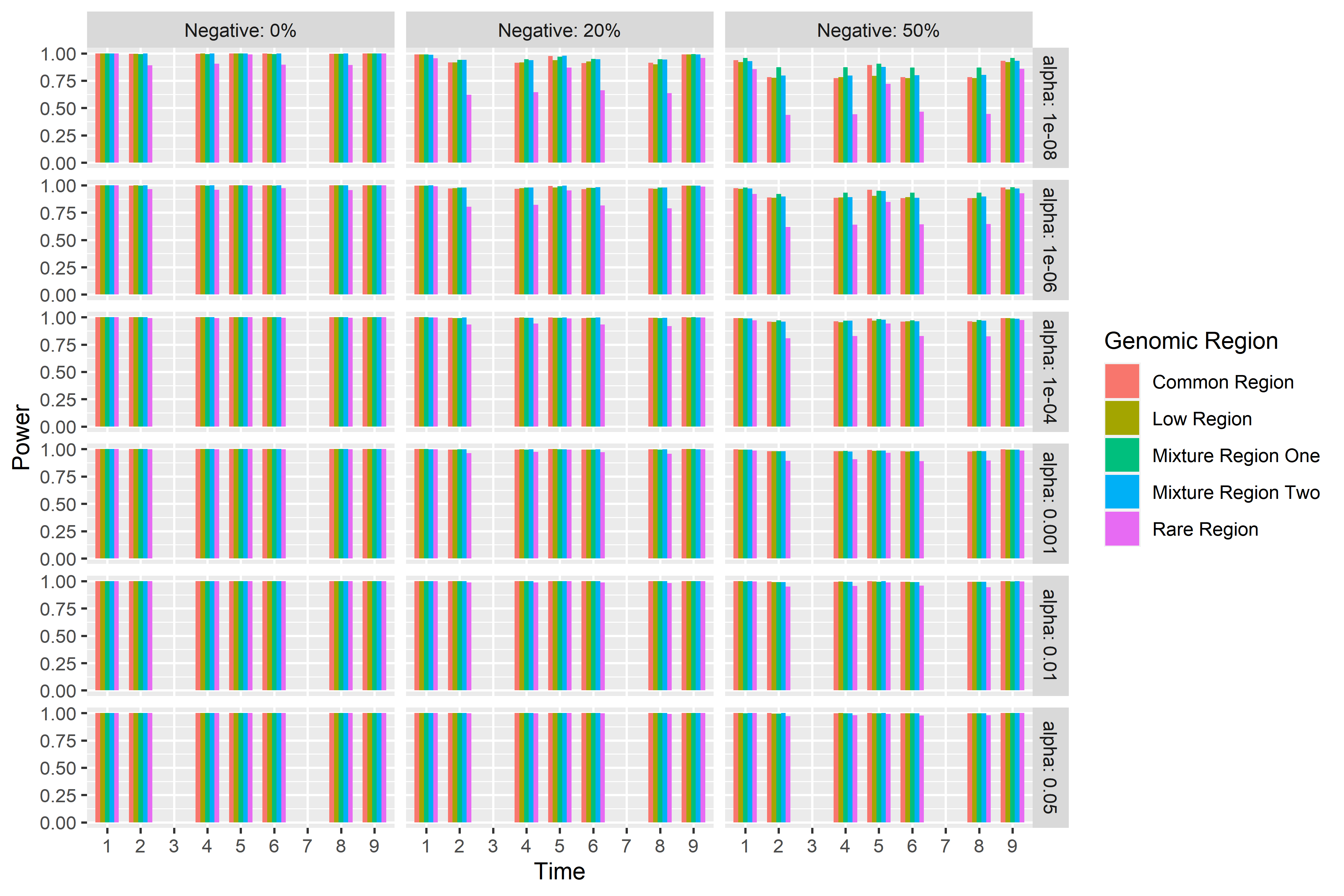

Supplement: Supplementary file 1 [file DataSheet1.ZIP › data in brief/S6/Sample 2000(Case2), c is 3 and the proportion of causal variants is 1%.png]

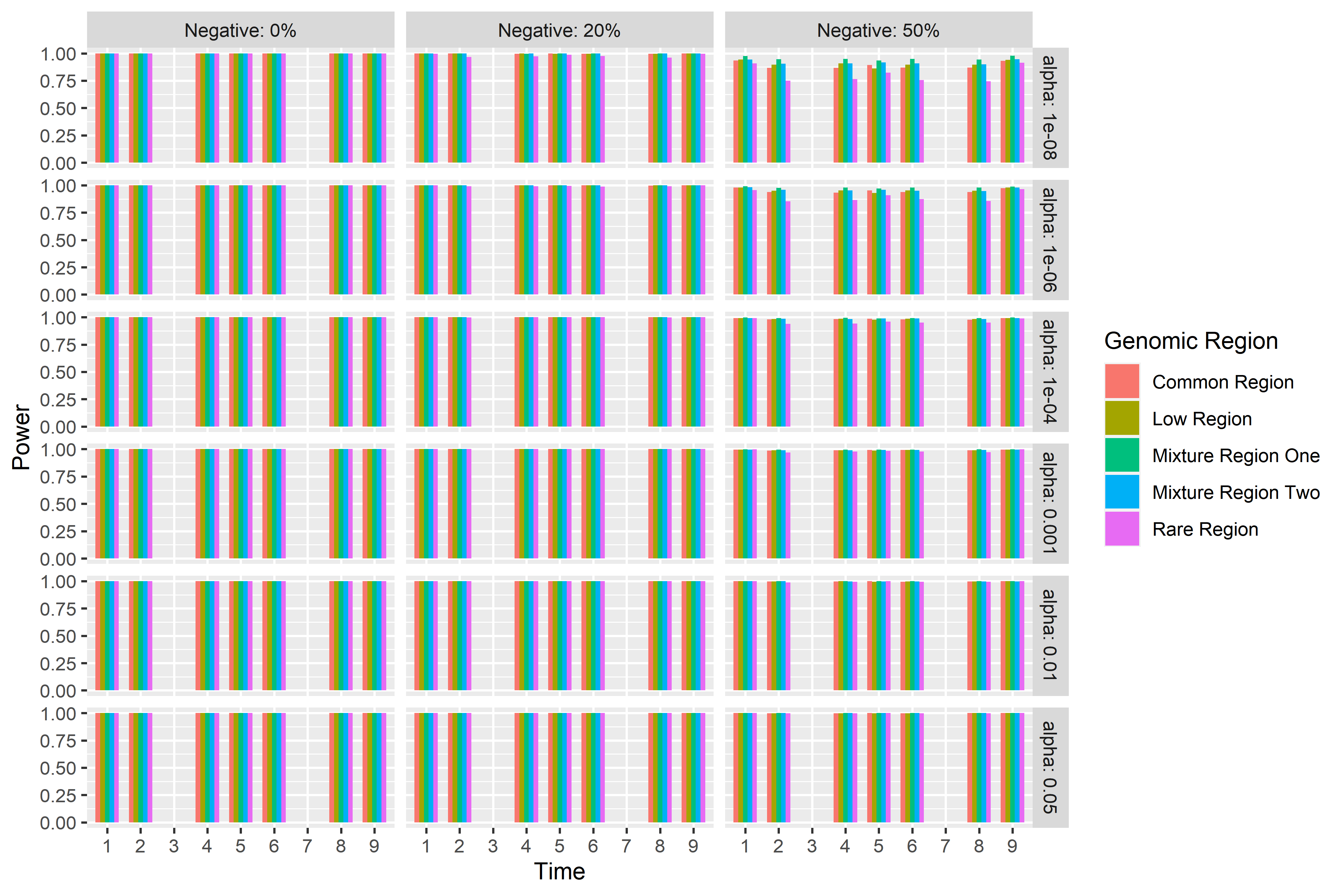

Supplement: Supplementary file 1 [file DataSheet1.ZIP › data in brief/S6/Sample 2000(Case2), c is 3 and the proportion of causal variants is 2%.png]

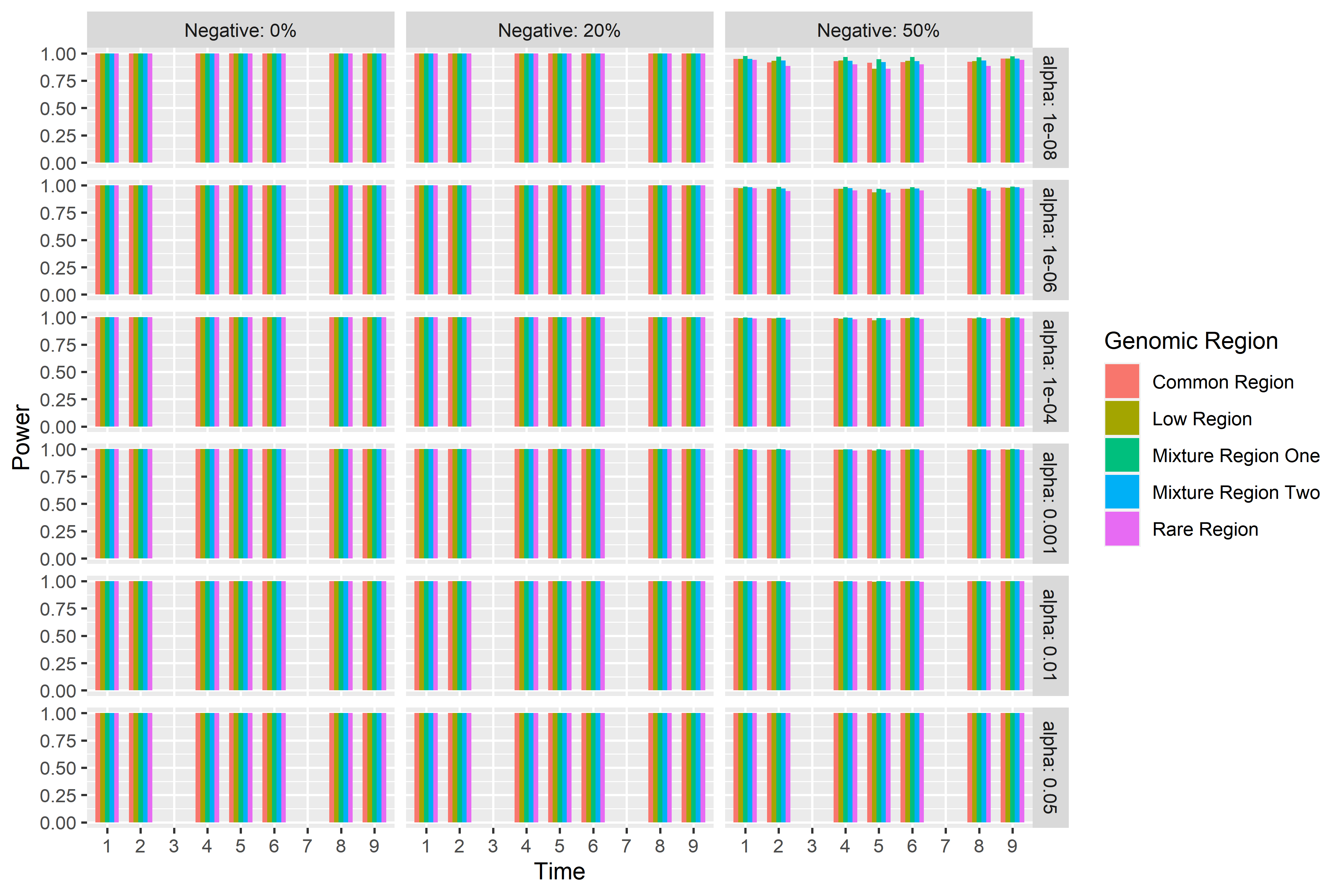

Supplement: Supplementary file 1 [file DataSheet1.ZIP › data in brief/S6/Sample 2000(Case2), c is 3 and the proportion of causal variants is 4%.png]

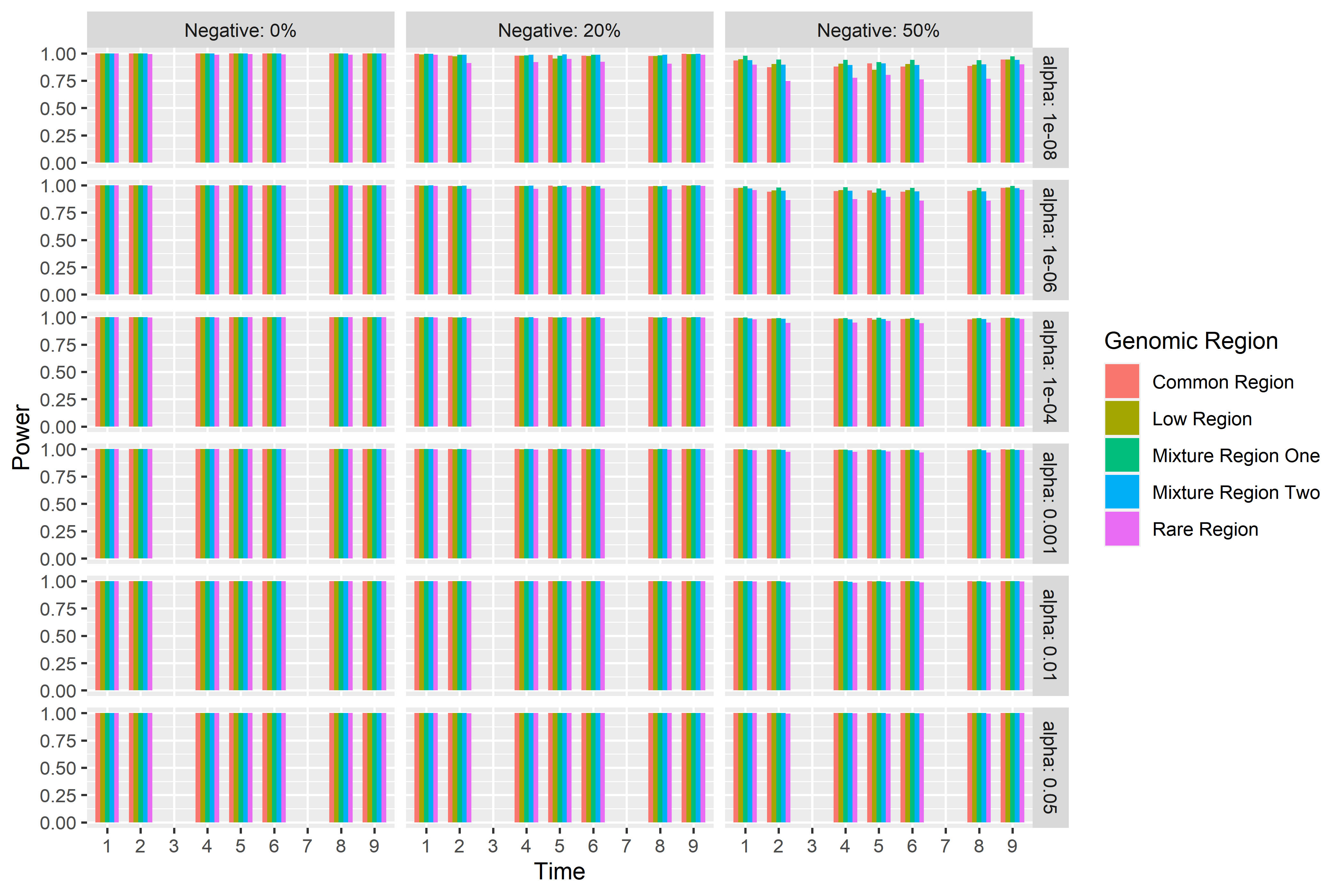

Supplement: Supplementary file 1 [file DataSheet1.ZIP › data in brief/S6/Sample 2000(Case2), c is 5 and the proportion of causal variants is 1%.png]

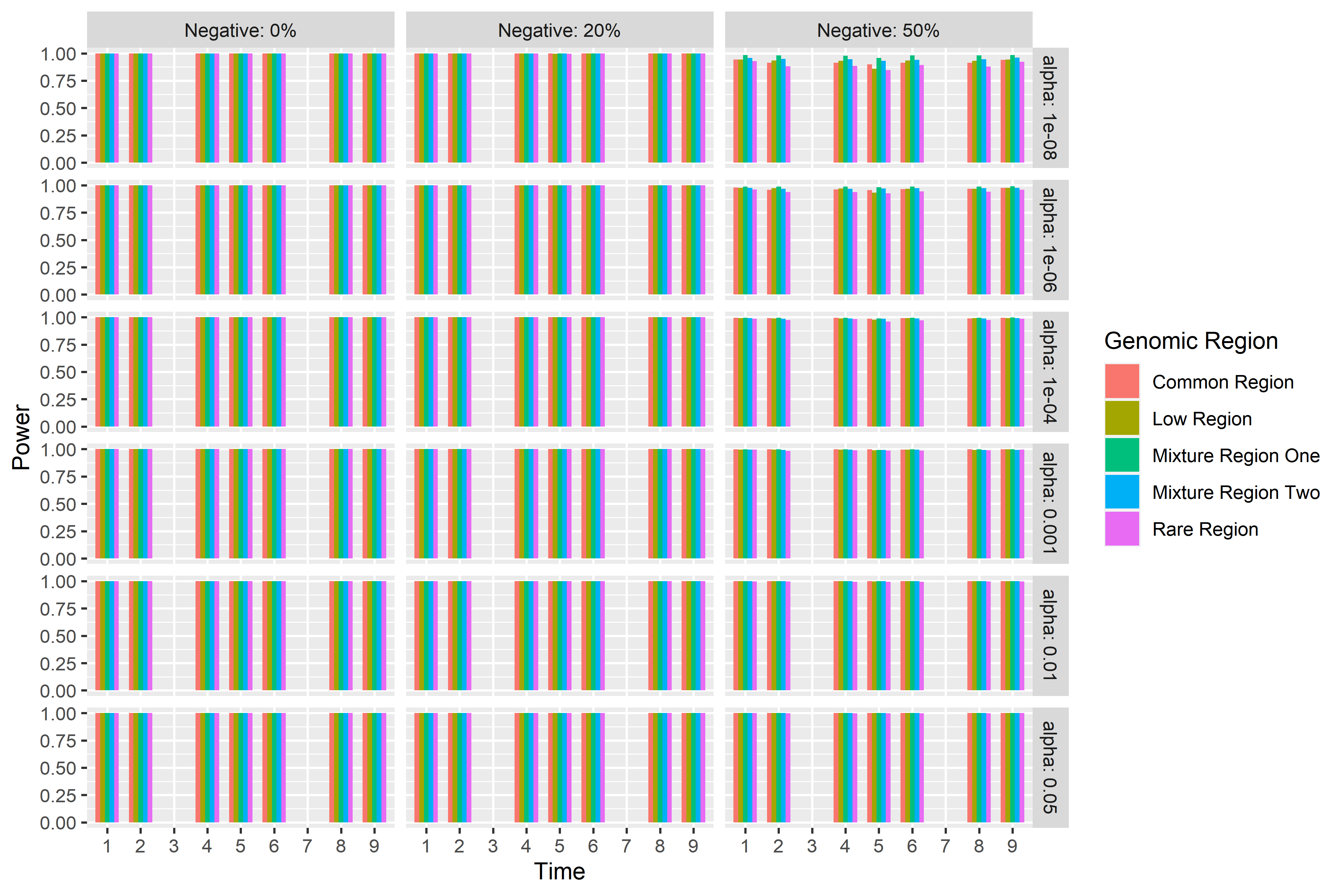

Supplement: Supplementary file 1 [file DataSheet1.ZIP › data in brief/S6/Sample 2000(Case2), c is 5 and the proportion of causal variants is 2%.png]

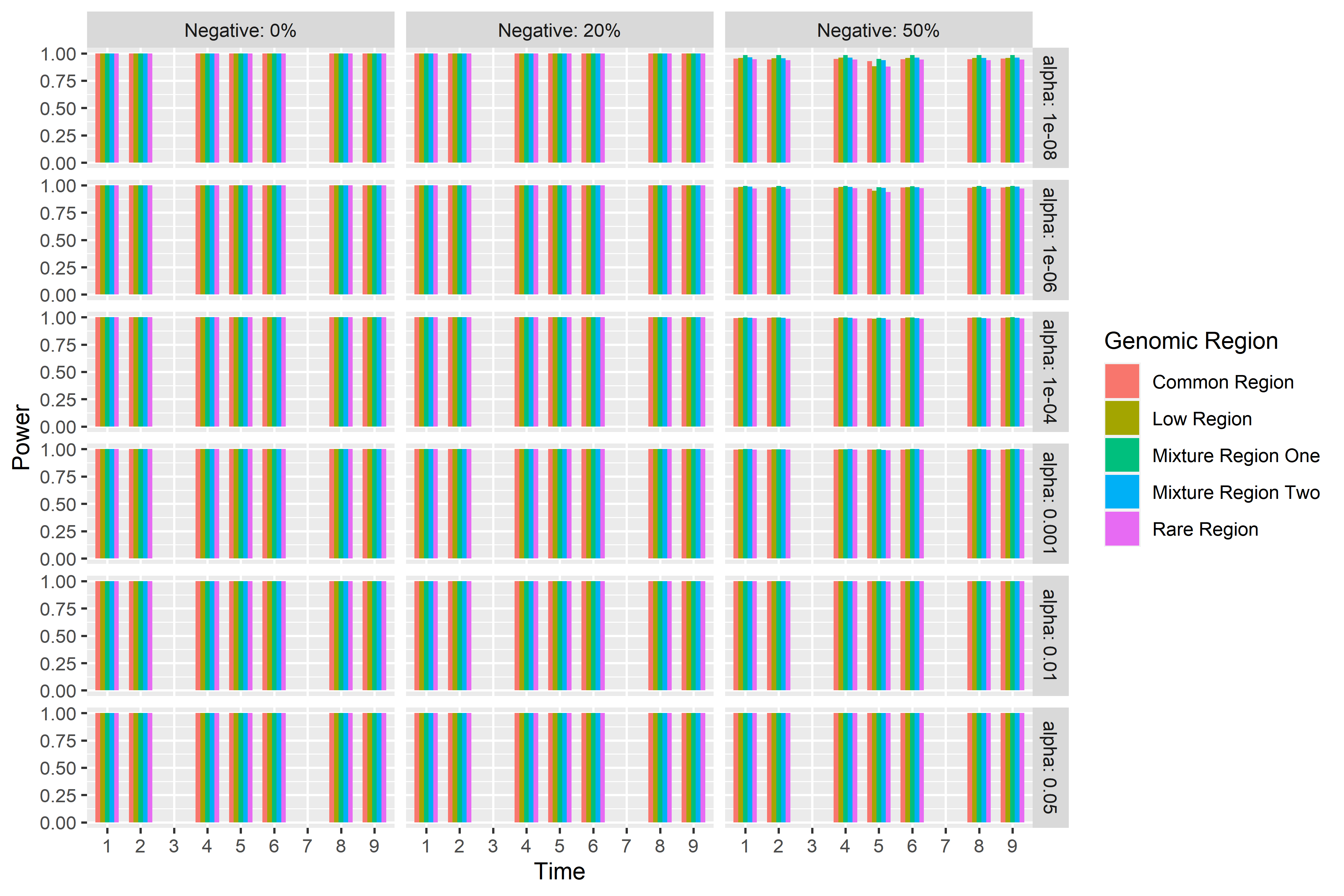

Supplement: Supplementary file 1 [file DataSheet1.ZIP › data in brief/S6/Sample 2000(Case2), c is 5 and the proportion of causal variants is 4%.png]

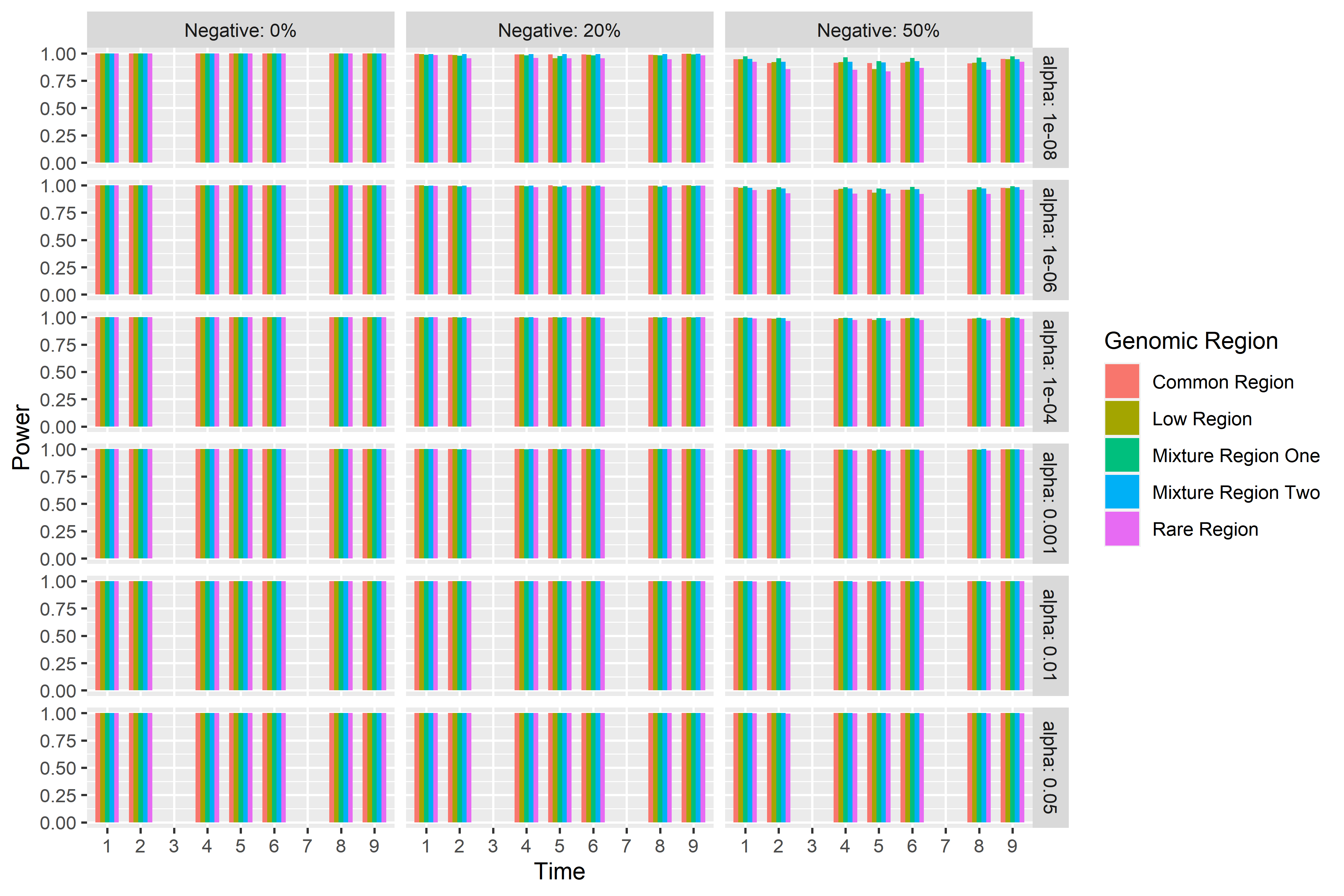

Supplement: Supplementary file 1 [file DataSheet1.ZIP › data in brief/S6/Sample 2000(Case2), c is 7 and the proportion of causal variants is 1%.png]

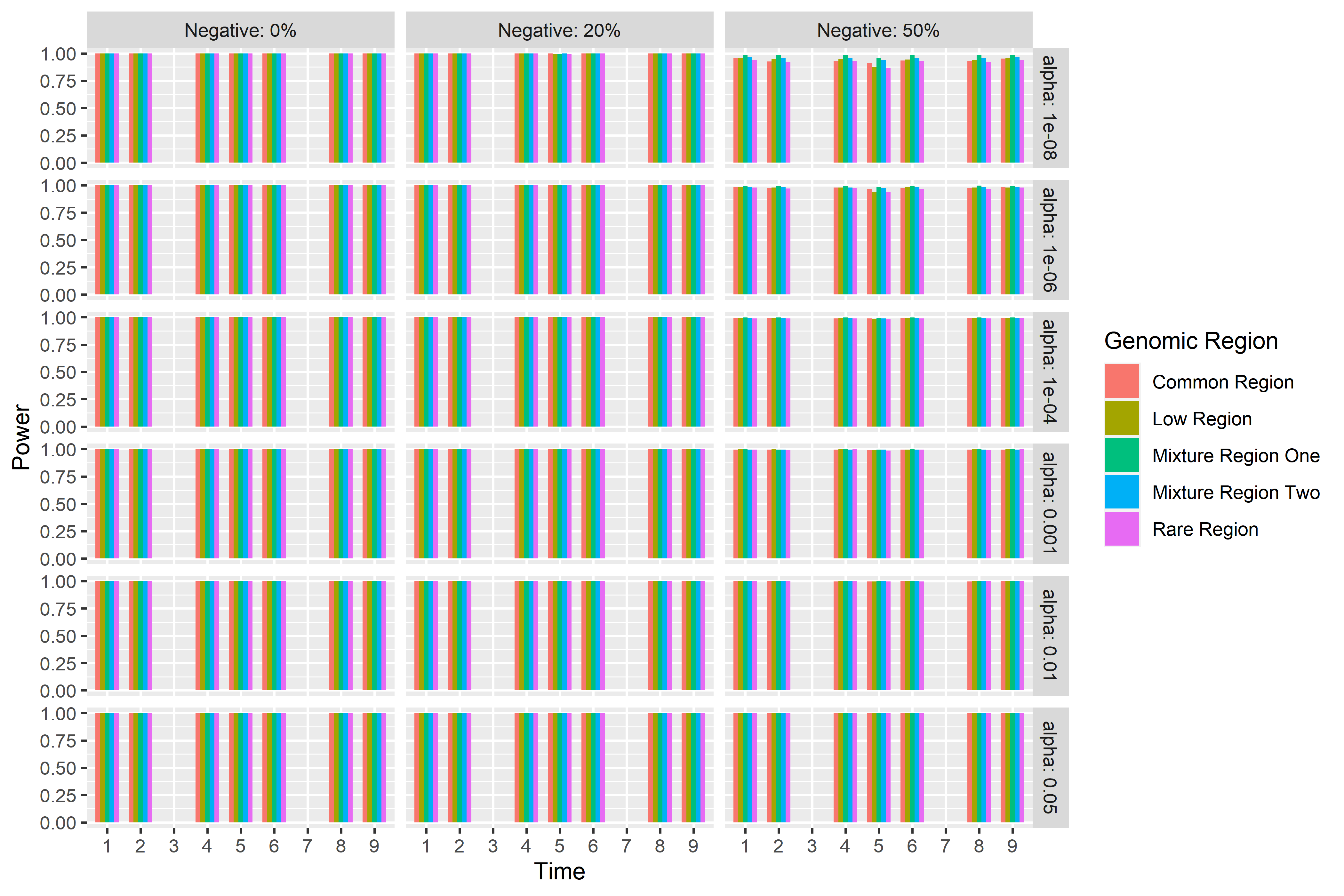

Supplement: Supplementary file 1 [file DataSheet1.ZIP › data in brief/S6/Sample 2000(Case2), c is 7 and the proportion of causal variants is 2%.png]
